# Time-Dynamic AI Models to Predict Quality of Life in Patients With Breast Cancer: Development and Validation Study Using the EORTC BALANCE Cohort

**DOI:** 10.2196/81424

**Published:** 2026-04-30

**Authors:** Niclas J Hubel, Thijs G W van der Heijden, Benjamin Murauer, Belle H de Rooij, Kelly M de Ligt, Helena M Verkooijen, Sofie AM Gernaat, Meeke Hoedjes, Volker Arndt, Lonneke V van de Poll-Franse, Bernhard Holzner, Jens Lehmann

**Affiliations:** 1 Health Outcomes Research Unit, University Hospital of Psychiatry II, Medical University of Innsbruck, Anichstrasse 35, Innsbruck, 6020, Austria, 00 43 0 512 5042 3629; 2 Department of Psychosocial Research and Epidemiology, The Netherlands Cancer Institute, Amsterdam, The Netherlands; 3 Evaluation Software Development GmbH, Innsbruck, Austria; 4 Center of Research on Psychological Disorders and Somatic Diseases, Department of Medical and Clinical Psychology, Tilburg University, Tilburg, The Netherlands; 5 Department of Research and Development, Netherlands Comprehensive Cancer Organisation (IKNL), Utrecht, The Netherlands; 6 Division of Imaging and Oncology, University Medical Centre Utrecht, Utrecht, The Netherlands; 7 Center of Research on Psychological Disorders and Somatic Disorders, Department of Medical and Clinical Psychology, Tilburg University, Tilburg, The Netherlands; 8 Cancer Survivorship Outcomes and Epidemiology, German Cancer Research Center (DKFZ), Heidelberg, Germany; 9 on behalf of the EORTC Quality of Life Group

**Keywords:** machine learning, health-related quality of life, HRQoL, breast cancer, prediction modeling, patient-reported outcomes

## Abstract

**Background:**

Patients with breast cancer often experience health-related quality of life (HRQoL) impairments that remain difficult to predict on an individual level. Prediction models can aid in understanding individual survivorship trajectories. However, current prognostic models are based on fixed intervals, limiting their utility in clinical follow-up schedules.

**Objective:**

This study aimed to develop and externally validate time-dynamic machine learning (ML) models that predict clinically relevant HRQoL impairments in nonmetastatic patients with breast cancer.

**Methods:**

Using the pooled multicohort EORTC (European Organisation for Research and Treatment of Cancer) BALANCE (big data in patients with breast cancer) dataset (n=6316) containing repeated HRQoL measurements (EORTC QLQ [Quality of Life Core Questionnaire]-C30), we constructed over 70,000 patient assessment pairs. ML algorithms were trained using the earlier HRQoL assessment and clinical data to predict dichotomized impairments in QLQ-C30 domains at the later assessment between 2 weeks and 5 years ahead, reflecting the range of follow-up intervals available in the dataset. The best performing model was determined via the area under the receiver operating characteristic curve in the internal validation, and externally validated in an independent cohort of the BALANCE dataset, in which the calibration and predictive performance in risk groups (patients: postmenopause, with financial difficulties, with obesity, with 2 or more comorbidities, with lower educational status, and with frailty) were also evaluated.

**Results:**

ML models showed good discrimination (area under the receiver operating characteristic curve 0.64‐0.84) across most domains, especially for persistent symptoms such as fatigue, financial difficulties, or functioning scales. Gradient boosting models performed best, but tended to be overconfident, with poor calibration for low-prevalence symptoms such as diarrhea or constipation. Model performance varied by risk group (eg, lower education and frailty), though no group consistently performed poorly. Performance remained stable across time windows, with prior HRQoL being the strongest predictor at the respective scale level, while clinical variables such as the type of treatment were less important for prediction.

**Conclusions:**

Time-dynamic ML models can support personalized HRQoL prediction in breast cancer care. Future improvements should focus on calibration and fairness to enable equitable, clinically meaningful implementation.

## Introduction

Breast cancer remains one of the most common malignancies among women worldwide, and although survival rates have improved significantly, many patients continue to experience long-term physical, emotional, and psychosocial consequences, stemming from both the disease and its treatment [1]. These effects can have a profound impact on health-related quality of life (HRQoL) across survivorship trajectories [2,3]. Despite its clinical importance, clinicians often lack individualized insights into how HRQoL evolves across the cancer trajectory. This gap contributes to persistent unmet supportive care needs among patients with breast cancer, especially in the posttreatment phase [4]. Without clear prognostic guidance on expected HRQoL changes, opportunities for patient-centered care and shared decision-making may be missed, potentially leading to suboptimal follow-up strategies and delayed interventions [5].

Machine learning (ML) offers a promising avenue to address this gap when incorporating real-world data (RWD). Techniques such as deep learning and gradient boosting can uncover complex, nonlinear relationships within high-dimensional data and provide personalized HRQoL predictions [6]. Existing ML models in breast cancer have largely focused on traditional clinical endpoints such as survival or recurrence [7]. In contrast, HRQoL remains underexplored as an outcome, despite being, alongside overall survival, one of the top priorities for patients [8,9].

However, several key challenges remain in applying ML to HRQoL prediction. First, structured, large-scale datasets capturing longitudinal HRQoL data are scarce [7,10]. In addition, existing datasets are highly heterogeneous: clinical trial data, RWD, and observational studies differ substantially in structure, completeness, and context. While integrating multiple data sources may enhance generalizability, it also introduces complex challenges related to data harmonization and standardization, making analyses more difficult [11].

A further limitation of most existing models is their reliance on fixed prediction intervals. Typically, HRQoL is predicted at standardized time points, such as 6 or 12 months posttreatment, without accounting for variability in patients’ assessment schedules or individual disease trajectories [7,12-15]. Yet in real-world practice, assessments often occur at irregular intervals shaped by patient needs and clinical routines. Static models thus fail to reflect the variability of patient journeys. Therefore, a time-dynamic prediction approach is warranted. Such a model would allow clinicians to predict HRQoL at any future point in time based on an individual’s prior trajectory, time since last assessment, and current clinical context, supporting more timely and personalized decision-making.

Finally, ML models may inadvertently exacerbate disparities in care [16-18]. Demographic and socioeconomic differences in HRQoL reporting, combined with the underrepresentation of certain populations in training data, can lead to biased predictions and inequitable care [17]. For example, models trained predominantly on data from phase III trials in high-income countries may underperform when applied to minority populations or patients in lower-resource settings, where symptom burden, health literacy, and reporting behaviors differ significantly [19]. At present, however, it remains unclear to what extent HRQoL prediction models are affected by such biases, as empirical investigations into fairness or subgroup performance in this context are still scarce.

The present study aims to develop and validate time-dynamic ML models for predicting HRQoL in patients with nonmetastatic breast cancer from the BALANCE (big data in patients with breast cancer) cohort [11]. We compare multiple ML algorithms against a baseline model and assess the impact of data heterogeneity on predictive performance. Moreover, we investigate the presence of algorithmic bias to advance fair, flexible, and clinically relevant HRQoL prediction tools for breast cancer care.

## Methods

### Study Design

In this study, we developed and externally validated ML algorithms to predict clinically important impairment in HRQoL within multiple pooled datasets. We report our findings according to the TRIPOD (Transparent Reporting of a Multivariable Prediction Model for Individual Prognosis or Diagnosis) guidelines [20] and the Guidelines for Developing and Reporting Machine Learning Predictive Models in Biomedical Research [21].

### Ethical Considerations

Ethical approval for the secondary data analysis was obtained in 2022 at the Antoni van Leeuwenhoekziekenhuis/Nederlands Kanker Instituut from the institutional review board (IRBd22-179).

For the cohorts: ethical approval for the OPTIMUM (Towards Optimal Timing and Method for Promoting Sustained Adherence to Lifestyle and Body Weight Recommendations in Postmenopausal Breast Cancer Survivors) study was obtained from the Medical Research Ethics Committee Brabant, the Netherlands (NL66913.028.18). The VERDI (Verlauf der Diagnostischen Abklärung) study was approved by the ethics committees of the University of Heidelberg and the Medical Association of Saarland, Germany. Written informed consent was obtained from all participants. The study protocol for UMBRELLA (Utrecht Cohort for Multiple Breast Cancer Intervention Studies and Long-Term Evaluation) was approved by the Institutional Review and Ethics Board of the University Medical Center Utrecht, the Netherlands. No further compensation for the original participants was provided as their identity was not known to us. Patient data was processed only in pseudonymized form.

### Dataset

**Figure 1.**
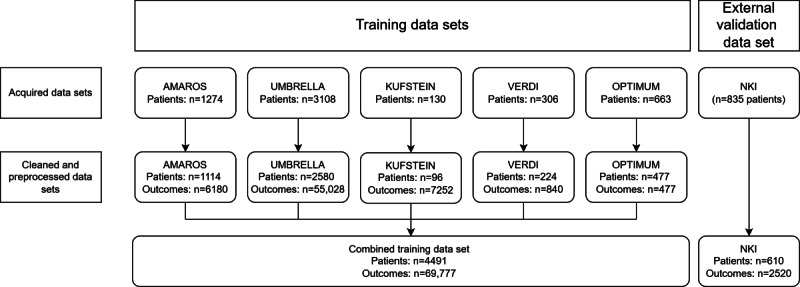
Patient flowchart. AMAROS: After Mapping of the Axilla, Radiotherapy or Surgery?; KUFSTEIN: District Hospital Kufstein cohort; NKI: Netherlands Cancer Institute; OPTIMUM: Towards Optimal Timing and Method for Promoting Sustained Adherence to Lifestyle and Body Weight Recommendations in Postmenopausal Breast Cancer Survivors; UMBRELLA: Utrecht Cohort for Multiple Breast Cancer Intervention Studies and Long-Term Evaluation; VERDI: Verlauf der Diagnostischen Abklärung.

We described the data pooling previously in more detail [11]. In brief, the BALANCE dataset comprises 6 cohorts with a total of 6316 female patients with nonmetastatic breast cancer, including trial data (EORTC [European Organisation for Research and Treatment of Cancer] AMAROS [After Mapping of the Axilla, Radiotherapy or Surgery?] [22]), RWD (Netherlands Cancer Institute [NKI] [23], district hospital Kufstein, Austria [24]), and observational studies (UMBRELLA [25], OPTIMUM [26], and VERDI [27]), with at least 2 HRQoL assessments (Figure 1). Data were collected between 2001 and 2024 and encompass patients receiving active treatment, follow-up, or survivorship care.

### Data Preparation

Data cleaning and preprocessing were conducted using R (R Foundation) [28], Jupyter Notebook, and Python (Python Software Foundation) libraries (Methods S1) in Multimedia Appendix 1 were used for statistical analyses and ML algorithms.

Features include patient characteristics and sociodemographics, comorbidities, disease and treatment parameters, as well as the items of the EORTC QLQ (Quality of Life Core Questionnaire)-C30 [29] and breast cancer module QLQ-BR23 [30]. Variables were either measured at the time of diagnosis or at the first HRQoL assessment. Cohort data were harmonized based on the common codebook (see Methods S2) in Multimedia Appendix 1. Briefly, time-related variables were coded in days since the date of diagnosis to ensure consistency across cohorts. Treatment characteristics were dichotomized into binary categories, and comorbidities were summarized as simple counts derived from available diagnostic information. Disease-related variables (eg, tumor stage and receptor status) were largely consistent across cohorts.

Features were preprocessed by one-hot encoding categorical variables (missing values were treated as distinct categories) to ensure they are represented numerically without implying an ordinal relationship and by normalizing all numeric features to 0 mean and unit variance to standardize their range, improving model performance and comparability. We removed features with 0 or near-0 variance (<0.001) from the analysis and imputed missing feature values using the iterative imputer implementation from scikit-learn. It estimates missing values in an iterated round-robin fashion as a function of other features based on a Bayesian ridge estimator [31].

To evaluate time-dynamic (ie, predicting HRQoL at every available time point using prior data and time since last assessment) predictive performance, for each patient, all possible combinations of their assessments were compiled into distinct pairs. Observations lacking either HRQoL data or the corresponding time point information were excluded. A new variable was introduced to represent the difference in days between the assessment dates within each pair. The outcome variable for each pair was defined as the value from the later assessment. For example (see Methods S3 in Multimedia Appendix 1 for an illustration), if a patient had 3 assessments, A1, A2, and A3, occurring at different time points, pairs such as (A1, A2), (A1, A3), and (A2, A3) were generated. The earlier assessment in each pair contributed its single items to the dataset, while the later assessment provided the target outcome based on the dichotomized scales (see subsection Outcomes under the Methods section). No other data from the second assessment in each pair was included in the input features. Each pair (A_m_, A_n_) is represented as a training sample (X_m_, t_m-n_, Y_n_), where X_m_ are the features of the assessment m, Y_n_ is the binary outcome variable of the respective observation, and t_m-n_ is the time difference between A_m_ and A_n_ in days. Each training sample can be interpreted as “t_m-n_ days after the patient has reported results X_m_, the patient’s outcome is Y_n_.” This approach allowed us to model changes in HRQoL over time. Figure S1 in Multimedia Appendix 1 shows the distribution of the time difference between assessment pairs in the train and test set.

### Outcomes

HRQoL was measured with the EORTC QLQ-C30 before, during, or after treatment. Symptom (fatigue, nausea and vomiting, pain, dyspnea, insomnia, appetite loss [AP], constipation, diarrhea, and financial difficulties) and functioning scales (physical, role, emotional, cognitive, and social) are reported on a 4-point Likert scale (“not at all,” “a bit,” “quite a bit,” and “very much”) and transformed to linear scores ranging from 0 to 100 scale [32]. We dichotomized the outcomes according to the established thresholds for clinical importance to indicate clinically relevant impairments, defined as scores associated with at least one of the following patient-reported concerns: limitations in daily life, need for help or care, and worries of the patient or their partner or family [33]. The dichotomization simplifies clinical interpretation through established and meaningful thresholds.

We did not adjust the dataset for class imbalance as most outcome variables were balanced (see Results section), and adjusting for class imbalance would increase the risk for overfitting.

### Algorithms

We divided our dataset into model development data (5 cohorts) and external validation data (NKI). The NKI set was chosen as it contains the most current data from clinical practice and a sufficient number of patients.

We used the scikit-learn implementations of algorithms from multiple classifier families [34] that proved effective in similar studies [6,12,35,36]. We evaluated a logistic regression with L2 regularization, an extra-trees classifier, a multilayer perceptron classifier, and a histogram-based gradient boosting classification tree (see Methods S3 in Multimedia Appendix 1 for a detailed description of libraries and GitHub for the code base). To account for the unequal contribution of patients with varying numbers of assessment pairs, we additionally incorporated sample weights inversely proportional to the number of pairs per patient. The final model was recalibrated using a probability calibration with logistic regression from scikit-learn (CalibratedClassifierCV, default settings) using a 5-fold cross-validation based on the training data. In addition, we performed post hoc recalibration on the external validation set to illustrate the extent to which context-specific adjustment can enhance model performance before deployment in a clinical setting.

### Evaluation

To assess model performance, we used internal cross-validation using a 5-fold (K=5) strategy. Given the differences in sample sizes across cohorts, the internal cross-validation was configured to keep the distribution of the cohorts within the train and test splits consistent. Thereby, the internal cross-validation was performed in a way that ensures an 80/20 train and test split and also prevents any patient overlap between train and test data.

Model performance was primarily evaluated using the area under the receiver operating characteristic curve (AUC), which is robust against class imbalance [37]. Training with AUC can provide acceptable performance at a 1/10 imbalance ratio without rebalancing [38]. Additional metrics included weighted *F*_1_-score, accuracy, balanced accuracy, and confusion matrix metrics (sensitivity, specificity, positive predictive value, and negative predictive value) based on their scikit-learn implementation using the mean and SD from all internal cross-validation iterations [34]. Accuracy reflects the overall proportion of correct predictions but can be misleading when classes are imbalanced. Balanced accuracy addresses this by averaging the recall obtained on each class, giving equal weight regardless of class frequency. The *F*_1_-score summarizes overall classification performance by balancing false positives and false negatives, with the weighted *F*_1_-score giving more weight to classes with more instances. Sensitivity (recall) quantifies the model’s ability to correctly identify patients with an impairment, whereas specificity reflects the ability to correctly identify those without impairment. Positive predictive value indicates the probability that patients predicted to have an impairment truly experience it, while negative predictive value reflects the probability that patients predicted not to have an impairment are indeed unaffected. Higher scores indicate better overall model performance across all metrics [39].

Last observation carried forward (LOCF) was used as a baseline for model comparison. LOCF serves as a straightforward method for estimating future values where patients’ HRQoL is not expected to change. These absolute values were used to compute AUC scores. It has been used in clinical trials and longitudinal studies as an imputation method or to predict future HRQoL [40,41]. Using this simple approach, therefore, provides a better baseline performance threshold compared to random chance (AUC=0.5). Furthermore, simplified logistic regression models with the prior HRQoL and the time difference as their only 2 predictors were computed to illustrate gains beyond LOCF.

Based on the internal cross-validation, we used the best-performing model for each target variable for external validation in the NKI dataset (Figure 1). Bootstrapping over 1000 iterations was used to estimate the stability of the external validation and LOCF, including 95% CIs [42].

To interpret model predictions, we applied permutation feature importance, quantifying the contribution of each feature to the trained models. Model calibration was evaluated with calibration plots, the calibration plot’s slope and intercept, and the expected calibration error (ECE), which assesses how closely predicted probabilities align with observed outcomes; good calibration is indicated by a calibration curve close to the diagonal, an intercept near zero, a slope approaching one, and a low ECE [39,43]. The ECE was based on a kernel-smoothed function using the relplot Python library [44]. Additionally, the Brier score was computed to assess the overall performance, combining discrimination and calibration, with lower values near 0 representing better performance [45].

Decision curve analyses were conducted to determine the clinical utility of the models [46]. Predicted risks may inform patient counseling, leading to heterogeneous downstream actions, such as additional assessment, supportive care, or monitoring, which differ in burden and resource use and cannot be directly compared. Evaluating net benefit across a wide threshold range (ie, the predicted risk at which a clinician would consider acting), therefore, allows assessment of model utility under varying clinical preferences and use scenarios. In this framework, the treat-all and treat-none strategies represent reference scenarios in which all patients or no patients, respectively, are considered at risk. A model is considered to have clinical utility at threshold probabilities where its decision curve yields a higher net benefit than both reference strategies, indicating more favorable trade-offs between identifying patients at risk and the use of health care resources [47].

### Model Fairness

In line with our aim of developing fair HRQoL prediction tools, and in accordance with recommendations to ensure equitable model performance across diverse populations [16], we defined risk groups within the external validation dataset to address group fairness concerns. The following risk groups were selected based on prior literature [48] and evaluated in the same manner as the full external dataset: (1) postmenopause, (2) financial difficulties (according to the thresholds for clinical importance for the financial difficulties scale [33] at baseline), (3) obesity (BMI≥30), (4) 2 or more comorbidities, (5) lower educational status (secondary education or lower), and (6) frailty (scoring according to Murugappan et al [49]). Additionally, to the previously described metrics, we evaluated true and false positive rates to screen for equalized odds disparities.

### Time-Dynamic Performance Evaluation

Further, we split the validation dataset to assess the impact of the time-dynamic aspect on model performance. As we are not relying on fixed prediction intervals, we want to ensure stable model performance across different time horizons. We evaluated long-term prediction defined as predicting outcomes more than 1 year in the future (t_m-n_ larger than 365 days), as such predictions are especially valuable for informing survivorship care planning and long-term patient management. Further, we selected all outcome pairs from within the first year after diagnosis, as this period typically encompasses active treatment, during which most changes in HRQoL are expected to occur. After the first year, HRQoL tends to stabilize, making early assessment particularly relevant for capturing clinically meaningful variation [50,51].

## Results

### Patient Characteristics

Outcome data from 6316 patients were initially acquired. After cleaning and preprocessing, 4491 patients remained in the model development dataset (Figure 1). Table 1 shows their main characteristics across all included datasets.

**Table 1.** Overview of datasets and patient characteristics at baseline. Only valid percentages are shown.

Variable	Overall	Kufstein^a^	UMBRELLA^b^	OPTIMUM^c^	VERDI^d^	AMAROS^e^	NKI^f^ (external)
Value (n)	5101	96	2580	477	224	1114	610
RWD^g^, n (%)	3987 (78.2)	N/A^h^	N/A	N/A	N/A	N/A	N/A
Country	N/A	Austria	Netherlands	Netherlands	Germany	Multiple^i^	Netherlands
Assessment date in days since diagnosis, mean (SD)	265.93 (655.79)	57.02 (141.33)	361.33 (870.33)	367.07 (35.97)	667.80 (512.69)	54.51 (146.08)	54.72 (122.77)
Number of assessment pairs per patient, mean (SD)	4.62 (3.39)	11.6 (8.76)	5.38 (4.05)	2 (0)	2.81 (1.13)	5.30 (1.86)	2.06 (0.98)
Age (years), mean (SD)	56.70 (10.81)	56.83 (13.55)	55.91 (10.54)	65.33 (7.01)	56.10 (10.88)	55.94 (10.34)	54.92 (11.71)
Postmenopause, n (%)	1535 (67.8)	N/A	N/A	477 (100)	154 (68.8)	648 (58.9)	247 (56.1)
Marital status, n (%)							
Married or living together	1491 (64.2)	6 (54.5)	573 (54.5)	349 (74.7)	151 (67.4)	N/A	412 (72)
Relationship (not married or not living together)	196 (8.4)	0 (0)	134 (12.7)	17 (3.6)	0 (0)	N/A	45 (7.9)
Divorced or split up	306 (13.2)	0 (0)	191 (18.2)	39 (8.4)	16 (7.1)	N/A	60 (10.5)
Widowed	193 (8.3)	0 (0)	80 (7.6)	45 (9.6)	45 (20.1)	N/A	23 (4)
Never married or lived together	137 (5.9)	3 (27.3)	73 (6.9)	17 (3.6)	12 (5.4)	N/A	32 (5.6)
Marital status missing	2778	87	1529	10	0	1114	38
Education status, n (%)							
Primary education or lower	112 (3.4)	N/A	40 (2)	6 (1.3)	61 (27.2)	N/A	5 (0.9)
Secondary education	660 (20.2)	N/A	490 (24.5)	98 (21)	39 (17.4)	N/A	33 (5.7)
Vocational education	907 (27.8)	N/A	435 (21.8)	162 (34.7)	117 (52.2)	N/A	193 (33.6)
University or higher (vocational) education	1586 (48.6)	N/A	1034 (51.7)	201 (43)	7 (3.1)	N/A	344 (59.8)
Education status missing	1836	96	581	10	0	1114	35
BMI, mean (SD)	25.98 (4.46)	25.20 (4.32)	N/A	26.05 (4.64)	26.03 (4.58)	25.94 (4.06)	26.10 (4.97)
Number of comorbidities, mean (SD)	1.73 (1.74)	0.33 (0.47)	N/A	2.48 (2.02)	2.25 (1.48)	N/A	1.13 (1.30)
Differentiation grade, n (%)							
Grade I	1176 (23.6)	7 (7.3)	668 (25.9)	102 (28.6)	17 (7.6)	276 (24.8)	106 (17.4)
Grade II	2239 (45)	39 (40.6)	1123 (43.5)	166 (46.5)	112 (50)	520 (46.7)	279 (45.7)
Grade III	1255 (25.2)	31 (32.3)	557 (21.6)	83 (23.2)	90 (40.2)	294 (26.4)	200 (32.8)
Grade IV	2 (0)	1 (1)	0 (0)	0 (0)	1 (0.4)	0 (0)	0 (0)
Grade unknown	307 (6.2)	17 (17.7)	232 (9)	6 (1.7)	4 (1.8)	24 (2.2)	24 (3.9)
Differentiation grade missing	120	0	0	120	0	0	0
TNM^j^ stage, n (%)							
0	196 (4.1)	0 (0)	196 (7.7)	0 (0)	0 (0)	0 (0)	0 (0)
IA	1903 (39.7)	27 (32.9)	1292 (51)	199 (64.6)	58 (33.9)	0 (0)	327 (55.2)
IB	135 (2.8)	0 (0)	135 (5.3)	0 (0)	0 (0)	0 (0)	0 (0)
IIA	1772 (36.9)	37 (45.1)	543 (21.4)	70 (22.7)	63 (36.8)	898 (80.8)	161 (27.2)
IIB	561 (11.7)	1 (1.2)	234 (9.2)	27 (8.8)	38 (22.2)	202 (18.2)	59 (10)
IIIA	176 (3.7)	17 (20.7)	102 (4)	9 (2.9)	8 (4.7)	12 (1.1)	28 (4.7)
IIIB	16 (0.3)	0 (0)	5 (0.2)	2 (0.6)	4 (2.3)	0 (0)	5 (0.8)
IIIC	38 (0.8)	0 (0)	25 (1)	1 (0.3)	0 (0)	0 (0)	12 (2)
TNM stage missing, n (%)	304	14	48	169	53	2	18
ER^k^ positive, n (%)	3049 (82.5)	N/A	2130 (83.5)	345 (86.7)	161 (77.8)	N/A	413 (76.3)
PR^l^ positive, n (%)	2548 (69.1)	N/A	1828 (71.7)	260 (65.3)	148 (71.8)	N/A	312 (58.1)
HER2NEU^m^ status, n (%)							
0	2724 (59.6)	N/A	1439 (57)	238 (60.4)	N/A	1045 (93.9)	2 (0.4)
1+	1020 (22.3)	N/A	739 (29.3)	126 (32)	N/A	68 (6.1)	87 (16.1)
2+	458 (10)	N/A	3 (0.1)	2 (0.5)	N/A	0 (0)	453 (83.6)
3+	372 (8.1)	N/A	344 (13.6)	28 (7.1)	N/A	0 (0)	0 (0)
HER2NEU status missing	527	96	55	83	224	^i^	68
Treatments, n (%)							
Surgery	4985 (98.4)	49 (51)	2572 (99.7)	440 (99.3)	223 (100)	1114 (100)	587 (96.2)
Radiotherapy	4206 (85.4)	1 (1.1)	2376 (92.1)	364 (91.5)	46 (34.8)	995 (89.3)	424 (69.5)
Chemotherapy	2779 (58.4)	94 (98.9)	1196 (46.4)	121 (78.1)	75 (36.1)	706 (63.4)	281 (46.1)
Hormonal therapy	2495 (59)	1 (1.2)	1304 (50.5)	218 (86.5)	75 (37.7)	897 (80.5)	N/A
Immunotherapy	452 (74.1)	N/A	N/A	N/A	N/A	N/A	103 (17)
Frail, n (%)	346 (7.7)	9 (9.5)	293 (11.4)	16 (3.4)	3 (1.3)	25 (2.3)	33 (5.6)
Financial difficulties, n (%)	624 (12.2)	13 (13.5)	357 (13.8)	30 (6.3)	70 (31.2)	75 (6.7)	79 (13)
Obese, n (%)	291 (15.9)	13 (18.1)	N/A	76 (16.3)	51 (23.5)	151 (14.1)	107 (19.6)
Two comorbidities or more, n (%)	444 (55.7)	0 (0)	N/A	298 (62.5)	146 (65.2)	N/A	173 (30)
Lower education status, n (%)	734 (27.3)	N/A	530 (26.5)	104 (22.3)	100 (44.6)	N/A	38 (6.6)

a Kufstein: District Hospital Kufstein cohort.

b UMBRELLA: Utrecht Cohort for Multiple Breast Cancer Intervention Studies and Long-Term Evaluation.

c OPTIMUM: Towards Optimal Timing and Method for Promoting Sustained Adherence to Lifestyle and Body Weight Recommendations in Postmenopausal Breast Cancer Survivors.

d VERDI: Verlauf der Diagnostischen Abklaerung.

e AMAROS: After Mapping of the Axilla, Radiotherapy or Surgery?

f NKI: Netherlands Cancer Institute.

g RWD: real world data.

h N/A: not applicable.

i France, Israel, Italy, Netherlands, Slovenia, Switzerland, Turkey, and United Kingdom.

j TNM: tumor, nodes, metastasis.

k ER: estrogen receptor.

l PR: progesterone receptor.

m HER2NEU: Human Epidermal Growth Factor Receptor 2 Neural.

### Outcomes

The outcome distribution in the development and validation sets after pairing HRQoL assessments showed a higher prevalence of impairments to HRQoL in the external validation set across all domains compared to the training set (Table 2). Major class imbalance (ratio of event to total number of less than 1/10) occurred in 3/14 scales (AP, constipation, and social functioning) in the training set. The event rates were higher in the external validation set.

**Table 2.** Outcome distribution (number of assessments with respective impairments) of the EORTC C30^a^ scales in the training and validation set.

	Training set (n=69,777)	External validation set (n=2520)
EORTC QLQ-C30 scale^b^	Assessments with impairments (events)^c^, n (%)	Assessments with impairments (events)^c^, n (%)
Physical functioning (PF2)	18,261 (26.2)	754 (29.9)
Role functioning (RF2)	9099 (13)	555 (22)
Emotional functioning (EF)	16,046 (23)	1051 (41.7)
Cognitive functioning (CF)	20,093 (28.8)	942 (37.4)
Social functioning (SF)	5195 (7.4)	432 (17.1)
Fatigue (FA)	14,213 (20.4)	794 (31.5)
Nausea and vomiting (NV)	9565 (13.7)	520 (20.6)
Pain (PA)	19,216 (27.5)	982 (39)
Dyspnea (DY)	19,894 (28.5)	858 (34)
Insomnia (SL)	12,245 (17.5)	605 (24)
Appetite loss (AP)	2091 (3)	124 (4.9)
Constipation (CO)	3414 (4.9)	156 (6.2)
Diarrhea (DI)	9652 (13.8)	559 (22.2)
Financial difficulties (FI)	7833 (11.2)	435 (17.3)

a EORTC QLQ-C30: European Organisation for Research and Treatment of Cancer Quality of Life Questionnaire Core 30.

b The global health status scale was not analyzed as there is no published threshold available.

c Events defined as scores below the thresholds for clinical importance indicate clinically relevant impairments according to Giesinger et al [33].

### Model Development and Internal Validation

**Figure 2.**
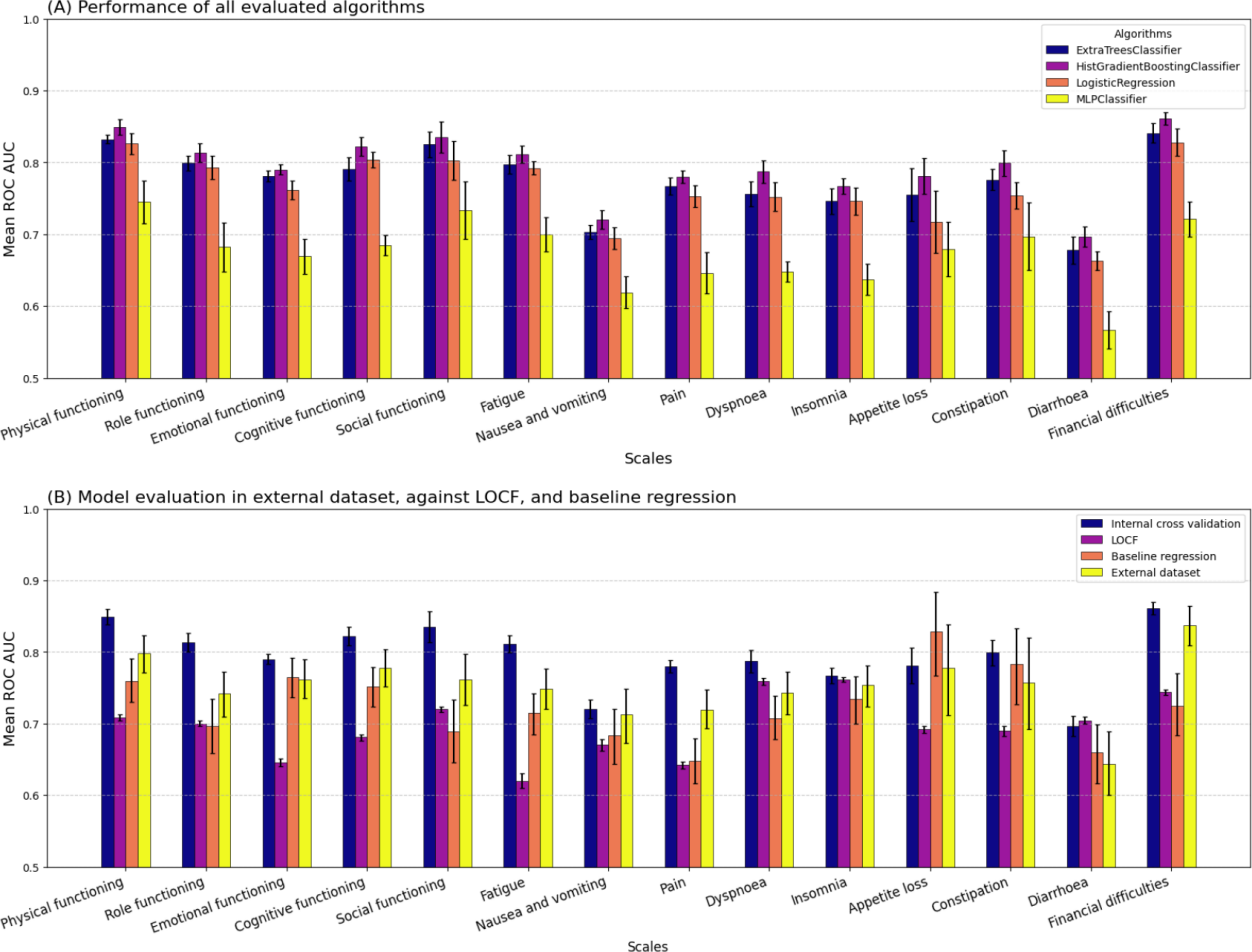
Evaluation of models trained to predict impairments in HRQoL. Error bars: SD, for LOCF and external validation: 95% CI. Full data available in Tables S1 and S2 in Multimedia Appendix 1. HRQoL: health-related quality of life; LOCF: last observation carried forward; ROC AUC: area under the receiver operating characteristic curve.

Figure 2A illustrates the performance of the logistic regression, extra-trees classifier, multilayer perceptron classifier, and a histogram-based gradient boosting classification tree for the prediction of impaired HRQoL at the next available assessment. Across all evaluated QLQ-C30 domains, the histogram-based gradient boosting classification tree showed the highest AUC, and all algorithms outperformed the LOCF baseline (Figure 2B). AUC values for the baseline regression models lay between 0.648 (95% CI 0.616‐0.679; pain) and 0.829 (95% CI 0.767‐0.884; AP), see Figure 2B for a comparison. A comprehensive list of evaluation metrics can be found in Table S1 in Multimedia Appendix 1.

From all QLQ-C30 domains, functional scales had a higher AUC than single-item symptom scales (dyspnea, insomnia, AP, constipation, and diarrhea). Except for financial difficulties, which showed the highest AUC overall (0.861, SD 0.009).

### External Validation

The external validation with the NKI yielded good discriminatory ability (Figure 2B and Table S2 in Multimedia Appendix 1). The highest AUC (0.837, 95% CI 0.809, 0.864) could be observed in the financial difficulties scale, whereas the diarrhea scale yielded the lowest AUC (0.643, 95% CI 0.600, 0.689).

Model calibration varied between the scales (Figure S2) in Multimedia Appendix 1. We observed the worst calibration in the single-item symptom scales (AP, constipation, and diarrhea), better calibration in the functioning scales, and the best calibration in fatigue and physical functioning. Generally, models for scales containing more items were better calibrated. Calibration slopes were below the ideal value of 1 for all models (Table S2) in Multimedia Appendix 1, ranging from 0.089 (constipation; 95% CI 0.063, 0.116) to 0.286 (fatigue; 95% CI 0.259, 0.313), indicating overconfident predictions. Post hoc recalibration improved discrimination (AUCs 0.975‐0.995) as well as calibration based on visual inspection (Table S5 and Figure S3) in Multimedia Appendix 1.

Across all domain models, the individual HRQoL items from the previous assessment, the time of assessment, and treatment start dates were among the most important features (Figure S4) in Multimedia Appendix 1. The type of treatment, other clinical variables, and diagnostic characteristics or comorbidities had little influence on the models’ performance.

Decision curves are shown in Figure S5 in Multimedia Appendix 1, and most models (exceptions: social functioning, AP, and diarrhea) proved to be superior compared to a “treat all” scenario for risk thresholds between 0.2 and 0.6.

### Model Fairness

**Figure 3.**
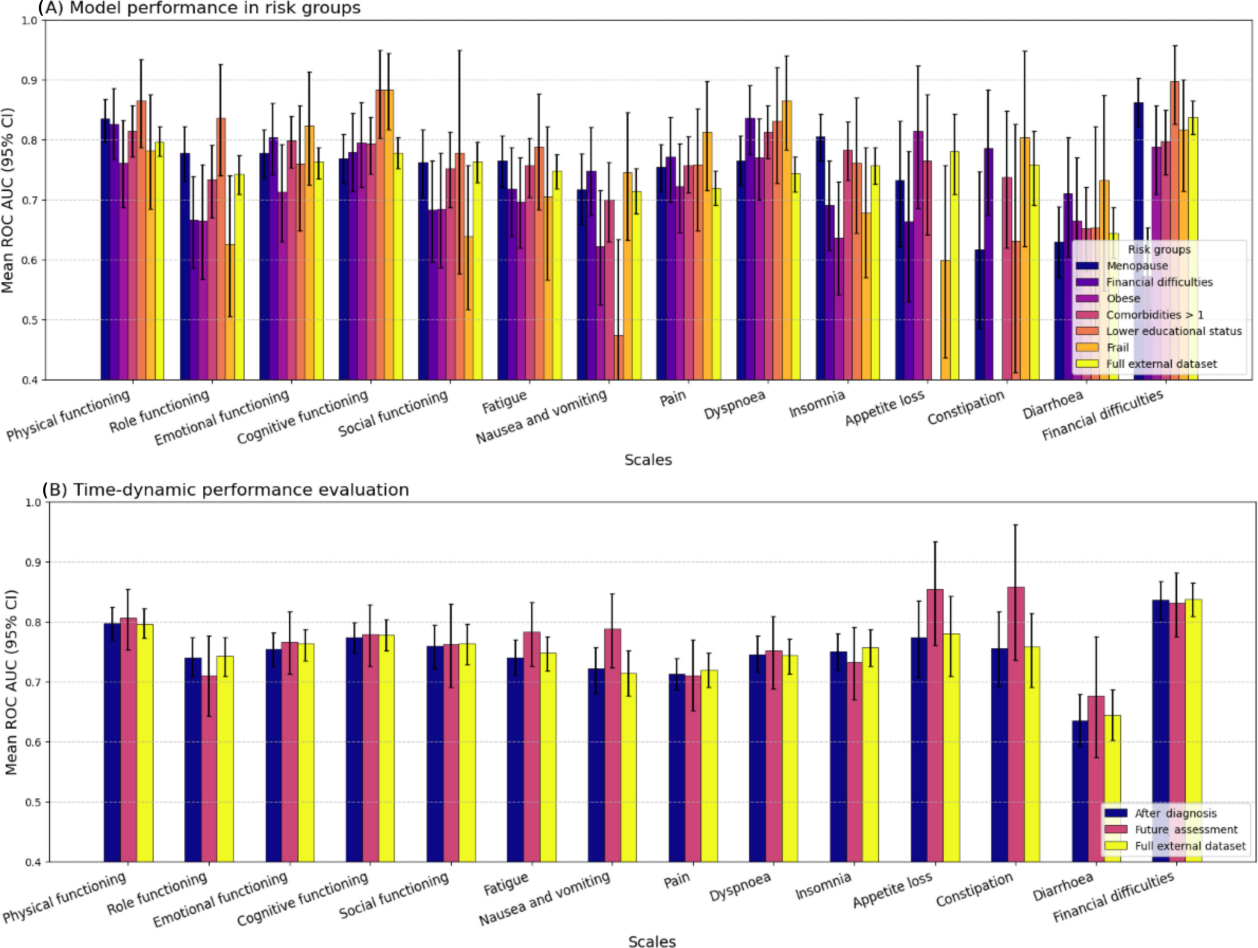
Model performance in risk groups and time-dynamic performance compared to the external validation. For (B) after diagnosis: all assessments within the first year after diagnosis; future assessment: all assessments more than 1 year in the future. Full data available in Table S4 in Multimedia Appendix 1. ROC AUC: area under the receiver operating characteristic curve.

The proportion of events in the risk groups was almost always higher than in the external validation set (Table S3) in Multimedia Appendix 1. Figure 3A illustrates model performance in risk groups selected from the external validation set in comparison with the whole set (see Table S3 in Multimedia Appendix 1 for risk group distributions).

The discrimination power within risk groups varied between domains, depending on specific risk group-scale combinations (Table S4) in Multimedia Appendix 1. The lower education status risk group showed the highest (financial difficulties: 0.897, 95% CI 0.825, 0.956) as well as the lowest (nausea and vomiting: 0.473, 95% CI 0.314, 0.633) AUC over all.

In the postmenopausal and obese risk groups, AUC CIs consistently overlapped with those of the full dataset, indicating similar model performance. This was largely consistent for other risk groups, except for the following cases: multiple comorbidities (pain and dyspnea), financial difficulties, frailty (AP and role functioning), and lower educational status (nausea and vomiting, and constipation), where reduced performance was observed.

Additionally, the predictive performance differed within some scales more than in others. For instance, in the pain scale, the AUCs were relatively homogenous, whereas the constipation scale had a high variability in AUCs. Model calibration was poor with calibration slopes across all targets and risk groups below the ideal value of 1 (Table S4) in Multimedia Appendix 1. Due to the low number of events, we could not evaluate the calibration plots.

### Time-Dynamic Performance

We observed little impact of the time-dynamic aspect on model performance (Figure 3B). The prediction within the first year after diagnosis showed similar AUCs compared to the whole external dataset, with differences below 0.01 (Table S4) in Multimedia Appendix 1. Long-term prediction showed slightly higher AUCs in 9 of 14 cases and the greatest deterioration in role functioning (0.032).

## Discussion

### Principal Findings

This study developed the first time-dynamic machine ML model for predicting impaired HRQoL in patients with early breast cancer, using multiple cohorts from different countries and settings. The ML-based prediction models consistently outperformed LOCF and the regression baseline in 10/14 cases, with the histogram-based gradient boosting classification tree showing the best performance. The best calibration was seen in multi-item scales; however, predictions were overconfident in all domains. We introduce a novel approach to time-dynamic HRQoL prediction, enabling flexible predictions beyond fixed time points. This addresses the issue with current static models, as they cannot capture the variability of real-world HRQoL assessment schedules. It therefore allows the early detection of meaningful HRQoL impairments throughout a patient’s course of disease and provides clinicians with a tool to guide targeted interventions. Model fairness analyses revealed variation in model performance across risk groups, depending on individual domain and group combinations.

### Model Evaluation and External Validation

Overall, our models demonstrated good discriminatory performance across most HRQoL domains; however, calibration was inconsistent, particularly for scales with low outcome prevalence. Discrimination, reflected in AUC values exceeding 0.75 [52], was generally strong, confirming that the models effectively distinguished between patients with impaired HRQoL and those without. This is within the range of comparable studies, most of which used internal k-fold cross-validation (see Krepper et al [12] for an overview). Yet, for some outcomes (especially AP, constipation, and diarrhea) predicted probabilities did not consistently align with observed event rates (ie, poor calibration), limiting the clinical reliability of the models in those domains.

Accurate predictions, based on both strong discrimination and acceptable calibration, were observed for key domains such as physical, emotional, and cognitive functioning, as well as fatigue, pain, and financial difficulties. These domains might reflect more persistent problems, likely contributing to the robustness of the predictive models [53,54]. In contrast, models struggled to predict transient or low-prevalence symptoms, including diarrhea, nausea and vomiting, and constipation. These limitations reflect both the nature of the symptoms, often short-lived and influenced by acute factors [55], and the statistical challenges of modeling rare outcomes [39]. The poor calibration in these domains can be attributed to the class imbalance and the insufficient total number of events. In our external dataset, some of these outcomes (AP and constipation) occurred fewer than 200 times, a known threshold below which reliable calibration curves become difficult to estimate [43], while also corresponding to less than 10% of all observations. Here, ensemble methods such as random forests and gradient boosting tend to struggle, often underestimating probabilities at the extremes due to their inherent smoothing behavior [56]. This happens because averaging predictions from many noisy base models pulls extreme values toward the center, making it difficult to produce confident predictions near 0 or 1.

To address these limitations, we recalibrated the models using data from the specific clinical setting where they could be applied, thereby adjusting for local outcome prevalence and population characteristics. We highlighted the impact of this approach in a supplementary analysis, which improved model performance drastically.

Another potential approach is upsampling the training data to balance class distributions, which showed improved calibration when using HRQoL to predict clinical events [36]. However, this does not reflect real-world incidence rates and may distort probability calibration without improving model performance in cases with an adequate number of events. Notably, similar challenges have been reported in other HRQoL modeling efforts: for example, Adiprakoso et al [35] observed good discrimination but suboptimal calibration when predicting fatigue in patients with cancer, reinforcing the need for careful evaluation of both performance dimensions.

Despite these issues, this study adds to the growing body of literature emphasizing the value of external validation, a step still rarely undertaken in HRQoL modeling despite TRIPOD guidelines recommending distinct datasets for model evaluation [20].

In line with previous research [35,57-59], prior HRQoL was consistently among the most important predictors across all models, even over longer periods of time. Temporal variables, such as treatment start and stop dates and the timing of HRQoL assessment, also ranked highly. This was also reflected in the competitive performance of the baseline model, which included only prior HRQoL and the time between assessments. In contrast, neither specific treatment types nor clinical variables (eg, tumor stage) appeared influential for predicting the general functioning and symptom domains of the QLQ-C30. This suggests that, for broad HRQoL outcomes, being on or off treatment may matter more than which specific treatment is received. These patterns may differ for treatment-specific side effects not captured by the QLQ-C30 (eg, skin problems for new targeted agents), highlighting an avenue for future studies using more granular or treatment-targeted patient-reported outcome (PRO) measures [60].

Decision curve analysis indicated that most models provided greater net benefit than treat-all and treat-none strategies across threshold probabilities between 0.2 and 0.6, suggesting potential clinical usefulness within this range. As subsequent actions following risk identification are not clearly defined and may vary widely (see Di Meglio et al [57] for a list of suggested interventions for patients with high risk of fatigue), the models should not be interpreted as directly guiding specific interventions. Rather, given that available options are often low risk and primarily involve the use of supportive resources, the models’ main value may lie in informing patient counseling and facilitating early, shared discussions about potential support needs.

### Model Fairness and Time Dynamic Performance

Model fairness analyses revealed disparities in model performance, notably among patients with lower educational status or frailty. However, there was no single risk group with the constant worst discrimination in all domains. Our findings echo concerns about algorithmic bias in health care ML models, exacerbating existing inequities in socioeconomic status, disability, and other characteristics [61-63]. This was reflected in the increased prevalence of impaired HRQoL across the selected risk groups, which nevertheless could not always be predicted correctly. Addressing these risks requires the use of fairness-aware ML techniques, such as reweighting, adversarial debiasing, or subgroup-specific calibration, to ensure equitable performance across diverse patient populations [17]. Future work should explore these strategies to prevent the amplification of health care disparities.

Our implementation of time-dynamic prediction marks a promising methodological extension over traditional fixed-interval ML models for HRQoL. Enabled by a heterogeneous dataset, this approach better reflects real-world clinical care, where follow-up intervals vary. Importantly, we observed consistent model performance across different time spans. For long-term prediction, that is, spanning more than 1 year between assessments, this might reflect a stabilization of HRQoL. At the same time, the models also performed well during the first year after diagnosis, despite this period being marked by significant treatment-related fluctuations in HRQoL. This indicates that the models are capable of capturing both stable and dynamic phases of a patient’s trajectory, making them applicable in both contexts. In line with the ESMO (European Society for Medical Oncology) clinical practice guideline for PRO use in clinical practice, which recommends integrating PRO results into clinical care [64], such insights may inform supportive care planning by enabling the prioritization of patients who are likely to require timely interventions, while also helping patients develop more realistic expectations regarding their long-term HRQoL prognosis.

As our approach primarily served as a proof-of-concept, no direct comparisons with existing HRQoL models are currently available. Nevertheless, these encouraging results highlight the potential of time-dynamic modeling to address real-world clinical needs, warranting extension to other populations and settings. Future research is needed to compare this approach to static models.

### Strengths and Limitations

This study presents several key strengths. To our knowledge, it is the first ML approach to HRQoL prediction that incorporates time-dynamic modeling for patients with breast cancer, allowing for predictions at variable future time points rather than fixed intervals. Second, deriving our outcomes directly from anchor-based thresholds (patients reporting limitations in daily life, need for help or care, or worries of the patient or their partner or family) [33] ensures that predictions are tied to clinically meaningful impairments. As such, the models can help identify which patients are more likely to develop problems in specific HRQoL domains at selected points in the future and who may benefit from further clinical attention. Considering that PRO data are increasingly collected in routine care and that the QLQ-C30 is among the most frequently used questionnaires in electronic systems [65], our study shows that these data could be used more comprehensively to inform clinical teams and patients. Additionally, the use of a multicohort dataset, combining clinical trial data with real-world observational data, improves the model’s validity. Importantly, we used external validation using a separate cohort to evaluate generalizability and illustrated the effects of post hoc recalibration. We also explicitly assessed predictive fairness, addressing a critical and often overlooked aspect of ML in health care by examining performance across subgroups and discussing bias mitigation strategies.

Nevertheless, this study has limitations. Most patients in our dataset were Dutch, which may limit the generalizability of findings to other countries. However, reference values for early patients with breast cancer demonstrate only slight variations across Europe [66], and the models primarily rely on prior HRQoL rather than population-specific variables. This might suggest that their performance is not inherently tied to national context, and the models may be transferable to other similar countries, provided that health care systems, cultural backgrounds, and follow-up intensity are broadly comparable. Yet, model adoption into different settings must still be evaluated carefully. In addition, we are currently working to include further German and British data to expand our database and retrain our models with a more international population.

Second, even though we evaluated model performance in an RWD set and this might have reduced selection bias, there might still be an underrepresentation of certain minorities or risk groups in our data.

Furthermore, as we performed a secondary analysis on existing datasets not originally intended for this purpose, some potentially important predictors of HRQoL (eg, socioeconomic status or social support) were unavailable, which may have limited the performance of our models.

Additionally, we dichotomized the outcomes to facilitate clinical interpretation; this may have led to a loss of information and reduced predictive accuracy compared to modeling with the original continuous scale.

Further, the field of ML is evolving rapidly, and by the time results are published, newer architectures (eg, pretrained tabular foundation models [67]) may already offer improved performance. Exploring such approaches was beyond the scope of this study but represents an important direction for future research.

Finally, while our models demonstrate promise, they remain preliminary with respect to clinical implementation, especially for a few selected scales showing poor calibration for certain rare symptoms. This limitation may be attributable not only to low event rates but also to the application of a single time-dynamic modeling framework across heterogeneous phases of the patient journey, raising the possibility that more phase-specific approaches (eg, during active treatment vs posttreatment or survivorship) could improve calibration for certain symptoms.

This mirrors a systemic issue in translational implementation, where HRQoL prediction models often fail to progress beyond proof-of-concept due to a combination of technical, operational, and contextual barriers [68]. As outlined by Spencer et al [69], both general ML deployment issues and PRO-specific challenges contribute to what they refer to as the “leaky pipeline” from model development to bedside application. Moreover, compliance with regulatory frameworks, such as the European Union Artificial Intelligence Act, necessitates not only robust prospective validation in real-world settings but also demonstrable alignment with ethical, safety, and stakeholder-involvement standards.

### Conclusions

Our findings support the future integration of ML into PRO-based clinical decision support systems, while also emphasizing the need to address fairness in predictive modeling. Importantly, the predicted impairments are clinically meaningful, as they identify patients at risk of future problems derived from anchor-based thresholds and enable more timely, targeted interventions. The time-dynamic nature of the predictions further increases their clinical utility by allowing risk estimation to be aligned with clinically relevant time horizons rather than fixed follow-up intervals. Future work should focus on improving model fairness, expanding dataset diversity, and evaluating clinical implementation in real-world oncology settings.

## Supplementary material

10.2196/81424 Multimedia Appendix 1 Tables, figures, and more on the data in this paper.

## Abbreviations

AMAROS: After Mapping of the Axilla, Radiotherapy or Surgery?
AP: appetite loss
AUC: area under the receiver operating characteristic curve
BALANCE: big data in patients with breast cancer
ECE: expected calibration error
EORTC: European Organisation for Research and Treatment of Cancer
ER: estrogen receptor
ESMO: European Society for Medical Oncology
HRQoL: health-related quality of life
LOCF: last observation carried forward
ML: machine learning
NKI: Netherlands Cancer Institute
OPTIMUM: Towards Optimal Timing and Method for Promoting Sustained Adherence to Lifestyle and Body Weight Recommendations in Postmenopausal Breast Cancer Survivors
PRO: patient-reported outcome
QLQ: Quality of Life Core Questionnaire
RWD: real world data
TRIPOD: Transparent Reporting of a Multivariable Prediction Model for Individual Prognosis or Diagnosis
UMBRELLA: Utrecht Cohort for Multiple Breast Cancer Intervention Studies and Long-Term Evaluation
VERDI: Verlauf der Diagnostischen Abklärung
