# Supplementary material for: Time-Dynamic AI Models to Predict Quality of Life in Patients With Breast Cancer: Development and Validation Study Using the EORTC BALANCE Cohort
Source: J Med Internet Res. 2026 Apr 30;28:e81424. doi: 10.2196/81424 (PMC13132481; doi:10.2196/81424)
Supplement: Multimedia Appendix 1 — Tables, figures, and more on the data in this paper. [file jmir-v28-e81424-s001.pdf]

Table S1. Classifier evaluation metrics.

AUC (SD)

Target	Extra trees classifier	Histogram-based gradient boosting classification tree	Logistic Regression	Multi-layer perceptron classifier
Physical functioning	0.832 0.006	0.849 0.011	0.826 0.015	0.745 0.030
Role functioning	0.799 0.010	0.813 0.013	0.793 0.016	0.682 0.034
Emotional functioning	0.781 0.008	0.790 0.007	0.761 0.013	0.669 0.024
Cognitive functioning	0.791 0.016	0.822 0.013	0.804 0.011	0.685 0.014
Social functioning	0.825 0.018	0.835 0.022	0.803 0.027	0.733 0.040
Fatigue	0.797 0.013	0.811 0.012	0.792 0.009	0.700 0.024
Nausea and vomiting	0.703 0.010	0.720 0.013	0.694 0.015	0.619 0.022
Pain	0.767 0.012	0.780 0.009	0.753 0.015	0.646 0.029
Dyspnoea	0.756 0.017	0.787 0.016	0.752 0.020	0.648 0.014
Insomnia	0.746 0.018	0.767 0.011	0.746 0.019	0.637 0.022
Appetite loss	0.755 0.037	0.781 0.025	0.717 0.043	0.679 0.038
Constipation	0.776 0.015	0.799 0.018	0.754 0.018	0.697 0.047
Diarrhoea	0.678 0.019	0.697 0.014	0.663 0.013	0.567 0.026
Financial difficulties	0.841 0.014	0.861 0.009	0.828 0.019	0.721 0.024

Accuracy (SD)

Target	Extra trees classifier	Histogram-based gradient boosting classification tree	Logistic Regression	Multi-layer perceptron classifier
Physical functioning	0.807 0.005	0.817 0.005	0.807 0.007	0.765 0.008
Role functioning	0.877 0.009	0.876 0.006	0.874 0.007	0.843 0.014
Emotional functioning	0.791 0.012	0.797 0.010	0.791 0.014	0.739 0.011
Cognitive functioning	0.766 0.010	0.785 0.007	0.775 0.005	0.705 0.010
Social functioning	0.930 0.006	0.927 0.005	0.927 0.003	0.916 0.005
Fatigue	0.817 0.008	0.824 0.005	0.821 0.002	0.778 0.010
Nausea and vomiting	0.857 0.007	0.861 0.007	0.852 0.007	0.828 0.009
Pain	0.772 0.017	0.773 0.014	0.763 0.016	0.705 0.020

Dyspnoea	0.747 0.012	0.772 0.009	0.759 0.010	0.695 0.006
Insomnia	0.827 0.017	0.830 0.016	0.826 0.015	0.789 0.013
Appetite loss	0.969 0.004	0.968 0.006	0.966 0.003	0.967 0.005
Constipation	0.951 0.005	0.949 0.006	0.946 0.006	0.944 0.007
Diarrhoea	0.861 0.010	0.858 0.011	0.855 0.010	0.828 0.014
Financial difficulties	0.895 0.008	0.905 0.002	0.900 0.003	0.877 0.008

Balanced accuracy (SD)

Target	Extra trees classifier	Histogram-based gradient boosting classification tree	Logistic Regression	Multi-layer perceptron classifier
Physical functioning	0.689 0.016	0.731 0.019	0.707 0.011	0.676 0.031
Role functioning	0.570 0.023	0.599 0.017	0.611 0.015	0.581 0.018
Emotional functioning	0.601 0.005	0.642 0.006	0.618 0.013	0.598 0.022
Cognitive functioning	0.648 0.018	0.702 0.014	0.682 0.010	0.621 0.011
Social functioning	0.535 0.024	0.568 0.022	0.602 0.029	0.578 0.021
Fatigue	0.621 0.011	0.660 0.012	0.659 0.010	0.615 0.019
Nausea and vomiting	0.509 0.006	0.531 0.008	0.543 0.015	0.540 0.011
Pain	0.626 0.008	0.658 0.005	0.636 0.011	0.601 0.020
Dyspnoea	0.620 0.010	0.683 0.019	0.658 0.016	0.616 0.015
Insomnia	0.531 0.008	0.577 0.014	0.580 0.005	0.557 0.013
Appetite loss	0.502 0.002	0.514 0.006	0.529 0.018	0.507 0.008
Constipation	0.501 0.002	0.517 0.009	0.556 0.009	0.542 0.023
Diarrhoea	0.502 0.002	0.515 0.008	0.532 0.019	0.529 0.014
Financial difficulties	0.559 0.019	0.674 0.024	0.643 0.024	0.615 0.013

F1-Score (SD)

Target	Extra trees classifier	Histogram-based gradient boosting classification tree	Logistic Regression	Multi-layer perceptron classifier
Physical functioning	0.544 0.033	0.611 0.037	0.574 0.024	0.520 0.059
Role functioning	0.243 0.066	0.319 0.039	0.344 0.036	0.271 0.032
Emotional functioning	0.351 0.011	0.445 0.016	0.395 0.027	0.369 0.039
Cognitive functioning	0.475 0.037	0.576 0.025	0.541 0.019	0.452 0.014

Social functioning	0.127 0.079	0.227 0.059	0.305 0.071	0.241 0.047
Fatigue	0.391 0.026	0.469 0.025	0.467 0.023	0.382 0.035
Nausea and vomiting	0.046 0.019	0.130 0.025	0.175 0.043	0.186 0.026
Pain	0.423 0.016	0.493 0.010	0.450 0.019	0.408 0.034
Dyspnoea	0.419 0.021	0.543 0.036	0.499 0.030	0.447 0.030
Insomnia	0.126 0.027	0.276 0.033	0.289 0.017	0.248 0.028
Appetite loss	0.009 0.008	0.055 0.021	0.098 0.051	0.030 0.028
Constipation	0.004 0.007	0.068 0.030	0.183 0.029	0.140 0.063
Diarrhoea	0.013 0.008	0.074 0.033	0.136 0.056	0.156 0.032
Financial difficulties	0.210 0.057	0.468 0.049	0.409 0.060	0.337 0.035

Weighted F1-Score (SD)

Target	Extra trees classifier	Histogram-based gradient boosting classification tree	Logistic Regression	Multi-layer perceptron classifier
Physical functioning	0.791 0.006	0.810 0.006	0.797 0.007	0.760 0.011
Role functioning	0.843 0.014	0.852 0.011	0.854 0.009	0.828 0.017
Emotional functioning	0.757 0.015	0.777 0.009	0.763 0.015	0.728 0.014
Cognitive functioning	0.743 0.013	0.775 0.008	0.762 0.005	0.698 0.011
Social functioning	0.901 0.011	0.907 0.009	0.913 0.005	0.902 0.008
Fatigue	0.793 0.010	0.808 0.007	0.806 0.003	0.767 0.013
Nausea and vomiting	0.804 0.010	0.815 0.010	0.817 0.009	0.805 0.010
Pain	0.737 0.020	0.755 0.016	0.739 0.020	0.695 0.022
Dyspnoea	0.722 0.013	0.762 0.010	0.744 0.012	0.692 0.005
Insomnia	0.769 0.023	0.793 0.023	0.794 0.017	0.767 0.019
Appetite loss	0.956 0.007	0.956 0.008	0.956 0.006	0.955 0.007
Constipation	0.927 0.007	0.929 0.006	0.934 0.008	0.930 0.010
Diarrhoea	0.799 0.014	0.806 0.015	0.812 0.018	0.801 0.014
Financial difficulties	0.862 0.013	0.895 0.004	0.886 0.003	0.866 0.009

Table S2. External validation evaluation metrics.

Target	AUC	F1 Score	Weighted F1-score	Accuracy	Balanced accuracy	Brier score	Calibration slope	Calibration intercept	Sensitivity	Specificity	PPV	NPV	ECE
Physical functioning	0.798 [0.771, 0.823]	0.489 [0.438, 0.536]	0.72 [0.691, 0.746]	0.753 [0.729, 0.774]	0.654 [0.63, 0.677]	0.172 [0.16, 0.186]	0.218 [0.202, 0.235]	0.618 [0.585, 0.651]	0.351 [0.305, 0.396]	0.958 [0.944, 0.97]	0.807 [0.751, 0.86]	0.744 [0.717, 0.769]	0.079 [0.079, 0.079]
Role functioning	0.742 [0.71, 0.772]	0.041 [0.013, 0.078]	0.701 [0.669, 0.731]	0.788 [0.766, 0.81]	0.51 [0.501, 0.519]	0.155 [0.14, 0.169]	0.224 [0.192, 0.254]	0.643 [0.572, 0.71]	0.021 [0.006, 0.041]	0.998 [0.995, 1.0]	0.744 [0.333, 1.0]	0.789 [0.766, 0.81]	0.068 [0.068, 0.068]
Emotional functioning	0.762 [0.736, 0.79]	0.332 [0.283, 0.377]	0.618 [0.585, 0.65]	0.686 [0.661, 0.71]	0.594 [0.576, 0.612]	0.208 [0.195, 0.221]	0.248 [0.226, 0.271]	0.691 [0.652, 0.73]	0.204 [0.169, 0.239]	0.984 [0.975, 0.991]	0.887 [0.828, 0.938]	0.667 [0.641, 0.693]	0.126 [0.126, 0.126]
Cognitive functioning	0.778 [0.752, 0.804]	0.51 [0.465, 0.554]	0.694 [0.665, 0.721]	0.725 [0.699, 0.748]	0.659 [0.636, 0.68]	0.19 [0.179, 0.203]	0.223 [0.205, 0.241]	0.623 [0.591, 0.653]	0.374 [0.332, 0.418]	0.943 [0.927, 0.958]	0.803 [0.749, 0.853]	0.707 [0.681, 0.734]	0.092 [0.092, 0.092]
Social functioning	0.761 [0.726, 0.797]	0.0 [0.0, 0.0]	0.772 [0.743, 0.8]	0.844 [0.823, 0.863]	0.5 [0.5, 0.5]	0.131 [0.116, 0.149]	0.22 [0.187, 0.254]	0.794 [0.69, 0.902]	0.0 [0.0, 0.0]	1.0 [1.0, 1.0]	nan [nan, nan]	0.844 [0.823, 0.863]	0.095 [0.095, 0.095]
Fatigue	0.749 [0.72, 0.777]	0.206 [0.157, 0.25]	0.608 [0.576, 0.641]	0.696 [0.671, 0.721]	0.554 [0.538, 0.568]	0.199 [0.185, 0.212]	0.286 [0.259, 0.313]	0.724 [0.678, 0.773]	0.117 [0.087, 0.146]	0.99 [0.982, 0.995]	0.852 [0.758, 0.933]	0.688 [0.662, 0.714]	0.11 [0.11, 0.11]
Nausea and vomiting	0.713 [0.673, 0.749]	0.0 [0.0, 0.0]	0.729 [0.698, 0.757]	0.813 [0.79, 0.833]	0.5 [0.5, 0.5]	0.142 [0.127, 0.156]	0.278 [0.227, 0.328]	0.709 [0.603, 0.812]	0.0 [0.0, 0.0]	1.0 [1.0, 1.0]	nan [nan, nan]	0.813 [0.79, 0.833]	0.046 [0.046, 0.046]
Pain	0.719 [0.693, 0.747]	0.327 [0.284, 0.372]	0.564 [0.531, 0.596]	0.635 [0.608, 0.661]	0.588 [0.572, 0.606]	0.234 [0.221, 0.246]	0.259 [0.234, 0.284]	0.699 [0.664, 0.734]	0.202 [0.171, 0.236]	0.974 [0.962, 0.985]	0.859 [0.801, 0.914]	0.609 [0.582, 0.636]	0.151 [0.151, 0.151]
Dyspnoea	0.743 [0.713, 0.772]	0.442 [0.392, 0.488]	0.688 [0.661, 0.718]	0.732 [0.71, 0.755]	0.635 [0.614, 0.656]	0.192 [0.18, 0.204]	0.254 [0.227, 0.28]	0.632 [0.59, 0.67]	0.298 [0.256, 0.338]	0.972 [0.96, 0.982]	0.852 [0.796, 0.906]	0.715 [0.691, 0.74]	0.079 [0.079, 0.079]
Insomnia	0.754 [0.724, 0.781]	0.083 [0.047, 0.123]	0.659 [0.627, 0.691]	0.749 [0.726, 0.773]	0.517 [0.506, 0.529]	0.166 [0.153, 0.179]	0.231 [0.203, 0.26]	0.619 [0.564, 0.673]	0.045 [0.025, 0.067]	0.989 [0.982, 0.995]	0.571 [0.381, 0.76]	0.753 [0.73, 0.778]	0.059 [0.059, 0.059]
Appetite loss	0.778 [0.712, 0.838]	0.0 [0.0, 0.0]	0.943 [0.927, 0.958]	0.962 [0.951, 0.972]	0.5 [0.5, 0.5]	0.036 [0.027, 0.046]	0.119 [0.082, 0.164]	0.468 [0.325, 0.636]	0.0 [0.0, 0.0]	1.0 [1.0, 1.0]	nan [nan, nan]	0.962 [0.951, 0.972]	0.013 [0.013, 0.013]
Constipation	0.757 [0.692, 0.82]	0.0 [0.0, 0.0]	0.914 [0.895, 0.932]	0.942 [0.929, 0.954]	0.5 [0.5, 0.5]	0.05 [0.041, 0.061]	0.089 [0.063, 0.116]	0.326 [0.241, 0.416]	0.0 [0.0, 0.0]	1.0 [1.0, 1.0]	nan [nan, nan]	0.942 [0.929, 0.954]	0.014 [0.014, 0.014]
Diarrhoea	0.643 [0.6, 0.689]	0.0 [0.0, 0.0]	0.757 [0.729, 0.784]	0.833 [0.813, 0.852]	0.5 [0.5, 0.5]	0.135 [0.121, 0.149]	0.274 [0.197, 0.352]	0.688 [0.534, 0.839]	0.0 [0.0, 0.0]	1.0 [1.0, 1.0]	nan [nan, nan]	0.833 [0.813, 0.852]	0.033 [0.033, 0.033]
Financial difficulties	0.837 [0.809, 0.864]	0.242 [0.178, 0.308]	0.775 [0.747, 0.804]	0.828 [0.807, 0.849]	0.567 [0.546, 0.59]	0.119 [0.107, 0.132]	0.209 [0.188, 0.229]	0.651 [0.594, 0.704]	0.142 [0.101, 0.188]	0.992 [0.987, 0.997]	0.816 [0.696, 0.925]	0.828 [0.807, 0.849]	0.056 [0.056, 0.056]

Table S3. Risk group distribution in the external dataset.

Target	Full external dataset (events, %)	Menopause (events, %)	Financial difficulties (events, %)	Obese (events, %)	Comorbidities > 1 (events, %)	Lower educational status (events, %)	Frail (events, %)	Future_assessment (events, %)	After_diagnosis (events, %)
	2520 observations, 610 patients	1009 observations, 256 patients	336 observations, 101 patients	333 observations, 112 patients	667 observations, 173 patients	151 observations, 38 patients	164 observations, 58 patients	436 observations, 280 patients	2427 observations, 598 patients
Physical functioning	754 (29.9)	304 (30.1)	157 (46.7)	161 (48.3)	284 (42.6)	55 (36.4)	116 (70.7)	149 (34.2)	741 (30.5)
Role functioning	555 (22.0)	201 (19.9)	132 (39.3)	88 (26.4)	159 (23.8)	40 (26.5)	115 (70.1)	97 (22.2)	537 (22.1)
Emotional functioning	1051 (41.7)	399 (39.5)	196 (58.3)	146 (43.8)	316 (47.4)	70 (46.4)	131 (79.9)	161 (36.9)	1014 (41.8)
Cognitive functioning	942 (37.4)	339 (33.6)	191 (56.8)	145 (43.5)	278 (41.7)	60 (39.7)	138 (84.1)	179 (41.1)	906 (37.3)
Social functioning	432 (17.1)	150 (14.9)	115 (34.2)	68 (20.4)	121 (18.1)	16 (10.6)	95 (57.9)	74 (17.0)	418 (17.2)
Fatigue	794 (31.5)	316 (31.3)	188 (56.0)	162 (48.6)	276 (41.4)	60 (39.7)	138 (84.1)	142 (32.6)	766 (31.6)
Nausea and vomiting	520 (20.6)	186 (18.4)	125 (37.2)	79 (23.7)	142 (21.3)	21 (13.9)	75 (45.7)	73 (16.7)	499 (20.6)
Pain	982 (39.0)	390 (38.7)	192 (57.1)	145 (43.5)	319 (47.8)	81 (53.6)	124 (75.6)	203 (46.6)	939 (38.7)
Dyspnoea	858 (34.0)	398 (39.4)	159 (47.3)	148 (44.4)	277 (41.5)	49 (32.5)	91 (55.5)	152 (34.9)	833 (34.3)
Insomnia	605 (24.0)	276 (27.4)	113 (33.6)	88 (26.4)	212 (31.8)	32 (21.2)	82 (50.0)	122 (28.0)	579 (23.9)
Appetite loss	124 (4.9)	60 (5.9)	35 (10.4)	22 (6.6)	48 (7.2)	12 (7.9)	37 (22.6)	11 (2.5)	123 (5.1)
Constipation	156 (6.2)	31 (3.1)	40 (11.9)	10 (3.0)	52 (7.8)	9 (6.0)	35 (21.3)	22 (5.0)	151 (6.2)
Diarrhoea	559 (22.2)	222 (22.0)	92 (27.4)	85 (25.5)	166 (24.9)	32 (21.2)	48 (29.3)	51 (11.7)	550 (22.7)
Financial difficulties	435 (17.3)	159 (15.8)	264 (78.6)	59 (17.7)	117 (17.5)	38 (25.2)	66 (40.2)	103 (23.6)	402 (16.6)

Table S4. Risk group evaluation metrics.

AUC (95%-CI)

Target	Full external dataset	Comorbidities >1	Financial difficulties	Frail	Lower educational status	Menopause	Obese	Within first year after diagnosis	Long-term prediction
Physical functioning	0.796 [0.772, 0.821]	0.814 [0.771, 0.856]	0.826 [0.767, 0.885]	0.781 [0.684, 0.875]	0.864 [0.786, 0.933]	0.834 [0.796, 0.867]	0.76 [0.686, 0.832]	0.797 [0.769, 0.824]	0.806 [0.753, 0.854]
Role functioning	0.742 [0.709, 0.773]	0.733 [0.67, 0.79]	0.666 [0.585, 0.739]	0.626 [0.505, 0.74]	0.836 [0.74, 0.926]	0.777 [0.731, 0.822]	0.664 [0.567, 0.758]	0.74 [0.708, 0.774]	0.71 [0.642, 0.776]
Emotional functioning	0.763 [0.734, 0.787]	0.798 [0.753, 0.839]	0.803 [0.741, 0.86]	0.823 [0.724, 0.912]	0.759 [0.648, 0.857]	0.778 [0.736, 0.817]	0.712 [0.629, 0.792]	0.754 [0.726, 0.781]	0.766 [0.713, 0.816]
Cognitive functioning	0.778 [0.752, 0.804]	0.793 [0.742, 0.837]	0.779 [0.714, 0.844]	0.883 [0.816, 0.943]	0.882 [0.802, 0.949]	0.768 [0.727, 0.809]	0.794 [0.72, 0.862]	0.774 [0.748, 0.798]	0.779 [0.726, 0.828]
Social functioning	0.763 [0.728, 0.795]	0.751 [0.686, 0.812]	0.682 [0.596, 0.765]	0.639 [0.517, 0.757]	0.778 [0.576, 0.949]	0.762 [0.704, 0.816]	0.684 [0.587, 0.777]	0.759 [0.722, 0.794]	0.762 [0.691, 0.829]
Fatigue	0.748 [0.718, 0.775]	0.756 [0.704, 0.802]	0.718 [0.639, 0.787]	0.705 [0.566, 0.822]	0.788 [0.683, 0.876]	0.765 [0.72, 0.806]	0.695 [0.619, 0.769]	0.74 [0.711, 0.769]	0.782 [0.726, 0.832]
Nausea and vomiting	0.714 [0.676, 0.752]	0.7 [0.629, 0.762]	0.748 [0.673, 0.82]	0.745 [0.632, 0.845]	0.473 [0.314, 0.633]	0.717 [0.658, 0.776]	0.621 [0.524, 0.715]	0.721 [0.68, 0.756]	0.788 [0.723, 0.846]
Pain	0.719 [0.691, 0.747]	0.757 [0.711, 0.805]	0.771 [0.696, 0.837]	0.812 [0.715, 0.897]	0.758 [0.648, 0.851]	0.754 [0.713, 0.792]	0.721 [0.645, 0.793]	0.712 [0.686, 0.739]	0.71 [0.652, 0.769]
Dyspnoea	0.743 [0.713, 0.771]	0.813 [0.768, 0.857]	0.836 [0.775, 0.891]	0.865 [0.783, 0.94]	0.831 [0.727, 0.92]	0.764 [0.723, 0.806]	0.769 [0.7, 0.834]	0.745 [0.716, 0.776]	0.751 [0.688, 0.808]
Insomnia	0.757 [0.726, 0.786]	0.782 [0.732, 0.83]	0.69 [0.615, 0.764]	0.677 [0.57, 0.787]	0.76 [0.643, 0.87]	0.805 [0.764, 0.842]	0.636 [0.541, 0.73]	0.75 [0.719, 0.78]	0.732 [0.669, 0.791]
Appetite loss	0.78 [0.708, 0.842]	0.765 [0.641, 0.875]	0.663 [0.529, 0.78]	0.598 [0.436, 0.756]	nan [nan, nan]	0.732 [0.621, 0.831]	0.814 [0.685, 0.923]	0.774 [0.707, 0.835]	0.854 [0.76, 0.933]
Constipation	0.758 [0.691, 0.814]	0.737 [0.619, 0.847]	0.785 [0.673, 0.882]	0.803 [0.621, 0.947]	0.631 [0.411, 0.825]	0.617 [0.485, 0.746]	nan [nan, nan]	0.755 [0.692, 0.816]	0.858 [0.736, 0.962]
Diarrhoea	0.644 [0.602, 0.686]	0.651 [0.58, 0.72]	0.71 [0.605, 0.804]	0.732 [0.548, 0.873]	0.653 [0.48, 0.822]	0.629 [0.569, 0.688]	0.665 [0.549, 0.769]	0.635 [0.592, 0.679]	0.676 [0.573, 0.775]
Financial difficulties	0.837 [0.809, 0.864]	0.797 [0.741, 0.849]	0.572 [0.488, 0.653]	0.816 [0.714, 0.899]	0.897 [0.825, 0.956]	0.862 [0.822, 0.902]	0.788 [0.709, 0.857]	0.836 [0.803, 0.867]	0.831 [0.775, 0.881]

Delta AUC (95%-CI)

Target	Full external dataset	Comorbidities >1	Financial difficulties	Frail	Lower educational status	Menopause	Obese	Within first year after diagnosis	Long-term prediction
Physical functioning	0.796 [0.772, 0.821]	-0.018	-0.030	0.015	-0.068	-0.038	0.036	-0.001	-0.010
Role functioning	0.742 [0.709, 0.773]	0.009	0.076	0.116	-0.094	-0.035	0.078	0.002	0.032
Emotional functioning	0.763 [0.734, 0.787]	-0.035	-0.040	-0.060	0.004	-0.015	0.051	0.009	-0.003
Cognitive functioning	0.778 [0.752, 0.804]	-0.015	-0.001	-0.105	-0.104	0.010	-0.016	0.004	-0.001
Social functioning	0.763 [0.728, 0.795]	0.012	0.081	0.124	-0.015	0.001	0.079	0.004	0.001
Fatigue	0.748 [0.718, 0.775]	-0.008	0.030	0.043	-0.040	-0.017	0.053	0.008	-0.034
Nausea and vomiting	0.714 [0.676, 0.752]	0.014	-0.034	-0.031	0.241	-0.003	0.093	-0.007	-0.074
Pain	0.719 [0.691, 0.747]	-0.038	-0.052	-0.093	-0.039	-0.035	-0.002	0.007	0.009
Dyspnoea	0.743 [0.713, 0.771]	-0.07	-0.093	-0.122	-0.088	-0.021	-0.026	-0.002	-0.008
Insomnia	0.757 [0.726, 0.786]	-0.025	0.067	0.080	-0.003	-0.048	0.121	0.007	0.025
Appetite loss	0.78 [0.708, 0.842]	0.015	0.117	0.182	-	0.048	-0.034	0.006	-0.074
Constipation	0.758 [0.691, 0.814]	0.021	-0.027	-0.045	0.127	0.141	-	0.003	-0.100
Diarrhoea	0.644 [0.602, 0.686]	-0.007	-0.066	-0.088	-0.009	0.015	-0.021	0.009	-0.032
Financial difficulties	0.837 [0.809, 0.864]	0.04	0.265	0.021	-0.060	-0.025	0.049	0.001	0.006

Balanced accuracy (95%-CI)

Target	Full external dataset	Comorbidities >1	Financial difficulties	Frail	Lower educational status	Menopause	Obese	Within first year after diagnosis	Long-term prediction
Physical functioning	0.66 [0.638, 0.684]	0.733 [0.694, 0.773]	0.686 [0.632, 0.74]	0.685 [0.57, 0.795]	0.767 [0.683, 0.848]	0.722 [0.686, 0.758]	0.726 [0.671, 0.782]	0.659 [0.635, 0.68]	0.628 [0.583, 0.675]
Role functioning	0.51 [0.502, 0.519]	0.525 [0.505, 0.55]	0.509 [0.492, 0.531]	0.525 [0.48, 0.573]	0.5 [0.5, 0.5]	0.508 [0.5, 0.52]	0.529 [0.496, 0.567]	0.51 [0.502, 0.52]	0.523 [0.5, 0.551]
Emotional functioning	0.594 [0.575, 0.614]	0.616 [0.581, 0.653]	0.673 [0.614, 0.731]	0.804 [0.722, 0.877]	0.621 [0.55, 0.705]	0.59 [0.565, 0.615]	0.54 [0.507, 0.579]	0.592 [0.574, 0.61]	0.597 [0.561, 0.639]
Cognitive functioning	0.658 [0.636, 0.68]	0.688 [0.645, 0.734]	0.701 [0.634, 0.762]	0.752 [0.632, 0.859]	0.726 [0.634, 0.816]	0.637 [0.604, 0.672]	0.651 [0.596, 0.708]	0.653 [0.629, 0.676]	0.649 [0.605, 0.696]
Social functioning	0.5 [0.5, 0.5]	0.5 [0.5, 0.5]	0.5 [0.5, 0.5]	0.5 [0.5, 0.5]	0.5 [0.5, 0.5]	0.5 [0.5, 0.5]	0.5 [0.5, 0.5]	0.5 [0.5, 0.5]	0.5 [0.5, 0.5]

Fatigue	0.554 [0.539, 0.57]	0.594 [0.564, 0.624]	0.567 [0.522, 0.612]	0.619 [0.503, 0.722]	0.617 [0.541, 0.694]	0.548 [0.527, 0.572]	0.545 [0.517, 0.577]	0.551 [0.535, 0.566]	0.563 [0.532, 0.595]
Nausea and vomiting	0.5 [0.5, 0.5]	0.5 [0.5, 0.5]	0.5 [0.5, 0.5]	0.5 [0.5, 0.5]	0.5 [0.5, 0.5]	0.5 [0.5, 0.5]	0.5 [0.5, 0.5]	0.5 [0.5, 0.5]	0.5 [0.5, 0.5]
Pain	0.588 [0.571, 0.605]	0.642 [0.607, 0.678]	0.634 [0.576, 0.691]	0.743 [0.624, 0.847]	0.63 [0.565, 0.703]	0.612 [0.583, 0.642]	0.618 [0.566, 0.666]	0.587 [0.57, 0.606]	0.577 [0.546, 0.61]
Dyspnoea	0.635 [0.614, 0.659]	0.695 [0.651, 0.737]	0.739 [0.689, 0.787]	0.794 [0.715, 0.867]	0.63 [0.542, 0.717]	0.672 [0.64, 0.702]	0.671 [0.612, 0.725]	0.631 [0.608, 0.653]	0.648 [0.607, 0.692]
Insomnia	0.517 [0.506, 0.529]	0.543 [0.518, 0.57]	0.521 [0.485, 0.561]	0.519 [0.441, 0.601]	0.485 [0.462, 0.5]	0.511 [0.5, 0.523]	0.548 [0.51, 0.597]	0.517 [0.505, 0.529]	0.519 [0.499, 0.545]
Appetite loss	0.5 [0.5, 0.5]	0.5 [0.5, 0.5]	0.5 [0.5, 0.5]	0.5 [0.5, 0.5]	0.522 [0.5, 1.0]	0.5 [0.5, 0.5]	0.5 [0.5, 0.5]	0.5 [0.5, 0.5]	0.5 [0.5, 0.5]
Constipation	0.5 [0.5, 0.5]	0.5 [0.5, 0.5]	0.5 [0.5, 0.5]	0.5 [0.5, 0.5]	0.5 [0.5, 0.5]	0.5 [0.5, 0.5]	0.501 [0.5, 0.5]	0.5 [0.5, 0.5]	0.5 [0.5, 0.5]
Diarrhoea	0.5 [0.5, 0.5]	0.5 [0.5, 0.5]	0.5 [0.5, 0.5]	0.5 [0.5, 0.5]	0.5 [0.5, 0.5]	0.5 [0.5, 0.5]	0.5 [0.5, 0.5]	0.5 [0.5, 0.5]	0.5 [0.5, 0.5]
Financial difficulties	0.567 [0.546, 0.59]	0.557 [0.518, 0.6]	0.562 [0.5, 0.614]	0.64 [0.563, 0.718]	0.529 [0.484, 0.596]	0.578 [0.542, 0.617]	0.513 [0.48, 0.554]	0.57 [0.549, 0.593]	0.564 [0.526, 0.607]

F1-Score (95%-CI)

Target	Full external dataset	Comorbidities >1	Financial difficulties	Frail	Lower educational status	Menopause	Obese	Within first year after diagnosis	Long-term prediction
Physical functioning	0.499 [0.453, 0.549]	0.669 [0.606, 0.726]	0.582 [0.486, 0.675]	0.76 [0.673, 0.844]	0.71 [0.579, 0.827]	0.62 [0.556, 0.681]	0.648 [0.553, 0.735]	0.497 [0.446, 0.541]	0.426 [0.323, 0.53]
Role functioning	0.042 [0.014, 0.074]	0.101 [0.022, 0.185]	0.052 [0.0, 0.125]	0.141 [0.036, 0.269]	0.0 [0.0, 0.0]	0.031 [0.0, 0.076]	0.118 [0.0, 0.241]	0.044 [0.014, 0.081]	0.086 [0.0, 0.187]
Emotional functioning	0.332 [0.282, 0.381]	0.405 [0.316, 0.486]	0.592 [0.5, 0.677]	0.795 [0.701, 0.873]	0.384 [0.182, 0.581]	0.31 [0.237, 0.378]	0.173 [0.074, 0.286]	0.326 [0.277, 0.371]	0.342 [0.247, 0.444]
Cognitive functioning	0.51 [0.468, 0.553]	0.577 [0.5, 0.653]	0.702 [0.625, 0.771]	0.865 [0.797, 0.921]	0.64 [0.481, 0.772]	0.454 [0.379, 0.529]	0.482 [0.364, 0.598]	0.499 [0.453, 0.543]	0.489 [0.396, 0.579]
Social functioning	0.0 [0.0, 0.0]	0.0 [0.0, 0.0]	0.0 [0.0, 0.0]	0.0 [0.0, 0.0]	0.0 [0.0, 0.0]	0.0 [0.0, 0.0]	0.0 [0.0, 0.0]	0.0 [0.0, 0.0]	0.0 [0.0, 0.0]
Fatigue	0.207 [0.161, 0.256]	0.32 [0.233, 0.402]	0.312 [0.213, 0.411]	0.637 [0.52, 0.735]	0.401 [0.216, 0.571]	0.183 [0.111, 0.255]	0.164 [0.066, 0.267]	0.198 [0.148, 0.247]	0.222 [0.12, 0.319]
Nausea and vomiting	0.0 [0.0, 0.0]	0.0 [0.0, 0.0]	0.0 [0.0, 0.0]	0.0 [0.0, 0.0]	0.0 [0.0, 0.0]	0.0 [0.0, 0.0]	0.0 [0.0, 0.0]	0.0 [0.0, 0.0]	0.0 [0.0, 0.0]
Pain	0.326 [0.284, 0.369]	0.477 [0.404, 0.545]	0.511 [0.419, 0.6]	0.803 [0.723, 0.87]	0.433 [0.286, 0.583]	0.387 [0.312, 0.46]	0.413 [0.288, 0.521]	0.324 [0.279, 0.37]	0.286 [0.194, 0.375]
Dyspnoea	0.442 [0.395, 0.492]	0.584 [0.504, 0.656]	0.67 [0.588, 0.744]	0.782 [0.682, 0.866]	0.439 [0.233, 0.618]	0.533 [0.467, 0.592]	0.533 [0.42, 0.634]	0.432 [0.38, 0.481]	0.466 [0.37, 0.562]
Insomnia	0.083 [0.046, 0.124]	0.167 [0.084, 0.252]	0.135 [0.031, 0.253]	0.303 [0.154, 0.462]	0.0 [0.0, 0.0]	0.045 [0.011, 0.092]	0.179 [0.043, 0.328]	0.083 [0.045, 0.122]	0.088 [0.021, 0.175]
Appetite loss	0.0 [0.0, 0.0]	0.0 [0.0, 0.0]	0.0 [0.0, 0.0]	0.0 [0.0, 0.0]	0.0 [0.0, 0.0]	0.0 [0.0, 0.0]	0.0 [0.0, 0.0]	0.0 [0.0, 0.0]	0.0 [0.0, 0.0]
Constipation	0.0 [0.0, 0.0]	0.0 [0.0, 0.0]	0.0 [0.0, 0.0]	0.0 [0.0, 0.0]	0.0 [0.0, 0.0]	0.0 [0.0, 0.0]	0.0 [0.0, 0.0]	0.0 [0.0, 0.0]	0.0 [0.0, 0.0]
Diarrhoea	0.0 [0.0, 0.0]	0.0 [0.0, 0.0]	0.0 [0.0, 0.0]	0.0 [0.0, 0.0]	0.0 [0.0, 0.0]	0.0 [0.0, 0.0]	0.0 [0.0, 0.0]	0.0 [0.0, 0.0]	0.0 [0.0, 0.0]
Financial difficulties	0.242 [0.178, 0.308]	0.22 [0.101, 0.336]	0.395 [0.307, 0.487]	0.467 [0.296, 0.623]	0.131 [0.0, 0.333]	0.271 [0.164, 0.379]	0.091 [0.0, 0.217]	0.251 [0.185, 0.317]	0.236 [0.119, 0.362]

Weighted F1-Score (95%-CI)

Target	Full external dataset	Comorbidities >1	Financial difficulties	Frail	Lower educational status	Menopause	Obese	Within first year after diagnosis	Long-term prediction
Physical functioning	0.726 [0.698, 0.753]	0.733 [0.691, 0.779]	0.654 [0.578, 0.724]	0.703 [0.616, 0.796]	0.774 [0.678, 0.86]	0.774 [0.733, 0.812]	0.712 [0.638, 0.781]	0.723 [0.692, 0.748]	0.688 [0.622, 0.75]
Role functioning	0.701 [0.669, 0.733]	0.682 [0.624, 0.738]	0.497 [0.418, 0.588]	0.337 [0.22, 0.454]	0.643 [0.538, 0.753]	0.698 [0.653, 0.742]	0.673 [0.585, 0.754]	0.708 [0.678, 0.74]	0.728 [0.671, 0.79]
Emotional functioning	0.619 [0.587, 0.652]	0.593 [0.534, 0.65]	0.627 [0.554, 0.697]	0.767 [0.68, 0.844]	0.692 [0.569, 0.806]	0.613 [0.567, 0.66]	0.535 [0.443, 0.622]	0.619 [0.586, 0.651]	0.626 [0.561, 0.69]
Cognitive functioning	0.694 [0.666, 0.722]	0.718 [0.668, 0.768]	0.699 [0.632, 0.76]	0.806 [0.722, 0.881]	0.747 [0.648, 0.846]	0.694 [0.649, 0.74]	0.678 [0.601, 0.75]	0.689 [0.661, 0.719]	0.68 [0.619, 0.733]
Social functioning	0.772 [0.744, 0.8]	0.742 [0.685, 0.796]	0.576 [0.49, 0.657]	0.464 [0.346, 0.576]	0.833 [0.736, 0.916]	0.786 [0.741, 0.829]	0.692 [0.615, 0.774]	0.777 [0.751, 0.805]	0.781 [0.724, 0.839]
Fatigue	0.608 [0.576, 0.64]	0.56 [0.501, 0.619]	0.435 [0.355, 0.518]	0.593 [0.485, 0.686]	0.652 [0.533, 0.758]	0.59 [0.539, 0.64]	0.434 [0.346, 0.523]	0.608 [0.573, 0.643]	0.632 [0.568, 0.694]
Nausea and vomiting	0.728 [0.698, 0.758]	0.685 [0.629, 0.742]	0.503 [0.422, 0.582]	0.463 [0.346, 0.577]	0.735 [0.612, 0.849]	0.715 [0.668, 0.761]	0.66 [0.571, 0.743]	0.734 [0.702, 0.763]	0.752 [0.694, 0.812]
Pain	0.563 [0.53, 0.595]	0.578 [0.52, 0.634]	0.533 [0.453, 0.61]	0.75 [0.669, 0.825]	0.542 [0.421, 0.654]	0.584 [0.535, 0.633]	0.545 [0.456, 0.632]	0.565 [0.532, 0.601]	0.526 [0.452, 0.588]
Dyspnoea	0.688 [0.658, 0.718]	0.707 [0.653, 0.759]	0.723 [0.662, 0.783]	0.78 [0.694, 0.858]	0.677 [0.557, 0.787]	0.688 [0.645, 0.729]	0.663 [0.583, 0.737]	0.684 [0.649, 0.715]	0.701 [0.641, 0.756]
Insomnia	0.659 [0.624, 0.691]	0.623 [0.56, 0.684]	0.587 [0.504, 0.673]	0.499 [0.384, 0.614]	0.619 [0.493, 0.747]	0.599 [0.552, 0.649]	0.677 [0.59, 0.762]	0.663 [0.631, 0.697]	0.645 [0.581, 0.708]
Appetite loss	0.943 [0.929, 0.958]	0.931 [0.897, 0.958]	0.873 [0.813, 0.924]	0.743 [0.635, 0.853]	0.948 [0.899, 1.0]	0.924 [0.895, 0.95]	0.933 [0.886, 0.975]	0.94 [0.924, 0.956]	0.963 [0.935, 0.986]
Constipation	0.914 [0.896, 0.931]	0.885 [0.844, 0.923]	0.843 [0.777, 0.902]	0.806 [0.696, 0.901]	0.882 [0.784, 0.949]	0.945 [0.921, 0.966]	0.934 [0.886, 0.975]	0.915 [0.894, 0.932]	0.925 [0.889, 0.958]
Diarrhoea	0.757 [0.728, 0.785]	0.689 [0.632, 0.742]	0.685 [0.609, 0.762]	0.667 [0.548, 0.789]	0.801 [0.689, 0.899]	0.711 [0.666, 0.756]	0.713 [0.63, 0.79]	0.751 [0.723, 0.78]	0.826 [0.777, 0.875]
Financial difficulties	0.775 [0.746, 0.801]	0.772 [0.723, 0.822]	0.42 [0.348, 0.493]	0.612 [0.489, 0.726]	0.621 [0.492, 0.747]	0.807 [0.766, 0.845]	0.717 [0.634, 0.797]	0.791 [0.763, 0.817]	0.717 [0.652, 0.776]

Calibration slope (95%-CI)

Target	Full external dataset	Comorbidities >1	Financial difficulties	Frail	Lower educational status	Menopause	Obese	Within first year after diagnosis	Long-term prediction
Physical functioning	0.217 [0.202, 0.233]	0.219 [0.193, 0.241]	0.247 [0.205, 0.289]	0.214 [0.127, 0.302]	0.251 [0.21, 0.293]	0.23 [0.209, 0.251]	0.21 [0.168, 0.251]	0.223 [0.206, 0.24]	0.248 [0.206, 0.286]
Role functioning	0.225 [0.193, 0.256]	0.219 [0.165, 0.267]	0.244 [0.136, 0.34]	0.256 [0.027, 0.468]	0.286 [0.19, 0.375]	0.256 [0.21, 0.301]	0.187 [0.074, 0.297]	0.225 [0.191, 0.258]	0.22 [0.142, 0.3]
Emotional functioning	0.248 [0.224, 0.27]	0.278 [0.239, 0.316]	0.338 [0.275, 0.404]	0.359 [0.215, 0.494]	0.235 [0.14, 0.318]	0.256 [0.222, 0.286]	0.218 [0.136, 0.296]	0.242 [0.219, 0.266]	0.258 [0.212, 0.3]
Cognitive functioning	0.224 [0.206, 0.241]	0.218 [0.187, 0.246]	0.237 [0.186, 0.284]	0.249 [0.165, 0.335]	0.28 [0.229, 0.33]	0.218 [0.189, 0.25]	0.257 [0.2, 0.313]	0.223 [0.205, 0.24]	0.239 [0.2, 0.274]
Social functioning	0.221 [0.186, 0.257]	0.217 [0.157, 0.276]	0.276 [0.157, 0.406]	0.257 [0.034, 0.48]	0.191 [0.053, 0.312]	0.222 [0.166, 0.285]	0.232 [0.114, 0.344]	0.215 [0.18, 0.254]	0.222 [0.149, 0.294]
Fatigue	0.286 [0.259, 0.313]	0.288 [0.241, 0.333]	0.265 [0.178, 0.347]	0.335 [0.152, 0.491]	0.288 [0.202, 0.368]	0.31 [0.267, 0.352]	0.286 [0.206, 0.371]	0.281 [0.252, 0.313]	0.362 [0.303, 0.42]
Nausea and vomiting	0.279 [0.233, 0.329]	0.272 [0.182, 0.359]	0.396 [0.28, 0.507]	0.445 [0.263, 0.611]	-0.027 [-0.205, 0.154]	0.289 [0.218, 0.359]	0.218 [0.069, 0.356]	0.284 [0.233, 0.331]	0.363 [0.259, 0.462]
Pain	0.259 [0.232, 0.286]	0.269 [0.232, 0.312]	0.3 [0.224, 0.37]	0.258 [0.151, 0.369]	0.261 [0.192, 0.324]	0.286 [0.251, 0.32]	0.257 [0.189, 0.321]	0.255 [0.228, 0.281]	0.279 [0.211, 0.342]
Dyspnoea	0.254 [0.228, 0.282]	0.3 [0.257, 0.347]	0.325 [0.264, 0.386]	0.415 [0.327, 0.506]	0.341 [0.226, 0.458]	0.277 [0.24, 0.316]	0.331 [0.259, 0.401]	0.256 [0.23, 0.284]	0.283 [0.221, 0.34]
Insomnia	0.234 [0.203, 0.26]	0.27 [0.221, 0.317]	0.208 [0.128, 0.29]	0.256 [0.116, 0.394]	0.236 [0.114, 0.359]	0.291 [0.25, 0.329]	0.168 [0.066, 0.265]	0.226 [0.198, 0.256]	0.228 [0.166, 0.288]
Appetite loss	0.119 [0.079, 0.162]	0.132 [0.056, 0.214]	0.123 [0.017, 0.231]	0.154 [-0.076, 0.379]	0.152 [0.031, 0.304]	0.155 [0.068, 0.25]	0.183 [0.062, 0.331]	0.125 [0.082, 0.172]	0.103 [0.032, 0.191]
Constipation	0.089 [0.063, 0.116]	0.107 [0.053, 0.163]	0.155 [0.082, 0.231]	0.172 [0.051, 0.292]	0.055 [-0.019, 0.146]	0.022 [-0.004, 0.05]	0.072 [-0.005, 0.156]	0.086 [0.059, 0.115]	0.136 [0.072, 0.202]
Diarrhoea	0.274 [0.201, 0.345]	0.325 [0.185, 0.449]	0.427 [0.248, 0.594]	0.525 [0.092, 0.899]	0.248 [-0.032, 0.532]	0.284 [0.165, 0.405]	0.317 [0.124, 0.515]	0.263 [0.187, 0.344]	0.251 [0.114, 0.38]
Financial difficulties	0.209 [0.189, 0.23]	0.156 [0.119, 0.192]	0.084 [-0.03, 0.199]	0.298 [0.202, 0.384]	0.273 [0.193, 0.35]	0.209 [0.18, 0.239]	0.208 [0.129, 0.284]	0.203 [0.179, 0.224]	0.232 [0.19, 0.27]

Calibration intercept (95%-CI)

Target	Full external dataset	Comorbidities >1	Financial difficulties	Frail	Lower educational status	Menopause	Obese	Within first year after diagnosis	Long-term prediction
Physical functioning	0.615 [0.582, 0.648]	0.629 [0.587, 0.67]	0.678 [0.613, 0.743]	0.66 [0.562, 0.757]	0.654 [0.571, 0.739]	0.613 [0.572, 0.657]	0.649 [0.591, 0.708]	0.624 [0.591, 0.657]	0.64 [0.566, 0.713]
Role functioning	0.656 [0.58, 0.73]	0.626 [0.52, 0.736]	0.718 [0.546, 0.878]	0.773 [0.571, 0.939]	0.755 [0.521, 0.962]	0.732 [0.623, 0.84]	0.584 [0.364, 0.795]	0.647 [0.57, 0.72]	0.607 [0.448, 0.775]
Emotional functioning	0.69 [0.648, 0.73]	0.751 [0.685, 0.815]	0.737 [0.672, 0.798]	0.746 [0.677, 0.82]	0.628 [0.457, 0.776]	0.724 [0.661, 0.784]	0.685 [0.55, 0.815]	0.679 [0.639, 0.719]	0.69 [0.609, 0.768]
Cognitive functioning	0.623 [0.592, 0.653]	0.586 [0.536, 0.637]	0.57 [0.51, 0.63]	0.649 [0.541, 0.756]	0.692 [0.589, 0.792]	0.622 [0.564, 0.68]	0.689 [0.6, 0.778]	0.62 [0.588, 0.651]	0.651 [0.586, 0.712]
Social functioning	0.788 [0.677, 0.901]	0.775 [0.585, 0.958]	0.9 [0.619, 1.191]	0.876 [0.421, 1.332]	0.642 [0.227, 1.014]	0.809 [0.625, 1.016]	0.881 [0.511, 1.218]	0.77 [0.657, 0.888]	0.78 [0.551, 0.99]
Fatigue	0.724 [0.676, 0.771]	0.766 [0.698, 0.827]	0.779 [0.692, 0.861]	0.761 [0.674, 0.841]	0.723 [0.576, 0.864]	0.777 [0.704, 0.849]	0.833 [0.726, 0.94]	0.713 [0.664, 0.764]	0.793 [0.696, 0.891]
Nausea and vomiting	0.712 [0.616, 0.811]	0.696 [0.524, 0.866]	0.962 [0.77, 1.15]	0.918 [0.66, 1.146]	0.134 [-0.18, 0.469]	0.733 [0.583, 0.874]	0.643 [0.351, 0.938]	0.715 [0.609, 0.81]	0.852 [0.638, 1.062]
Pain	0.7 [0.663, 0.735]	0.738 [0.688, 0.784]	0.777 [0.721, 0.831]	0.744 [0.645, 0.832]	0.779 [0.7, 0.865]	0.736 [0.688, 0.783]	0.737 [0.659, 0.808]	0.691 [0.656, 0.729]	0.734 [0.655, 0.812]
Dyspnoea	0.631 [0.596, 0.669]	0.652 [0.597, 0.702]	0.697 [0.632, 0.757]	0.663 [0.585, 0.739]	0.64 [0.488, 0.79]	0.661 [0.614, 0.708]	0.654 [0.581, 0.718]	0.631 [0.592, 0.669]	0.631 [0.555, 0.701]
Insomnia	0.624 [0.562, 0.678]	0.691 [0.6, 0.781]	0.543 [0.423, 0.669]	0.611 [0.47, 0.745]	0.632 [0.391, 0.881]	0.763 [0.678, 0.842]	0.546 [0.348, 0.733]	0.61 [0.554, 0.668]	0.623 [0.502, 0.739]
Appetite loss	0.467 [0.318, 0.629]	0.507 [0.238, 0.811]	0.515 [0.124, 0.91]	0.663 [-0.047, 1.431]	0.556 [0.117, 1.108]	0.599 [0.277, 0.946]	0.692 [0.237, 1.24]	0.49 [0.328, 0.662]	0.392 [0.125, 0.722]
Constipation	0.326 [0.243, 0.418]	0.387 [0.218, 0.562]	0.511 [0.303, 0.738]	0.502 [0.212, 0.808]	0.239 [-0.0, 0.54]	0.106 [0.019, 0.205]	0.257 [0.018, 0.521]	0.317 [0.224, 0.416]	0.457 [0.252, 0.672]
Diarrhoea	0.688 [0.546, 0.826]	0.805 [0.541, 1.045]	0.976 [0.635, 1.294]	1.064 [0.37, 1.661]	0.609 [0.056, 1.183]	0.732 [0.503, 0.967]	0.767 [0.393, 1.148]	0.669 [0.516, 0.828]	0.584 [0.317, 0.837]
Financial difficulties	0.651 [0.597, 0.709]	0.505 [0.406, 0.605]	0.735 [0.659, 0.803]	0.756 [0.632, 0.88]	0.847 [0.622, 1.055]	0.665 [0.576, 0.751]	0.59 [0.408, 0.767]	0.626 [0.56, 0.686]	0.716 [0.606, 0.825]

False Positive Rate (95%-CI)

Target	Full external dataset	Comorbidities >1	Financial difficulties	Frail	Lower educational status	Menopause	Obese	Within first year after diagnosis	Long-term prediction
Physical functioning	0.039 [0.027, 0.052]	0.084 [0.049, 0.123]	0.065 [0.021, 0.119]	0.322 [0.154, 0.519]	0.062 [0.0, 0.137]	0.036 [0.017, 0.057]	0.055 [0.011, 0.107]	0.039 [0.026, 0.054]	0.033 [0.01, 0.059]
Role functioning	0.002 [0.0, 0.005]	0.004 [0.0, 0.011]	0.008 [0.0, 0.031]	0.028 [0.0, 0.091]	0.0 [0.0, 0.0]	0.0 [0.0, 0.0]	0.008 [0.0, 0.024]	0.002 [0.0, 0.005]	0.0 [0.0, 0.0]
Emotional functioning	0.016 [0.008, 0.025]	0.033 [0.01, 0.059]	0.109 [0.047, 0.178]	0.076 [0.0, 0.194]	0.0 [0.0, 0.0]	0.006 [0.0, 0.015]	0.018 [0.0, 0.046]	0.017 [0.008, 0.026]	0.02 [0.005, 0.041]
Cognitive functioning	0.057 [0.043, 0.073]	0.085 [0.05, 0.122]	0.263 [0.172, 0.356]	0.335 [0.143, 0.556]	0.077 [0.018, 0.161]	0.044 [0.024, 0.066]	0.036 [0.009, 0.075]	0.059 [0.044, 0.076]	0.053 [0.021, 0.086]
Social functioning	0.0 [0.0, 0.0]	0.0 [0.0, 0.0]	0.0 [0.0, 0.0]	0.0 [0.0, 0.0]	0.0 [0.0, 0.0]	0.0 [0.0, 0.0]	0.0 [0.0, 0.0]	0.0 [0.0, 0.0]	0.0 [0.0, 0.0]
Fatigue	0.01 [0.005, 0.017]	0.005 [0.0, 0.014]	0.06 [0.012, 0.122]	0.274 [0.1, 0.474]	0.038 [0.0, 0.091]	0.005 [0.0, 0.014]	0.0 [0.0, 0.0]	0.011 [0.005, 0.019]	0.0 [0.0, 0.0]
Nausea and vomiting	0.0 [0.0, 0.0]	0.0 [0.0, 0.0]	0.0 [0.0, 0.0]	0.0 [0.0, 0.0]	0.0 [0.0, 0.0]	0.0 [0.0, 0.0]	0.0 [0.0, 0.0]	0.0 [0.0, 0.0]	0.0 [0.0, 0.0]
Pain	0.026 [0.015, 0.038]	0.04 [0.016, 0.071]	0.091 [0.029, 0.167]	0.224 [0.053, 0.438]	0.026 [0.0, 0.088]	0.023 [0.007, 0.039]	0.034 [0.0, 0.076]	0.025 [0.014, 0.038]	0.017 [0.0, 0.039]
Dyspnoea	0.028 [0.018, 0.041]	0.049 [0.023, 0.079]	0.052 [0.011, 0.105]	0.105 [0.024, 0.211]	0.053 [0.0, 0.119]	0.04 [0.021, 0.061]	0.042 [0.01, 0.084]	0.03 [0.018, 0.041]	0.02 [0.005, 0.04]
Insomnia	0.011 [0.005, 0.018]	0.008 [0.0, 0.019]	0.038 [0.008, 0.075]	0.182 [0.083, 0.288]	0.03 [0.0, 0.077]	0.002 [0.0, 0.008]	0.007 [0.0, 0.023]	0.012 [0.005, 0.019]	0.009 [0.0, 0.022]

Appetite loss	0.0 [0.0, 0.0]	0.0 [0.0, 0.0]	0.0 [0.0, 0.0]	0.0 [0.0, 0.0]	0.0 [0.0, 0.0]	0.0 [0.0, 0.0]	0.0 [0.0, 0.0]	0.0 [0.0, 0.0]	0.0 [0.0, 0.0]
Constipation	0.0 [0.0, 0.0]	0.0 [0.0, 0.0]	0.0 [0.0, 0.0]	0.0 [0.0, 0.0]	0.0 [0.0, 0.0]	0.0 [0.0, 0.0]	0.0 [0.0, 0.0]	0.0 [0.0, 0.0]	0.0 [0.0, 0.0]
Diarrhoea	0.0 [0.0, 0.0]	0.0 [0.0, 0.0]	0.0 [0.0, 0.0]	0.0 [0.0, 0.0]	0.0 [0.0, 0.0]	0.0 [0.0, 0.0]	0.0 [0.0, 0.0]	0.0 [0.0, 0.0]	0.0 [0.0, 0.0]
Financial difficulties	0.008 [0.003, 0.013]	0.022 [0.009, 0.04]	0.138 [0.059, 0.238]	0.043 [0.0, 0.113]	0.017 [0.0, 0.058]	0.009 [0.002, 0.017]	0.028 [0.007, 0.056]	0.008 [0.003, 0.014]	0.013 [0.0, 0.027]

True Positive Rate (95%-CI)

Target	Full external dataset	Comorbidities >1	Financial difficulties	Frail	Lower educational status	Menopause	Obese	Within first year after diagnosis	Long-term prediction
Physical functioning	0.359 [0.316, 0.405]	0.551 [0.48, 0.623]	0.436 [0.34, 0.532]	0.692 [0.574, 0.8]	0.596 [0.444, 0.75]	0.48 [0.41, 0.546]	0.507 [0.407, 0.606]	0.356 [0.309, 0.397]	0.289 [0.208, 0.378]
Role functioning	0.021 [0.007, 0.038]	0.054 [0.011, 0.102]	0.027 [0.0, 0.068]	0.078 [0.018, 0.158]	0.0 [0.0, 0.0]	0.016 [0.0, 0.039]	0.066 [0.0, 0.14]	0.023 [0.007, 0.042]	0.045 [0.0, 0.103]
Emotional functioning	0.205 [0.168, 0.242]	0.265 [0.196, 0.332]	0.456 [0.361, 0.549]	0.684 [0.565, 0.796]	0.242 [0.1, 0.409]	0.186 [0.135, 0.236]	0.098 [0.039, 0.171]	0.2 [0.165, 0.235]	0.214 [0.145, 0.293]
Cognitive functioning	0.373 [0.335, 0.414]	0.46 [0.384, 0.542]	0.666 [0.576, 0.75]	0.84 [0.747, 0.918]	0.528 [0.371, 0.7]	0.318 [0.253, 0.387]	0.338 [0.235, 0.449]	0.365 [0.321, 0.408]	0.352 [0.271, 0.44]
Social functioning	0.0 [0.0, 0.0]	0.0 [0.0, 0.0]	0.0 [0.0, 0.0]	0.0 [0.0, 0.0]	0.0 [0.0, 0.0]	0.0 [0.0, 0.0]	0.0 [0.0, 0.0]	0.0 [0.0, 0.0]	0.0 [0.0, 0.0]
Fatigue	0.118 [0.089, 0.15]	0.193 [0.133, 0.251]	0.194 [0.123, 0.266]	0.512 [0.391, 0.623]	0.271 [0.125, 0.424]	0.102 [0.06, 0.148]	0.09 [0.034, 0.154]	0.113 [0.081, 0.145]	0.126 [0.064, 0.19]
Nausea and vomiting	0.0 [0.0, 0.0]	0.0 [0.0, 0.0]	0.0 [0.0, 0.0]	0.0 [0.0, 0.0]	0.0 [0.0, 0.0]	0.0 [0.0, 0.0]	0.0 [0.0, 0.0]	0.0 [0.0, 0.0]	0.0 [0.0, 0.0]
Pain	0.201 [0.171, 0.233]	0.324 [0.262, 0.389]	0.36 [0.28, 0.447]	0.71 [0.61, 0.808]	0.285 [0.169, 0.418]	0.247 [0.192, 0.306]	0.27 [0.174, 0.36]	0.2 [0.168, 0.235]	0.171 [0.11, 0.234]
Dyspnoea	0.299 [0.26, 0.342]	0.44 [0.361, 0.517]	0.53 [0.439, 0.622]	0.694 [0.56, 0.812]	0.313 [0.148, 0.485]	0.384 [0.321, 0.446]	0.383 [0.284, 0.483]	0.291 [0.248, 0.333]	0.316 [0.235, 0.407]
Insomnia	0.045 [0.025, 0.069]	0.093 [0.045, 0.146]	0.079 [0.018, 0.155]	0.22 [0.102, 0.357]	0.0 [0.0, 0.0]	0.024 [0.005, 0.048]	0.102 [0.023, 0.2]	0.045 [0.024, 0.068]	0.047 [0.011, 0.098]
Appetite loss	0.0 [0.0, 0.0]	0.0 [0.0, 0.0]	0.0 [0.0, 0.0]	0.0 [0.0, 0.0]	0.0 [0.0, 0.0]	0.0 [0.0, 0.0]	0.0 [0.0, 0.0]	0.0 [0.0, 0.0]	0.0 [0.0, 0.0]
Constipation	0.0 [0.0, 0.0]	0.0 [0.0, 0.0]	0.0 [0.0, 0.0]	0.0 [0.0, 0.0]	0.0 [0.0, 0.0]	0.0 [0.0, 0.0]	0.0 [0.0, 0.0]	0.0 [0.0, 0.0]	0.0 [0.0, 0.0]
Diarrhoea	0.0 [0.0, 0.0]	0.0 [0.0, 0.0]	0.0 [0.0, 0.0]	0.0 [0.0, 0.0]	0.0 [0.0, 0.0]	0.0 [0.0, 0.0]	0.0 [0.0, 0.0]	0.0 [0.0, 0.0]	0.0 [0.0, 0.0]
Financial difficulties	0.142 [0.101, 0.188]	0.137 [0.059, 0.22]	0.261 [0.191, 0.339]	0.323 [0.184, 0.472]	0.076 [0.0, 0.2]	0.165 [0.094, 0.245]	0.054 [0.0, 0.14]	0.149 [0.106, 0.194]	0.141 [0.065, 0.23]

Table S5. Post hoc recalibration metrics.

Target	AUC	F1 Score	Weighted F1-score	Accuracy	Balanced accuracy	Brier score	Calibration slope	Calibration intercept	Sensitivity	Specificity	PPV	NPV	ECE
Physical functioning	0.988 [0.984, 0.992]	0.904 [0.882, 0.923]	0.936 [0.922, 0.949]	0.936 [0.923, 0.949]	0.925 [0.908, 0.94]	0.079 [0.074, 0.083]	0.338 [0.331, 0.346]	0.571 [0.556, 0.588]	0.89 [0.86, 0.918]	0.96 [0.946, 0.971]	0.918 [0.894, 0.942]	0.945 [0.93, 0.96]	0.151 [0.151, 0.151]
Role functioning	0.984 [0.978, 0.988]	0.571 [0.514, 0.631]	0.848 [0.824, 0.872]	0.871 [0.852, 0.889]	0.7 [0.673, 0.731]	0.09 [0.083, 0.097]	0.456 [0.439, 0.473]	0.817 [0.783, 0.849]	0.402 [0.347, 0.463]	0.999 [0.997, 1.0]	0.991 [0.969, 1.0]	0.859 [0.838, 0.879]	0.135 [0.135, 0.135]
Emotional functioning	0.984 [0.979, 0.988]	0.891 [0.869, 0.912]	0.92 [0.905, 0.935]	0.921 [0.906, 0.935]	0.907 [0.889, 0.923]	0.093 [0.088, 0.098]	0.364 [0.356, 0.372]	0.564 [0.548, 0.58]	0.844 [0.811, 0.875]	0.969 [0.956, 0.98]	0.944 [0.922, 0.964]	0.91 [0.89, 0.928]	0.155 [0.155, 0.155]
Cognitive functioning	0.986 [0.982, 0.99]	0.891 [0.869, 0.913]	0.922 [0.907, 0.938]	0.924 [0.909, 0.939]	0.903 [0.885, 0.921]	0.105 [0.101, 0.11]	0.44 [0.43, 0.45]	0.599 [0.581, 0.615]	0.815 [0.781, 0.851]	0.991 [0.984, 0.996]	0.983 [0.97, 0.993]	0.896 [0.876, 0.917]	0.164 [0.164, 0.164]
Social functioning	0.981 [0.975, 0.986]	0.0 [0.0, 0.0]	0.772 [0.743, 0.8]	0.844 [0.823, 0.863]	0.5 [0.5, 0.5]	0.096 [0.086, 0.106]	0.666 [0.63, 0.699]	1.282 [1.211, 1.346]	0.0 [0.0, 0.0]	1.0 [1.0, 1.0]	nan [nan, nan]	0.844 [0.823, 0.863]	0.104 [0.104, 0.104]
Fatigue	0.985 [0.981, 0.989]	0.836 [0.805, 0.865]	0.9 [0.881, 0.918]	0.904 [0.888, 0.921]	0.86 [0.838, 0.881]	0.108 [0.103, 0.114]	0.474 [0.463, 0.485]	0.666 [0.647, 0.684]	0.725 [0.683, 0.767]	0.995 [0.99, 0.999]	0.987 [0.973, 0.997]	0.877 [0.857, 0.898]	0.16 [0.16, 0.16]
Nausea and vomiting	0.985 [0.98, 0.99]	0.651 [0.596, 0.706]	0.889 [0.868, 0.909]	0.903 [0.886, 0.92]	0.742 [0.712, 0.773]	0.077 [0.071, 0.084]	0.422 [0.405, 0.438]	0.79 [0.754, 0.823]	0.483 [0.425, 0.546]	1.0 [1.0, 1.0]	1.0 [1.0, 1.0]	0.893 [0.874, 0.911]	0.132 [0.132, 0.132]
Pain	0.976 [0.97, 0.982]	0.859 [0.835, 0.881]	0.887 [0.869, 0.904]	0.889 [0.871, 0.905]	0.876 [0.858, 0.894]	0.15 [0.146, 0.154]	0.683 [0.667, 0.698]	0.604 [0.587, 0.62]	0.772 [0.737, 0.804]	0.981 [0.97, 0.991]	0.969 [0.951, 0.985]	0.846 [0.822, 0.868]	0.161 [0.161, 0.161]
Dyspnoea	0.986 [0.982, 0.99]	0.884 [0.86, 0.907]	0.922 [0.907, 0.937]	0.923 [0.909, 0.937]	0.901 [0.882, 0.919]	0.089 [0.084, 0.093]	0.383 [0.375, 0.391]	0.587 [0.571, 0.603]	0.823 [0.787, 0.858]	0.979 [0.969, 0.988]	0.956 [0.935, 0.975]	0.909 [0.891, 0.928]	0.157 [0.157, 0.157]
Insomnia	0.987 [0.982, 0.991]	0.837 [0.801, 0.87]	0.924 [0.908, 0.941]	0.928 [0.915, 0.944]	0.863 [0.837, 0.887]	0.082 [0.076, 0.088]	0.393 [0.382, 0.403]	0.696 [0.673, 0.717]	0.73 [0.678, 0.777]	0.996 [0.991, 0.999]	0.984 [0.965, 0.996]	0.915 [0.898, 0.934]	0.145 [0.145, 0.145]
Appetite loss	0.988 [0.982, 0.993]	0.241 [0.098, 0.386]	0.955 [0.939, 0.968]	0.967 [0.957, 0.976]	0.57 [0.526, 0.62]	0.022 [0.017, 0.027]	0.218 [0.18, 0.254]	0.726 [0.599, 0.843]	0.139 [0.052, 0.239]	1.0 [1.0, 1.0]	1.0 [1.0, 1.0]	0.967 [0.956, 0.976]	0.045 [0.045, 0.045]
Constipation	0.993 [0.989, 0.996]	0.625 [0.515, 0.724]	0.962 [0.949, 0.974]	0.968 [0.957, 0.978]	0.731 [0.674, 0.787]	0.021 [0.017, 0.026]	0.21 [0.188, 0.23]	0.651 [0.583, 0.715]	0.462 [0.349, 0.575]	0.999 [0.998, 1.0]	0.973 [0.912, 1.0]	0.968 [0.958, 0.977]	0.057 [0.057, 0.057]
Diarrhoea	0.975 [0.968, 0.981]	0.0 [0.0, 0.0]	0.757 [0.729, 0.784]	0.833 [0.813, 0.852]	0.5 [0.5, 0.5]	0.1 [0.091, 0.109]	0.693 [0.66, 0.725]	1.253 [1.192, 1.315]	0.0 [0.0, 0.0]	1.0 [1.0, 1.0]	nan [nan, nan]	0.833 [0.813, 0.852]	0.109 [0.109, 0.109]
Financial difficulties	0.995 [0.993, 0.997]	0.898 [0.867, 0.927]	0.962 [0.951, 0.973]	0.963 [0.953, 0.973]	0.914 [0.89, 0.938]	0.04 [0.036, 0.044]	0.262 [0.254, 0.27]	0.6 [0.579, 0.621]	0.835 [0.785, 0.882]	0.994 [0.989, 0.998]	0.972 [0.948, 0.991]	0.962 [0.95, 0.973]	0.112 [0.112, 0.112]

Figure S1. Distribution of time difference between assessments.

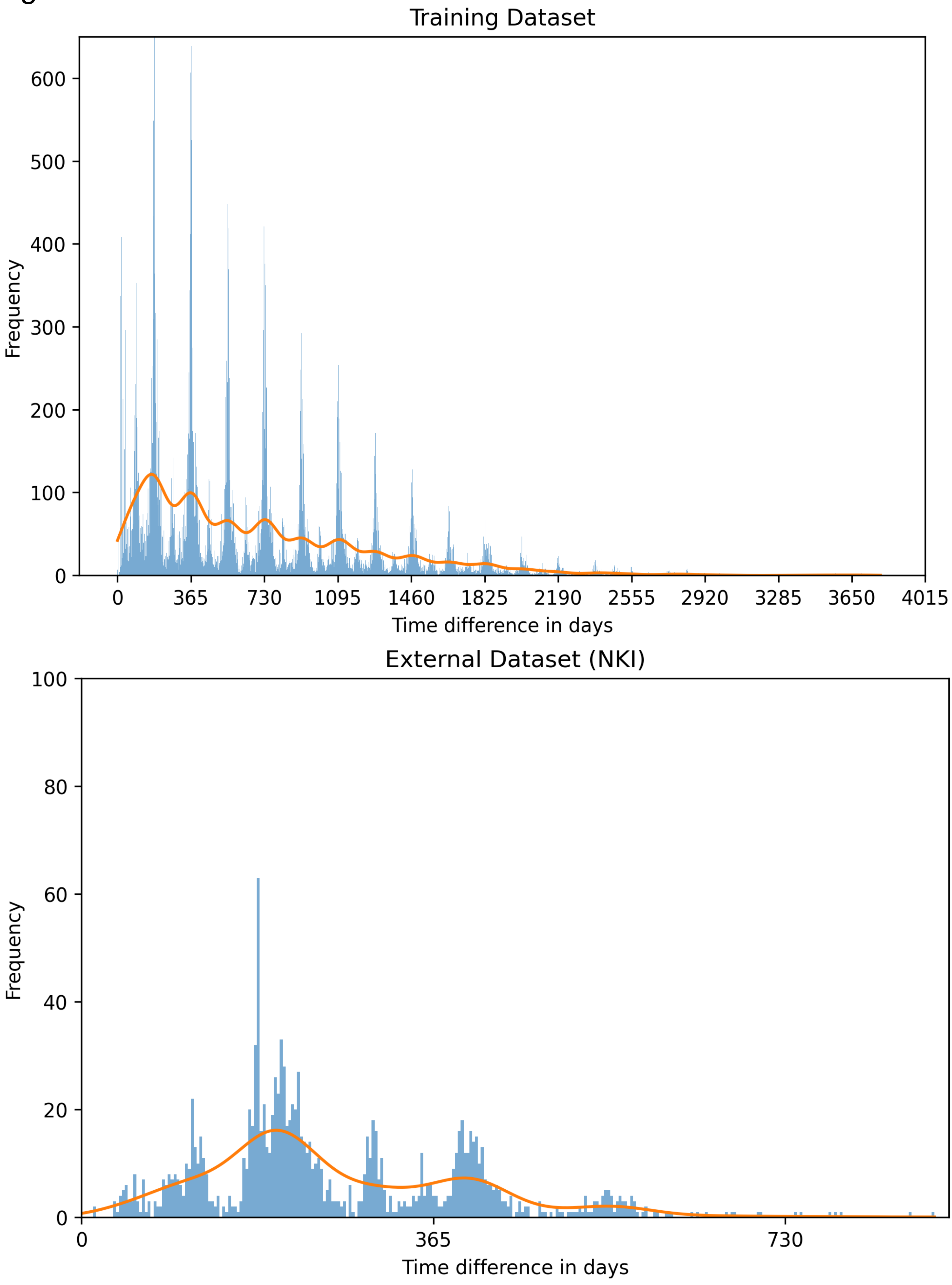

Figure S2. Calibration plots.

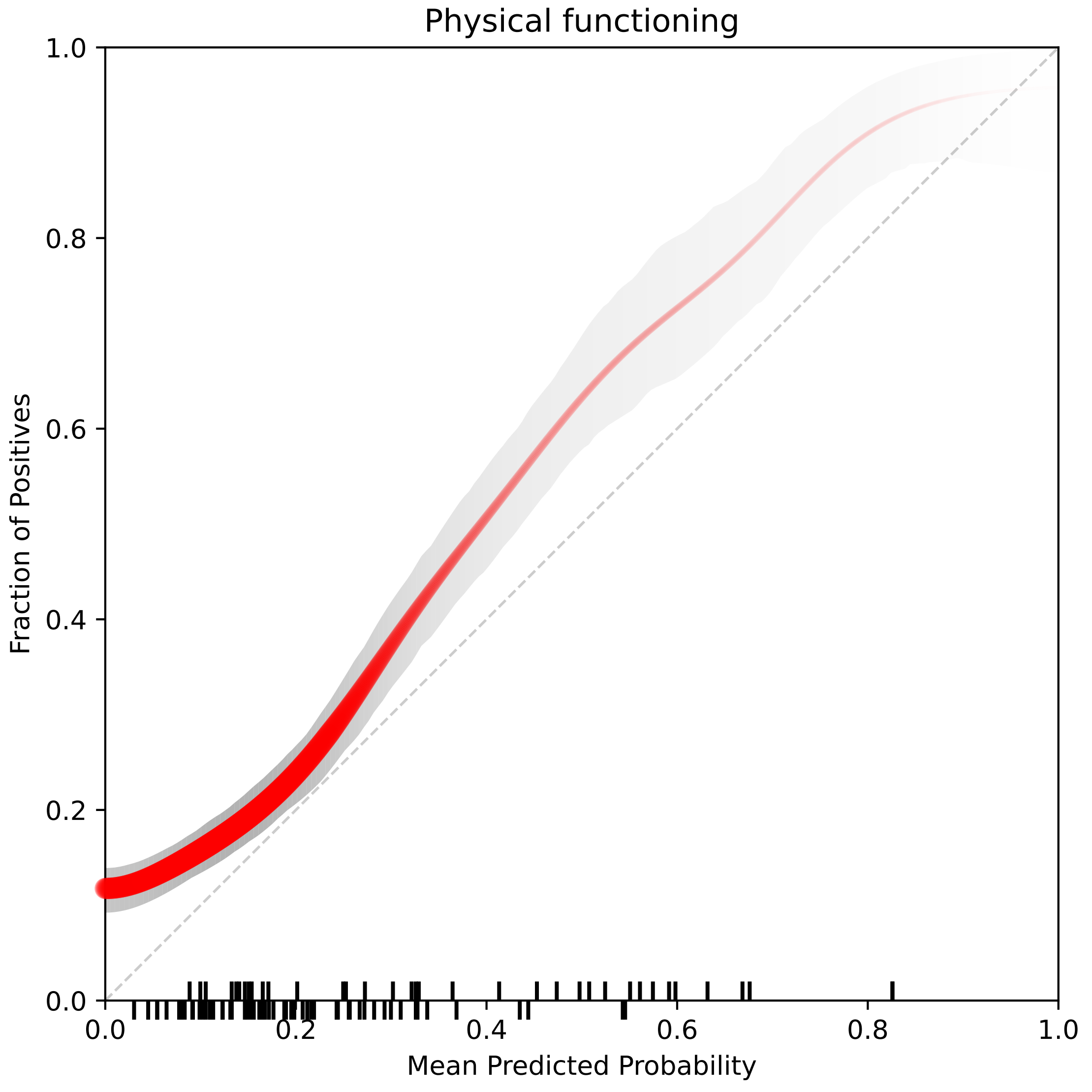

Physical functioning
Fraction of Positives
Mean Predicted Probability
0.0
0.2
0.4
0.6
0.8
1.0

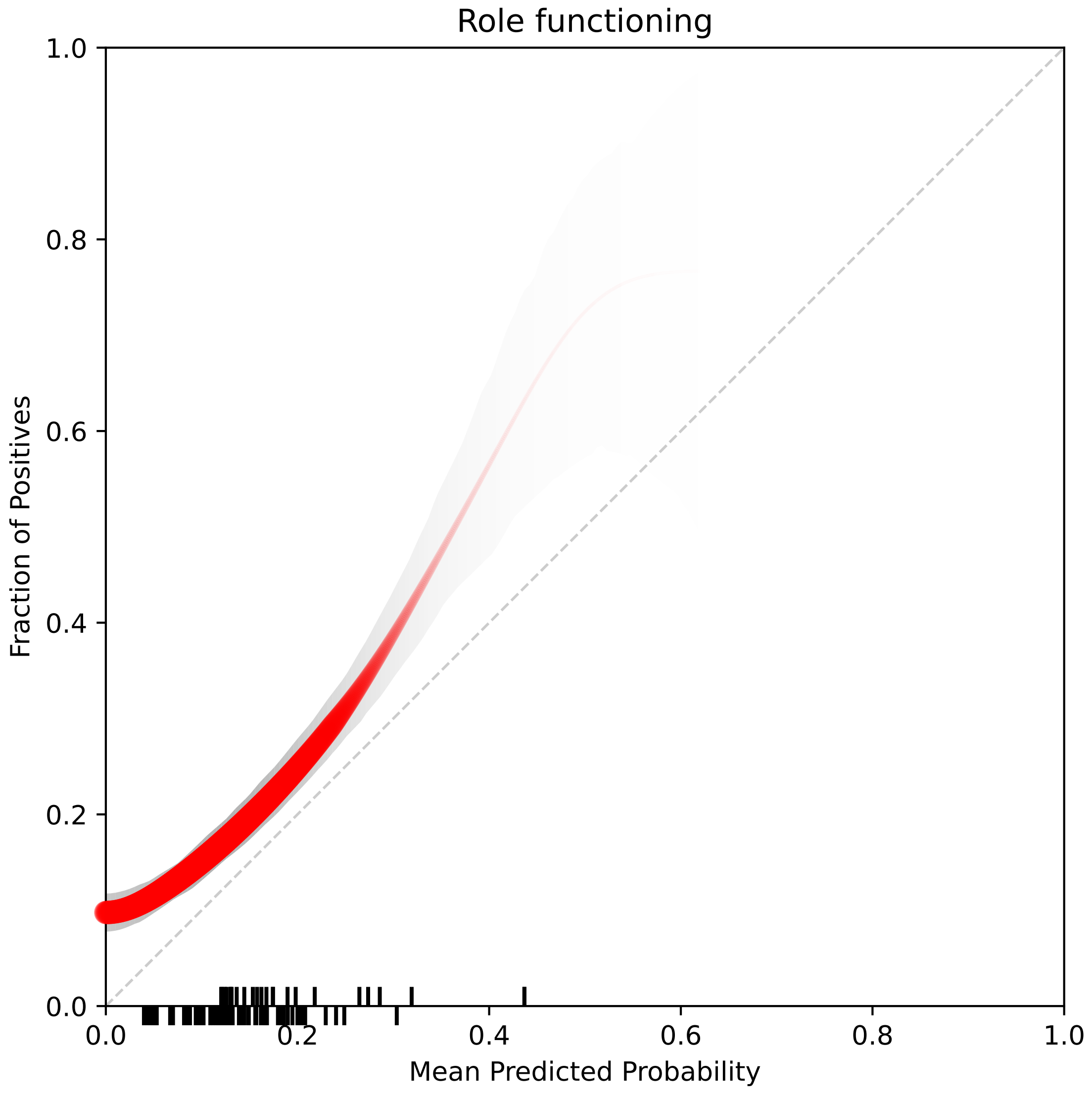

Role functioning
Fraction of Positives
Mean Predicted Probability
0.0
0.2
0.4
0.6
0.8
1.0

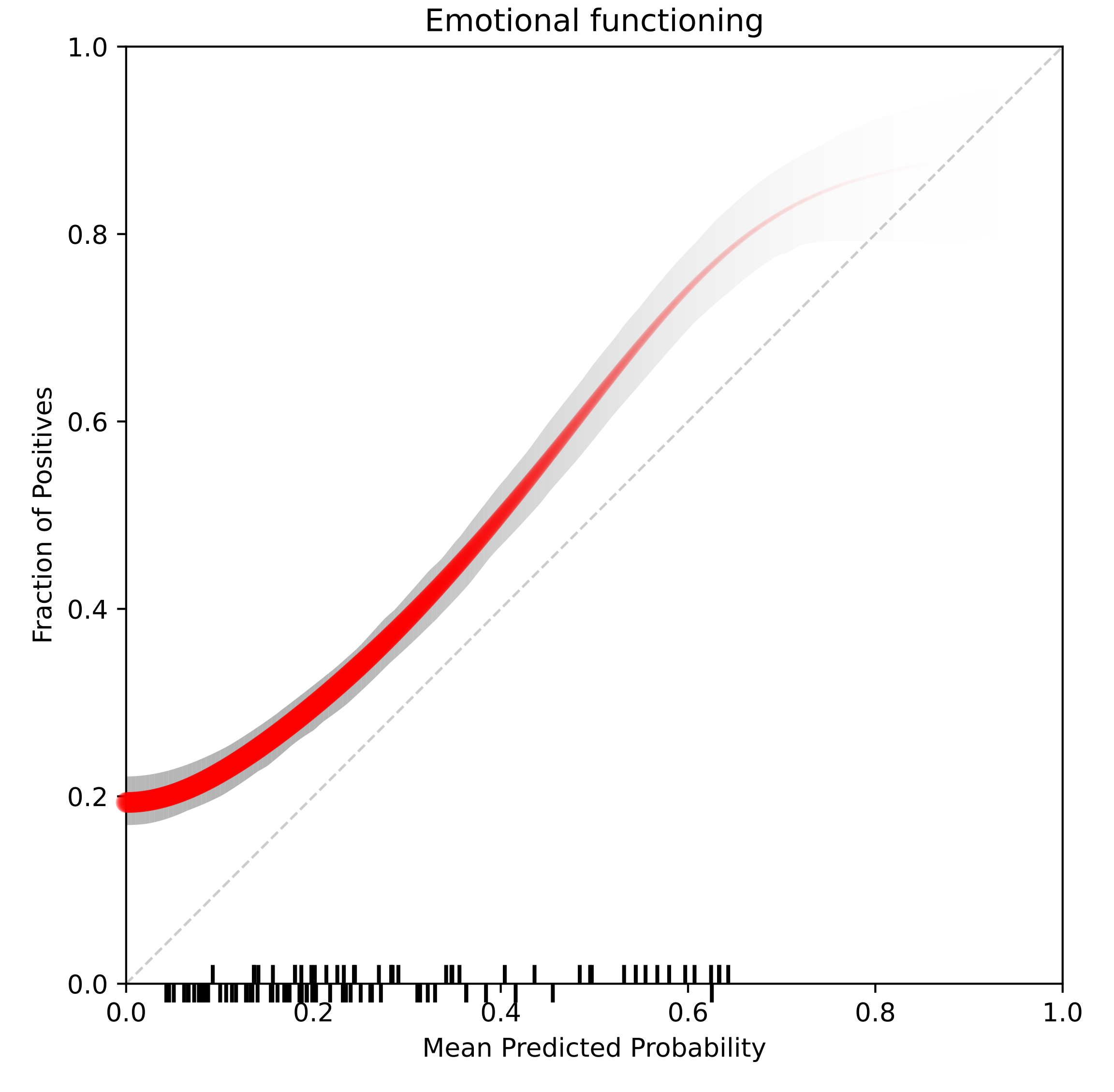

Emotional functioning
Fraction of Positives
Mean Predicted Probability
0.0
0.2
0.4
0.6
0.8
1.0
0.0
0.2
0.4
0.6
0.8
1.0

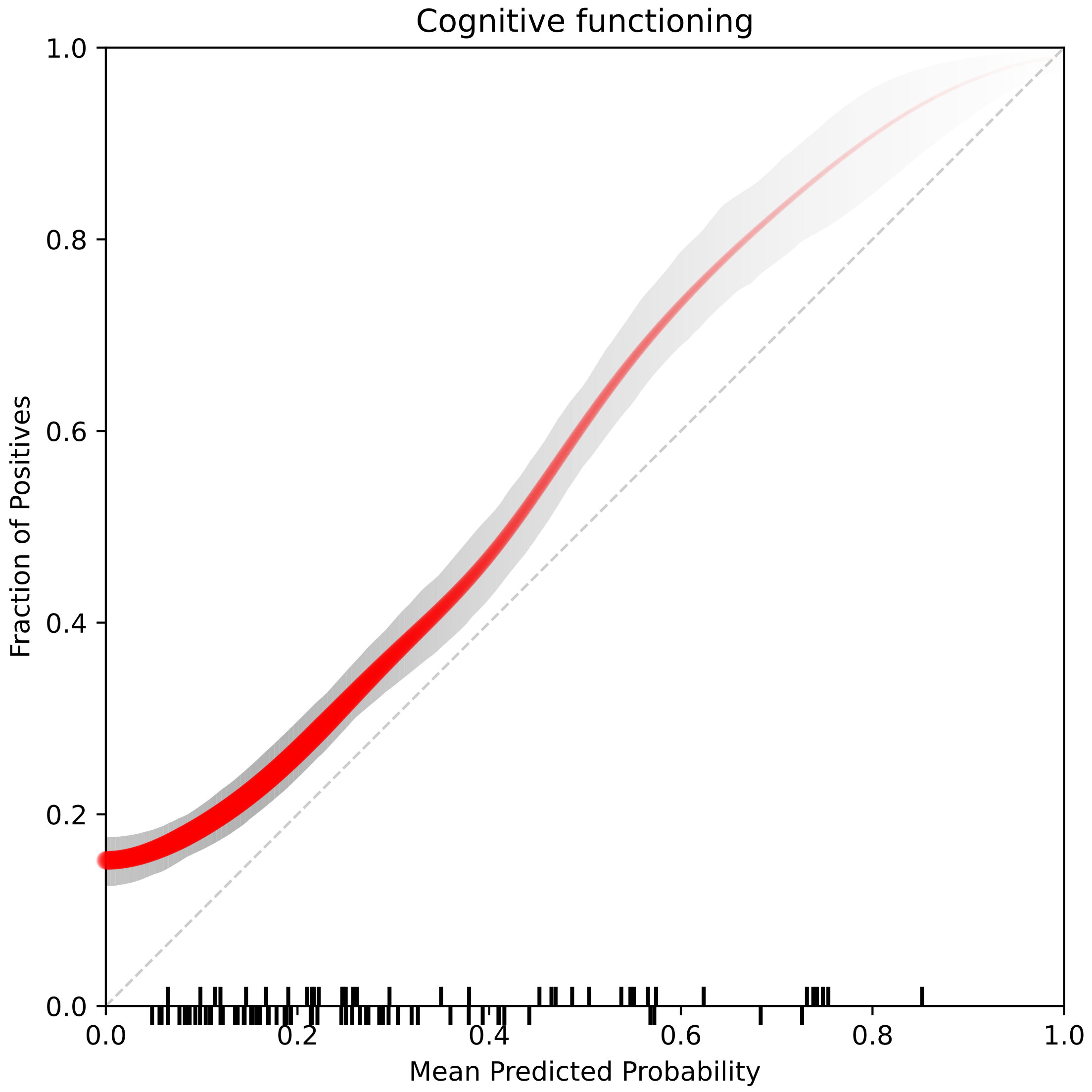
Cognitive functioning
Fraction of Positives
Mean Predicted Probability
0.0
0.2
0.4
0.6
0.8
1.0

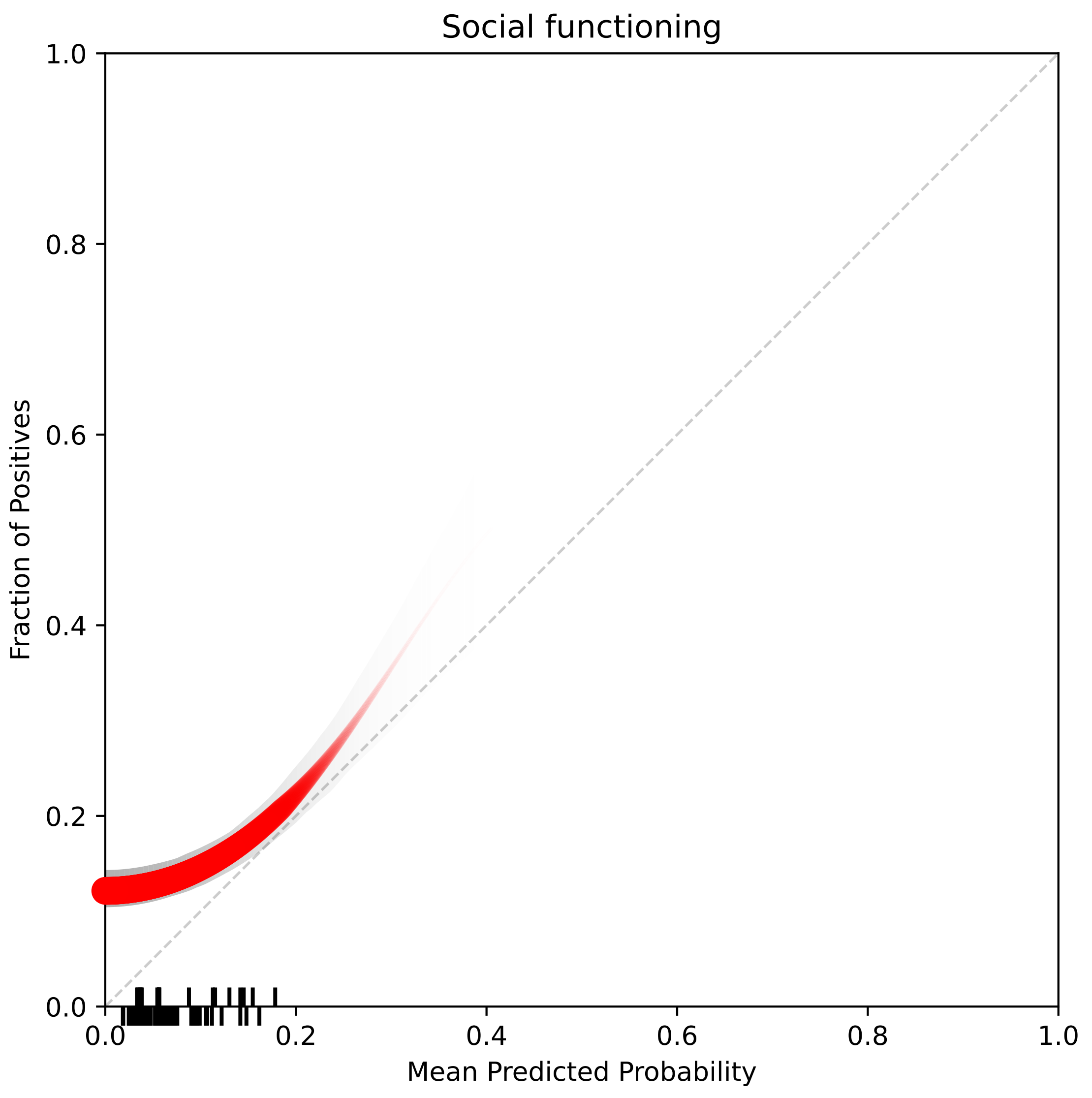
Social functioning
Fraction of Positives
Mean Predicted Probability
0.0
0.2
0.4
0.6
0.8
1.0

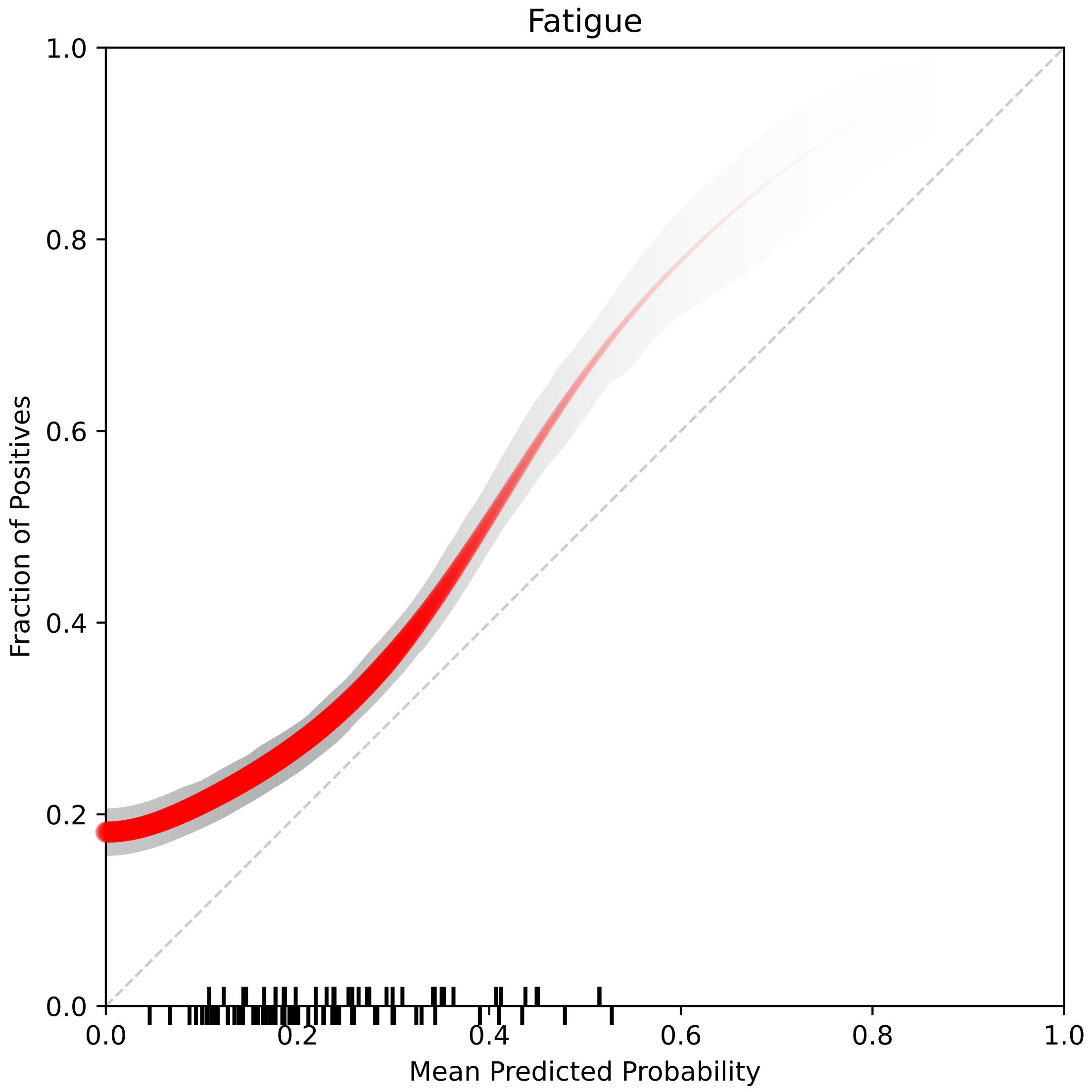

Fatigue
Fraction of Positives
Mean Predicted Probability
0.0
0.2
0.4
0.6
0.8
1.0

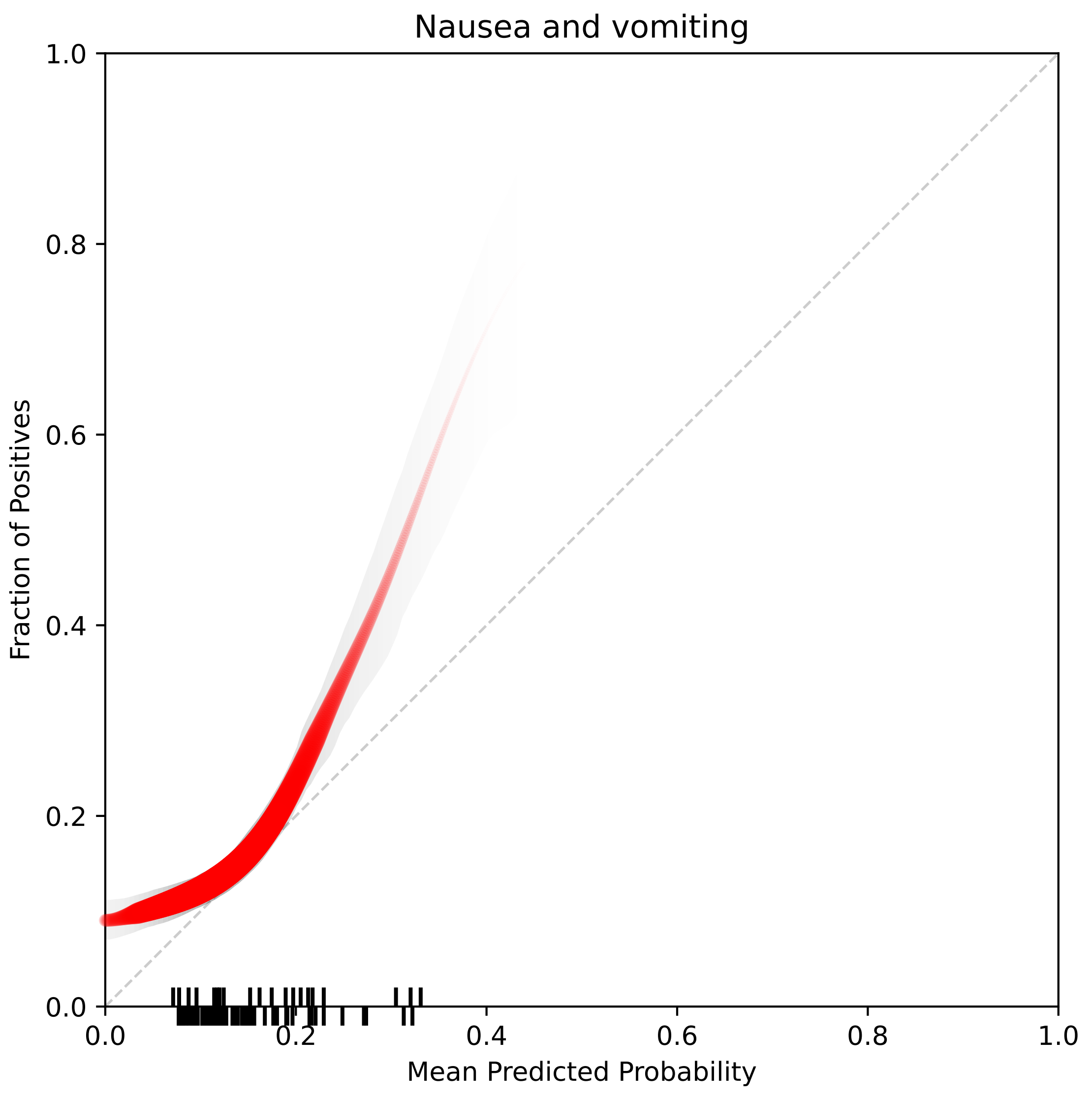

Nausea and vomiting
Fraction of Positives
Mean Predicted Probability
0.0
0.2
0.4
0.6
0.8
1.0

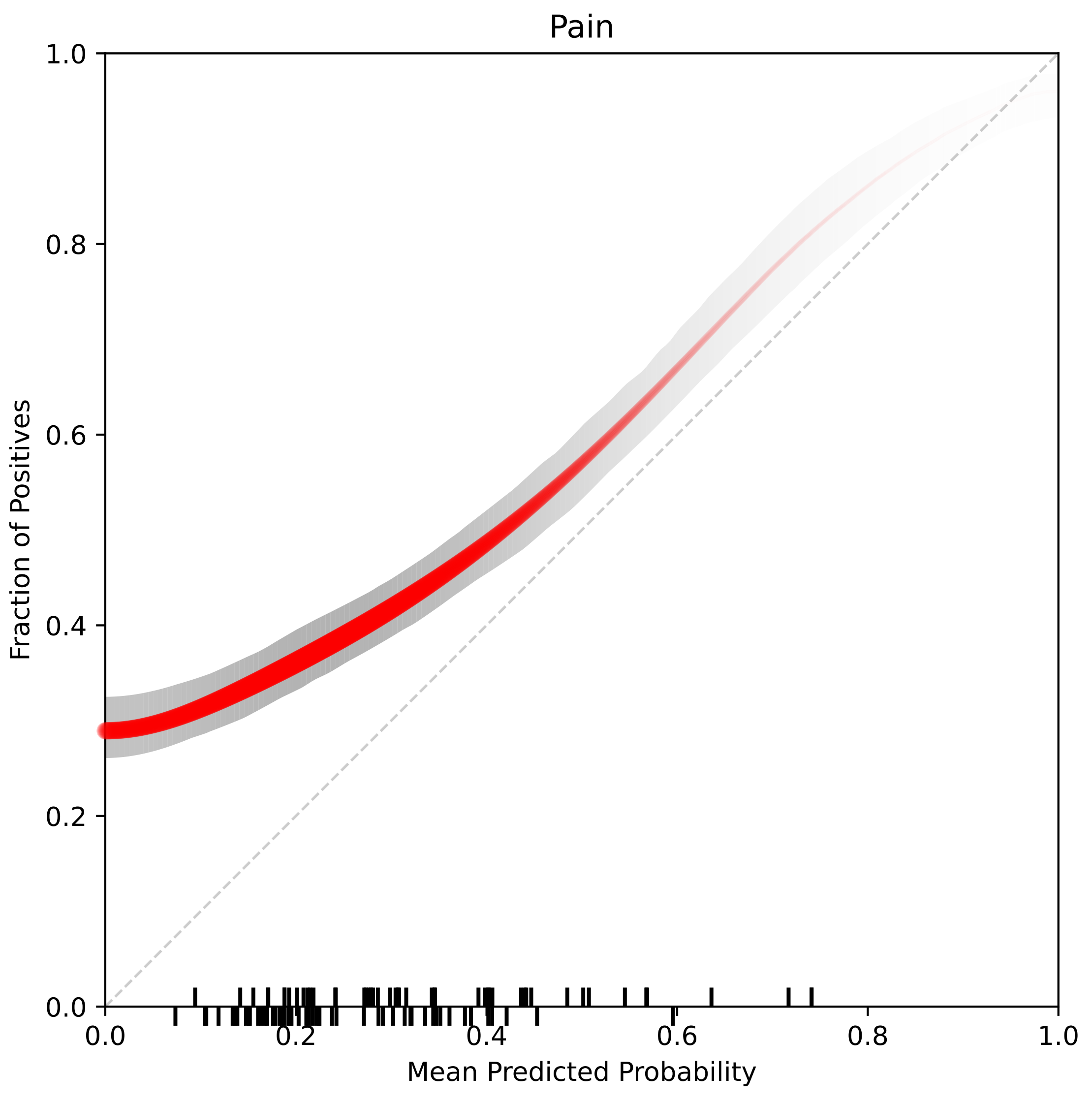

Pain
Fraction of Positives
Mean Predicted Probability
0.0
0.2
0.4
0.6
0.8
1.0

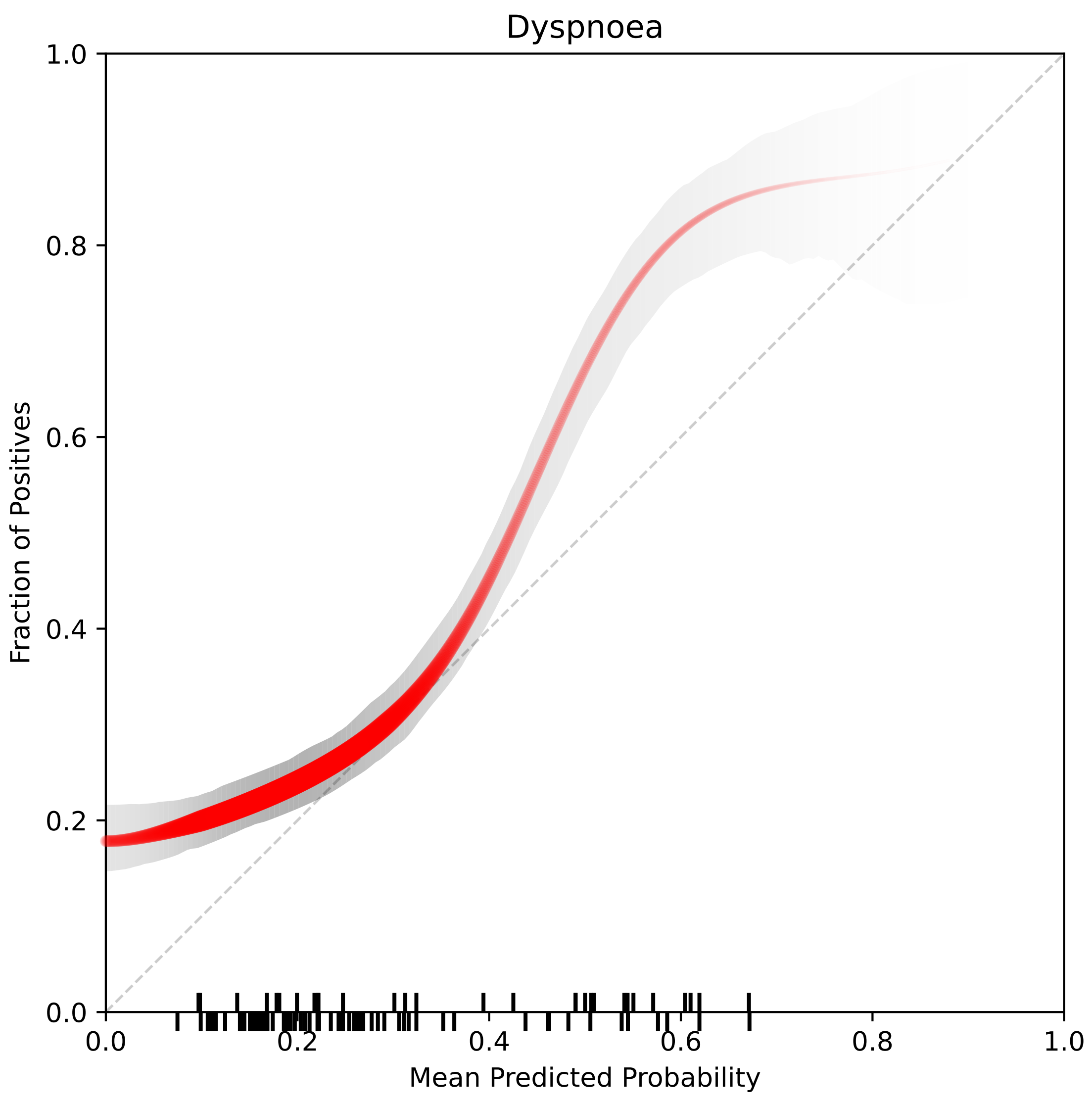

Dyspnoea
Fraction of Positives
Mean Predicted Probability
0.0
0.2
0.4
0.6
0.8
1.0

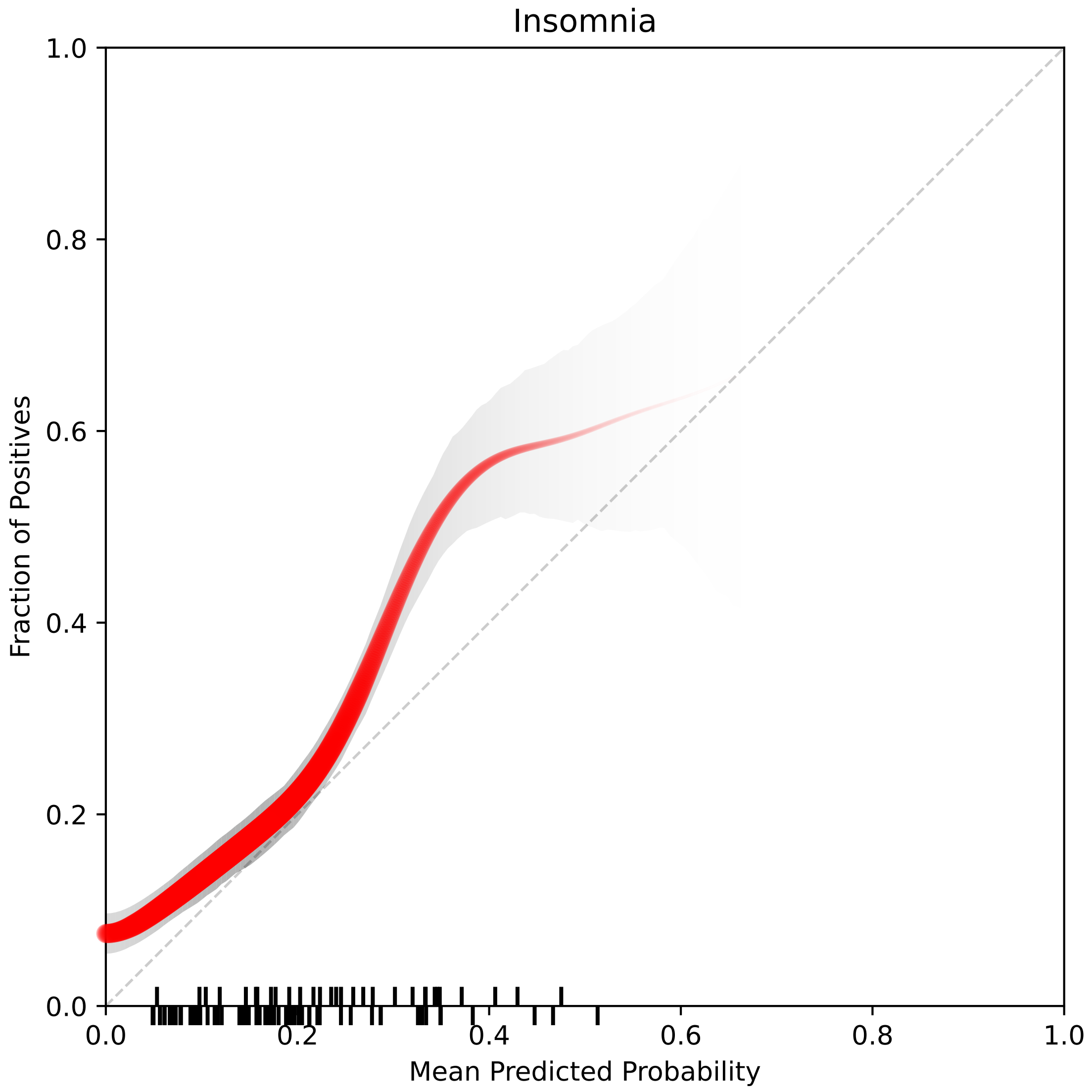

Insomnia
1.0
0.8
0.6
0.4
0.2
0.0
0.0
0.2
0.4
0.6
0.8
1.0
Fraction of Positives
Mean Predicted Probability

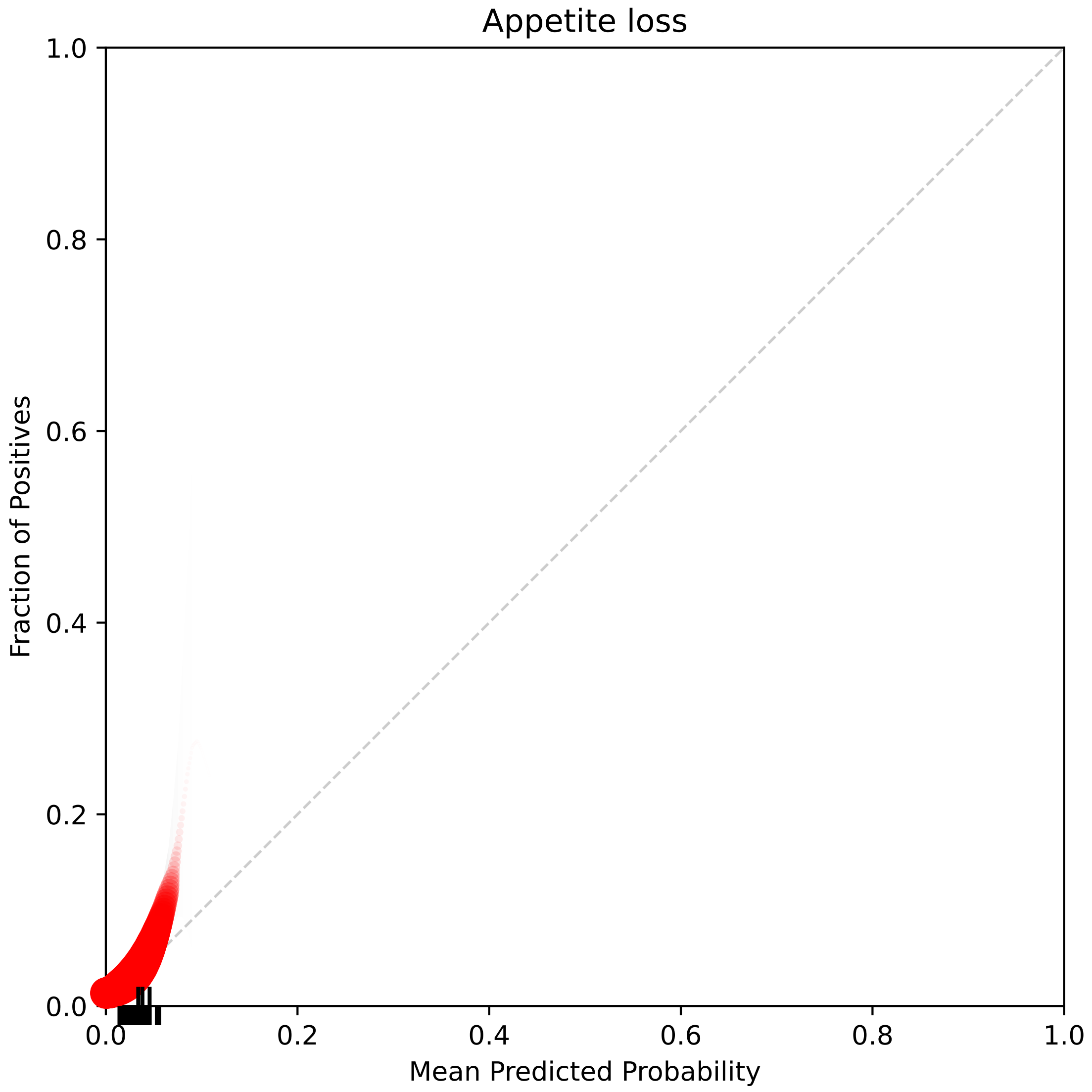

Appetite loss
Fraction of Positives
Mean Predicted Probability
0.0
0.2
0.4
0.6
0.8
1.0

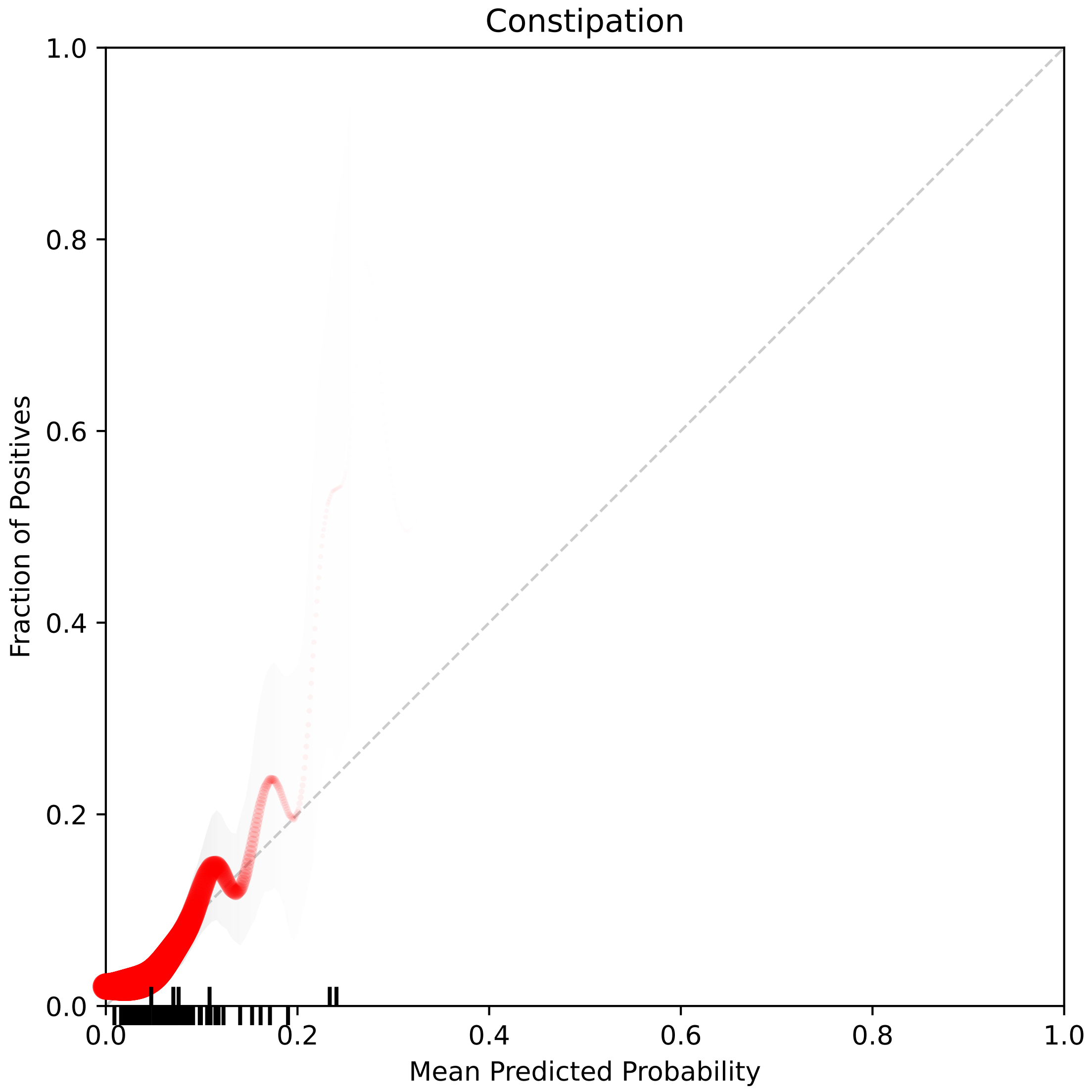

Constipation
Fraction of Positives
Mean Predicted Probability
0.0
0.2
0.4
0.6
0.8
1.0

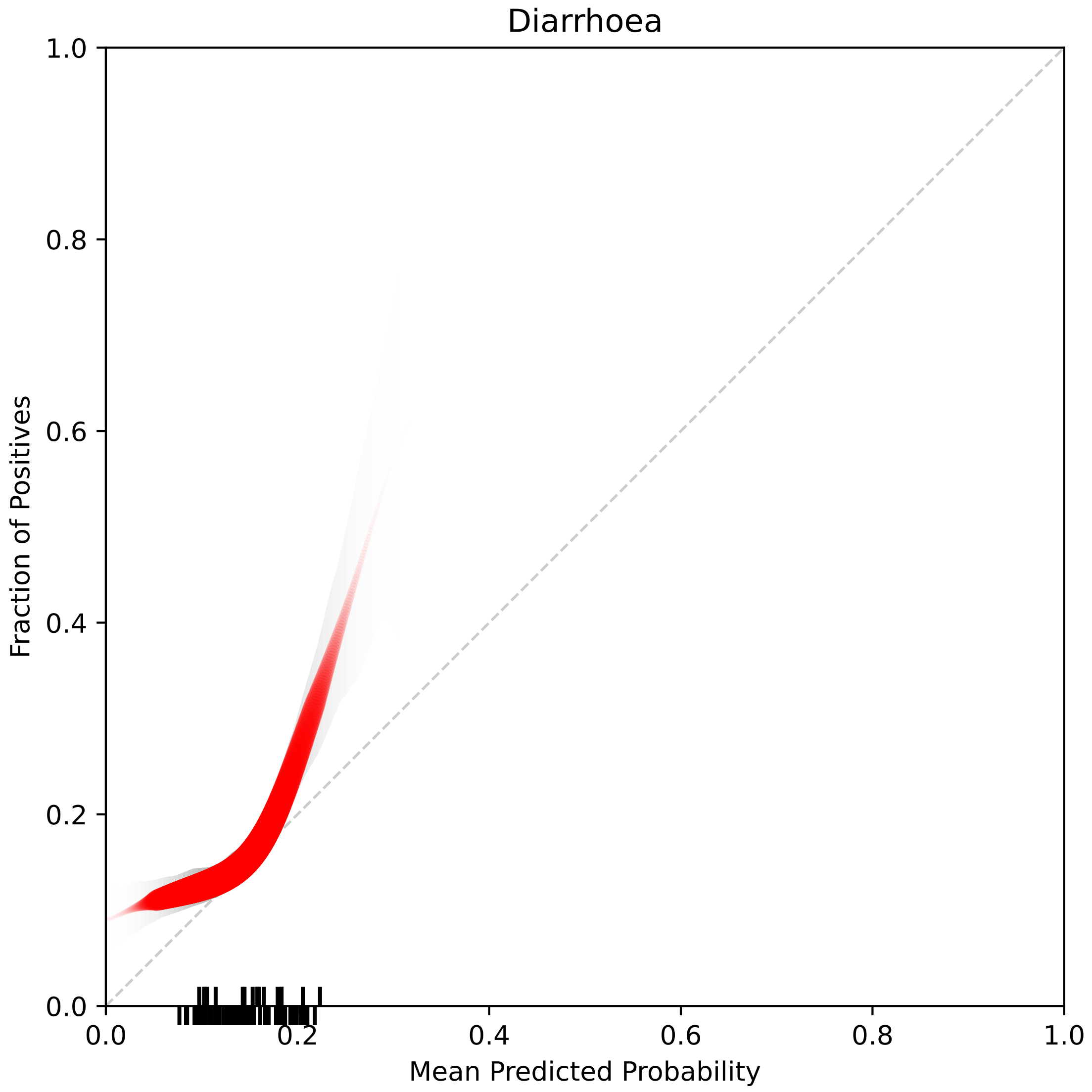

Diarrhoea
Fraction of Positives
Mean Predicted Probability
0.0
0.2
0.4
0.6
0.8
1.0

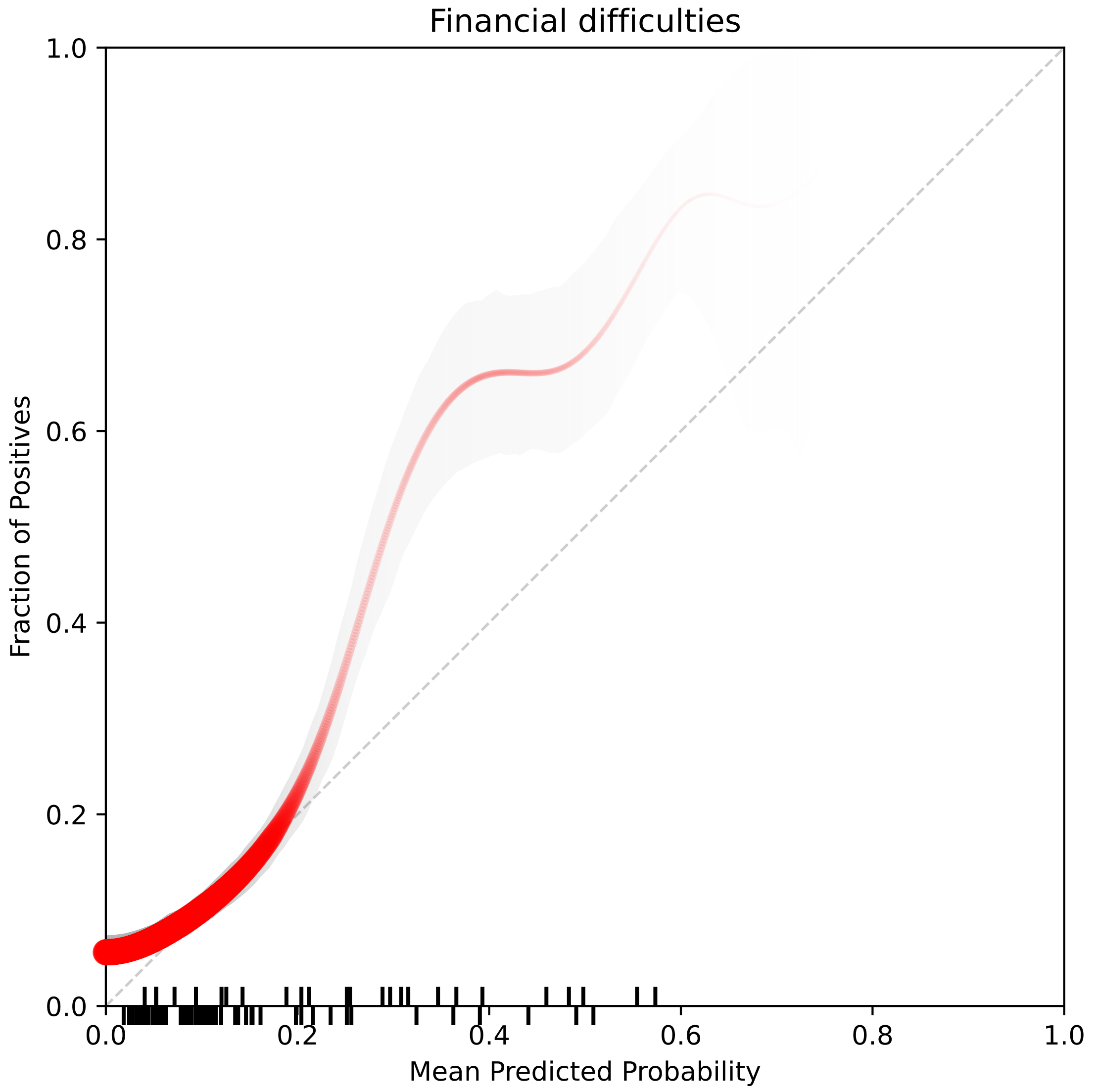

Financial difficulties
Fraction of Positives
Mean Predicted Probability
0.0
0.2
0.4
0.6
0.8
1.0

Figure S3. Post hoc recalibration plots.

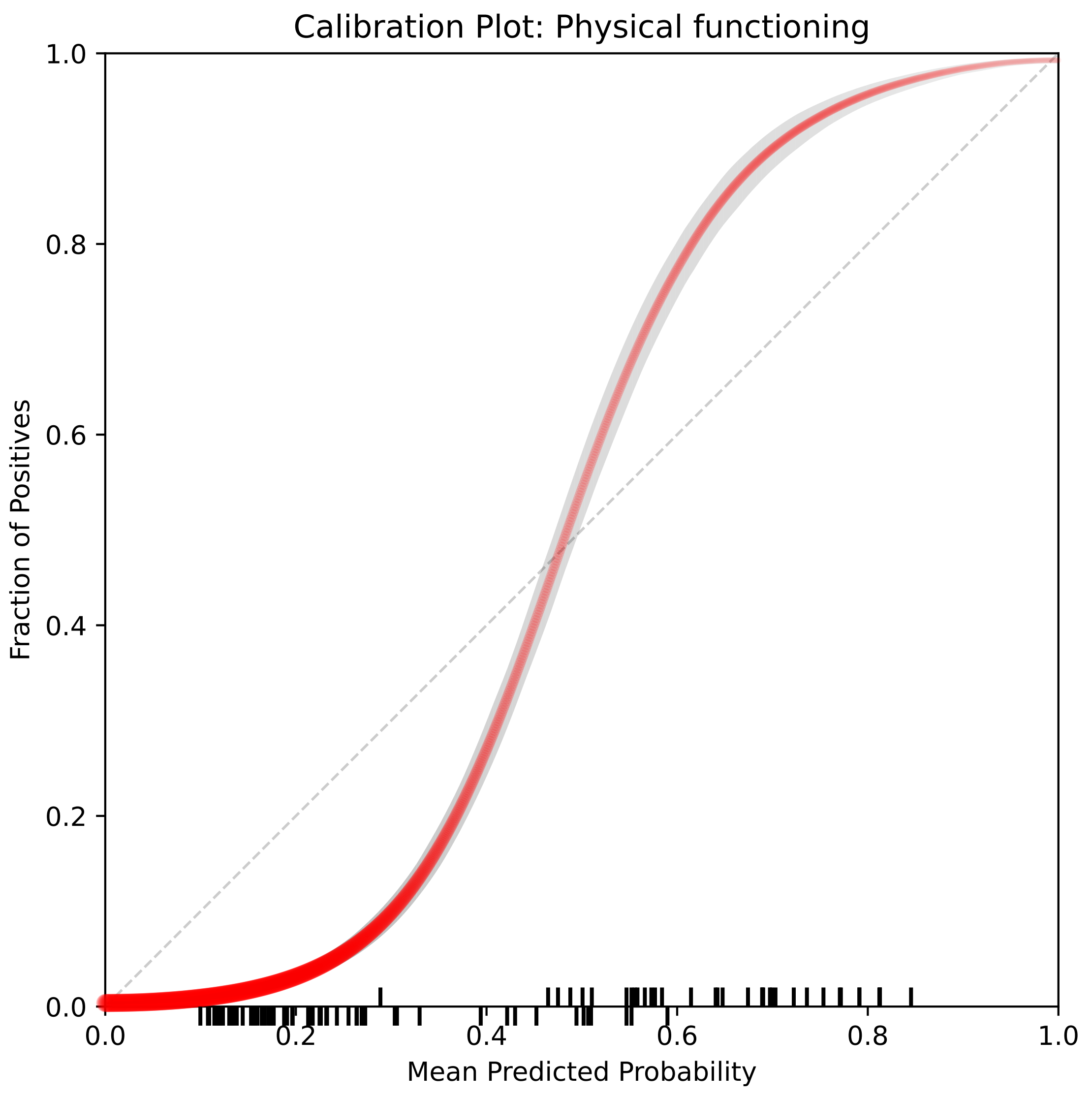

Calibration Plot: Physical functioning
Fraction of Positives
Mean Predicted Probability
0.0
0.2
0.4
0.6
0.8
1.0

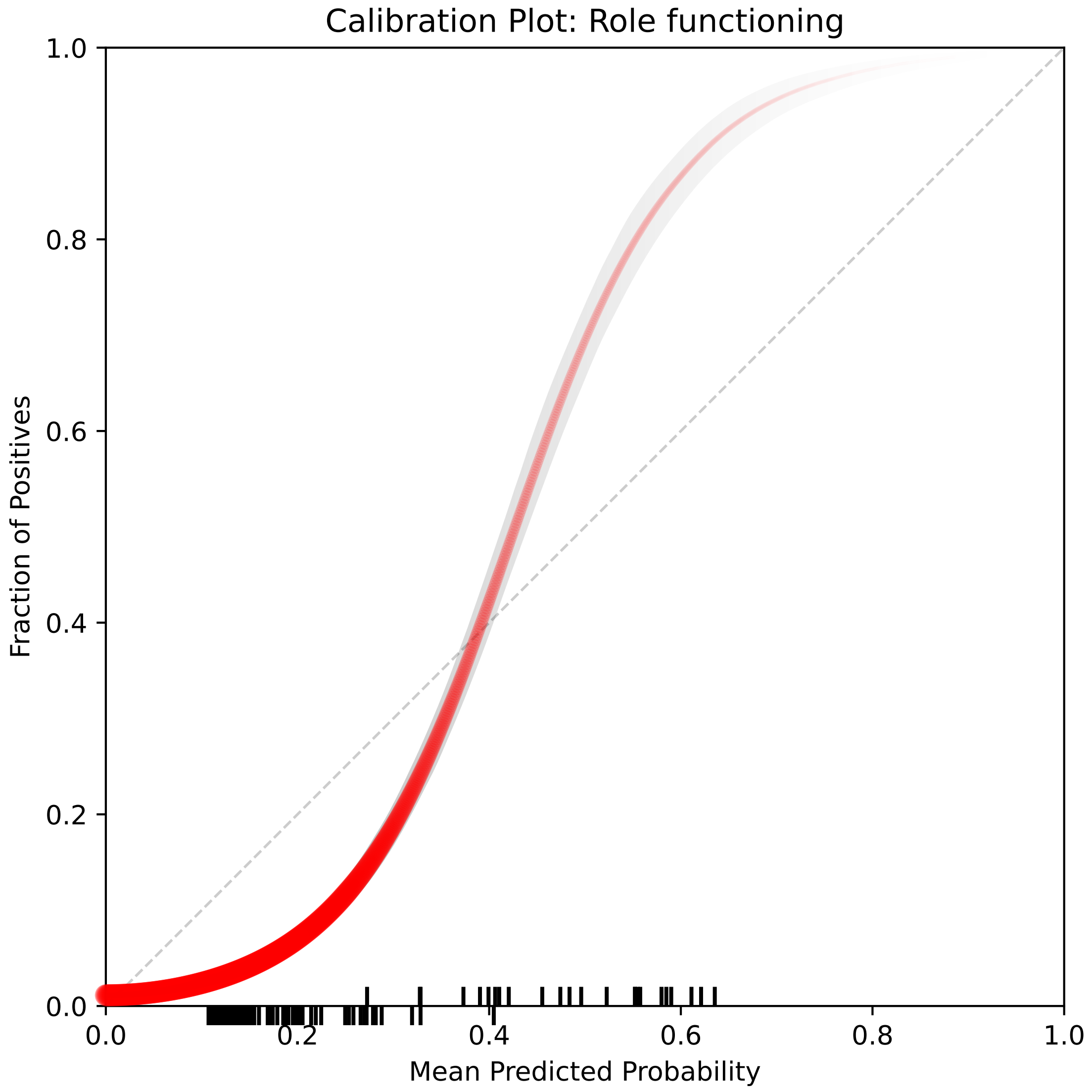

Calibration Plot: Role functioning
Fraction of Positives
Mean Predicted Probability
0.0
0.2
0.4
0.6
0.8
1.0

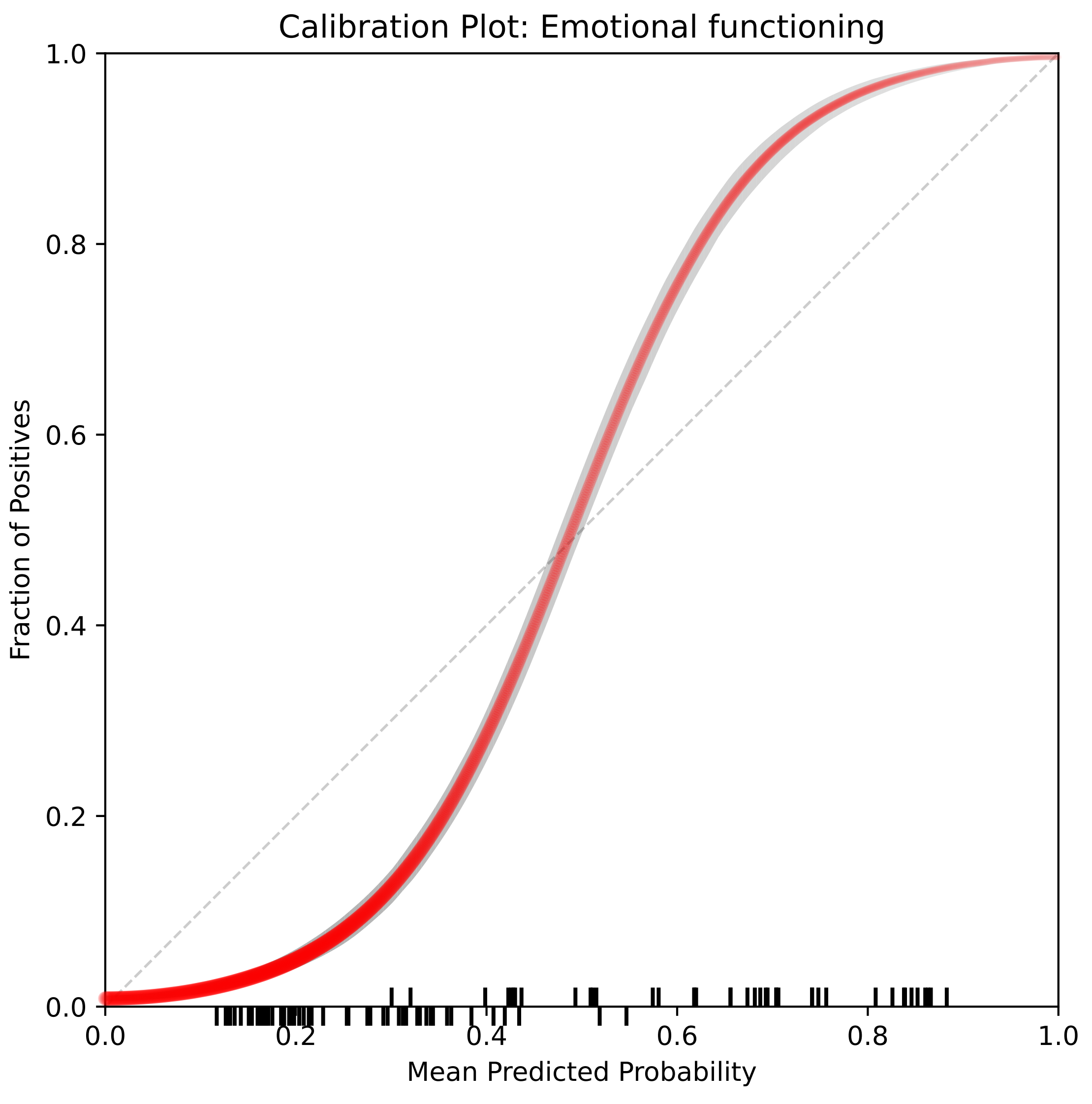

Calibration Plot: Emotional functioning
Fraction of Positives
Mean Predicted Probability
0.0
0.2
0.4
0.6
0.8
1.0

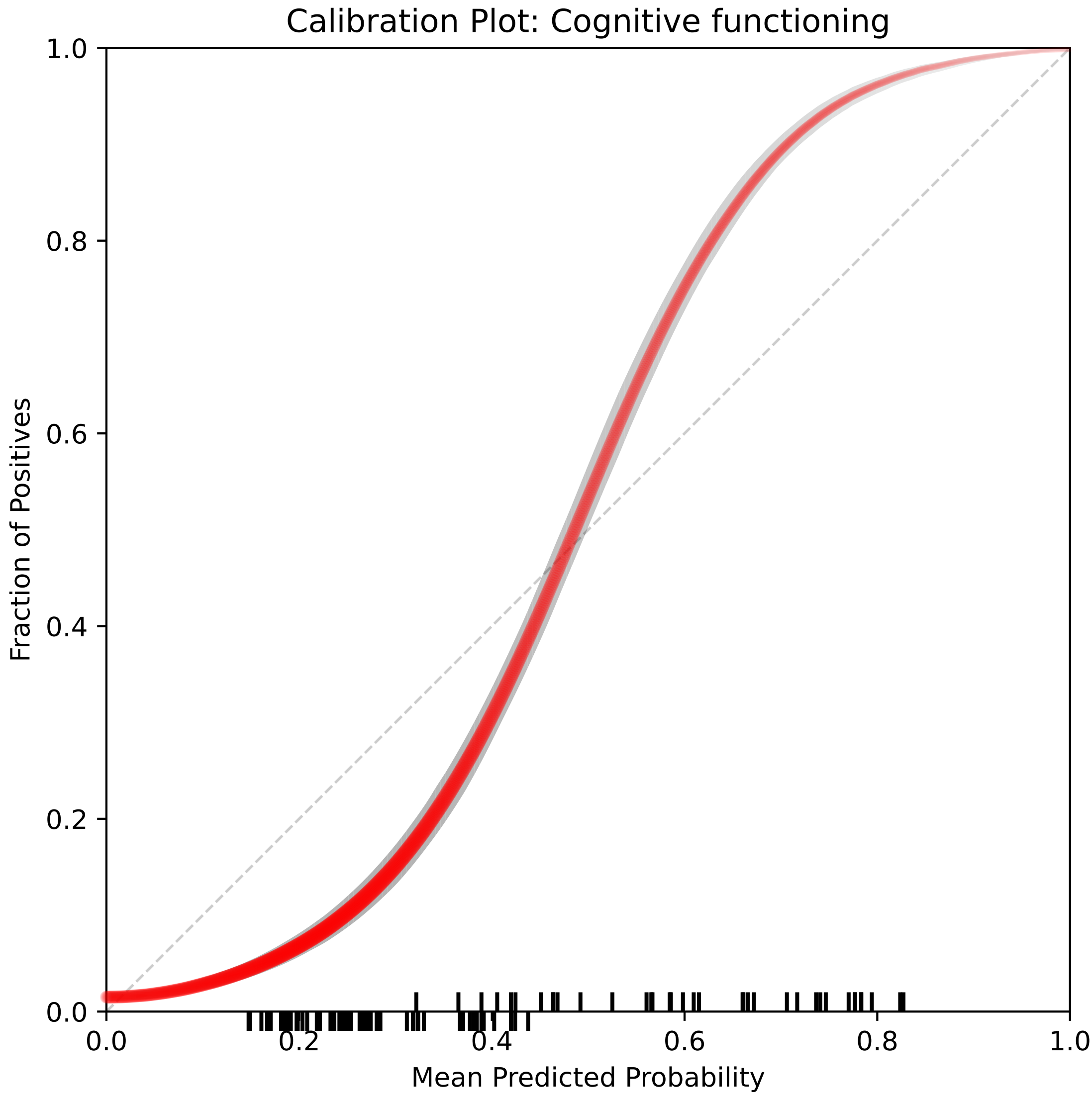

Calibration Plot: Cognitive functioning
Fraction of Positives
Mean Predicted Probability
0.0
0.2
0.4
0.6
0.8
1.0

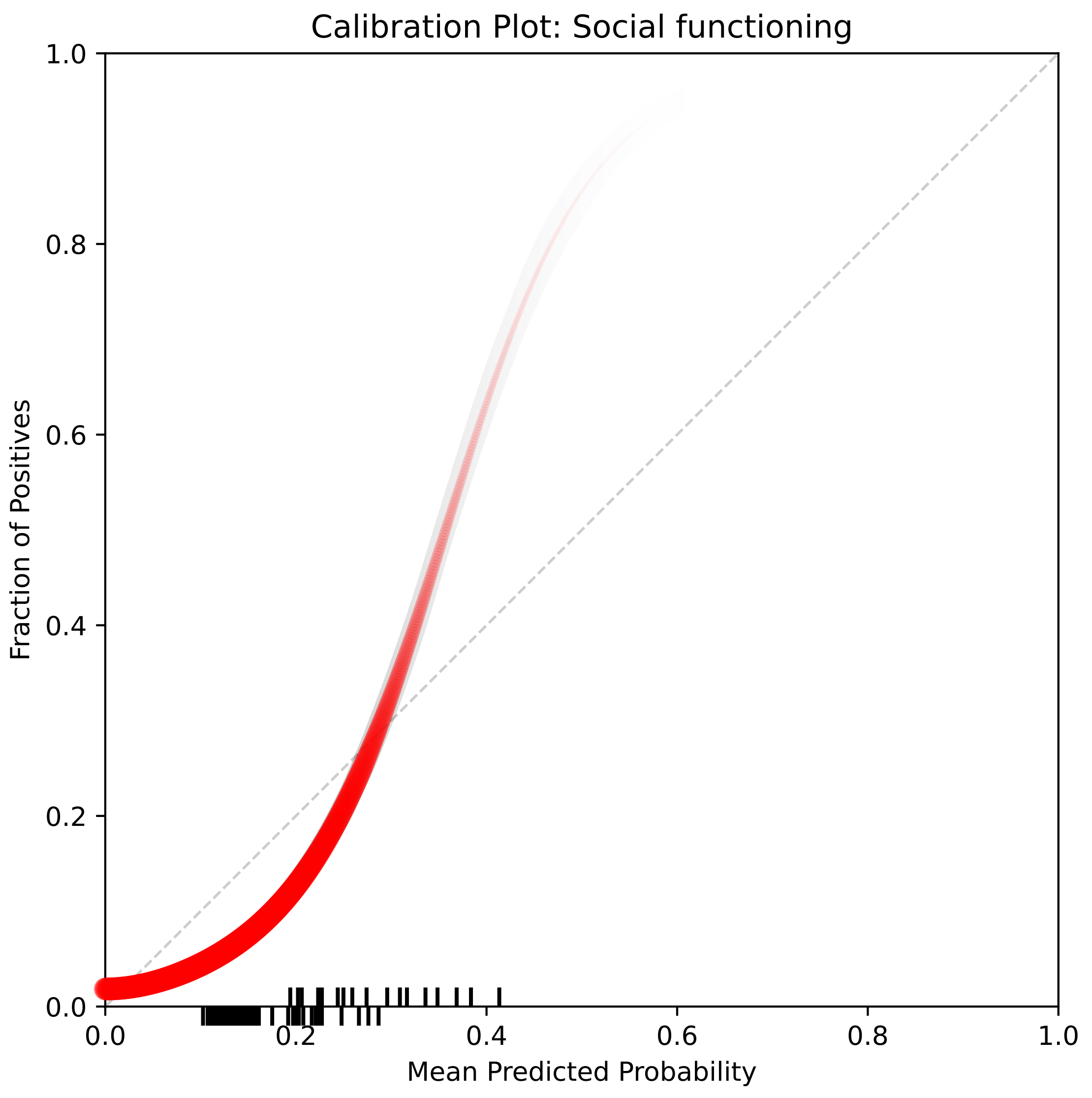

Calibration Plot: Social functioning
Fraction of Positives
Mean Predicted Probability
0.0
0.2
0.4
0.6
0.8
1.0

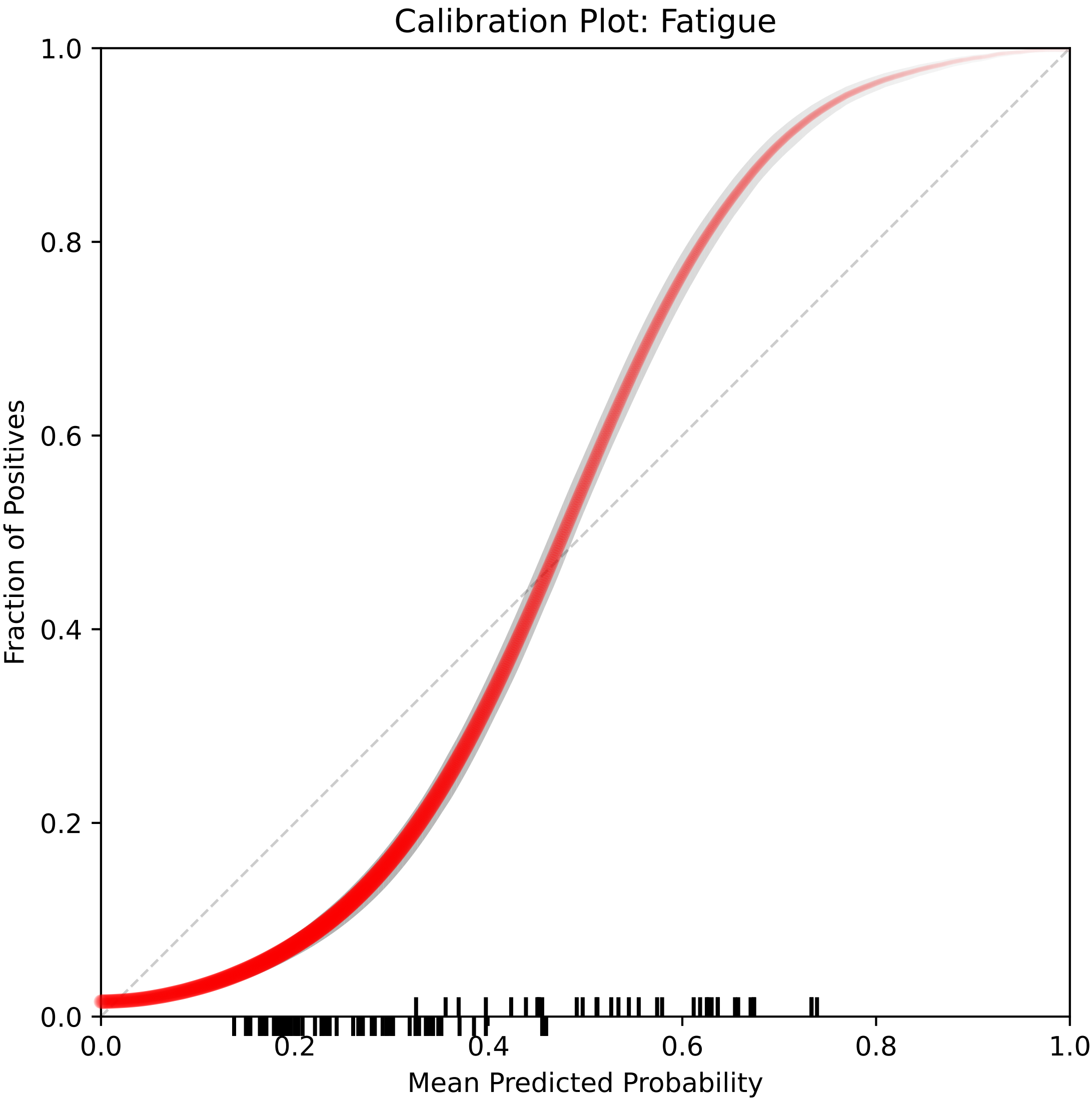

Calibration Plot: Fatigue
Fraction of Positives
Mean Predicted Probability
0.0
0.2
0.4
0.6
0.8
1.0

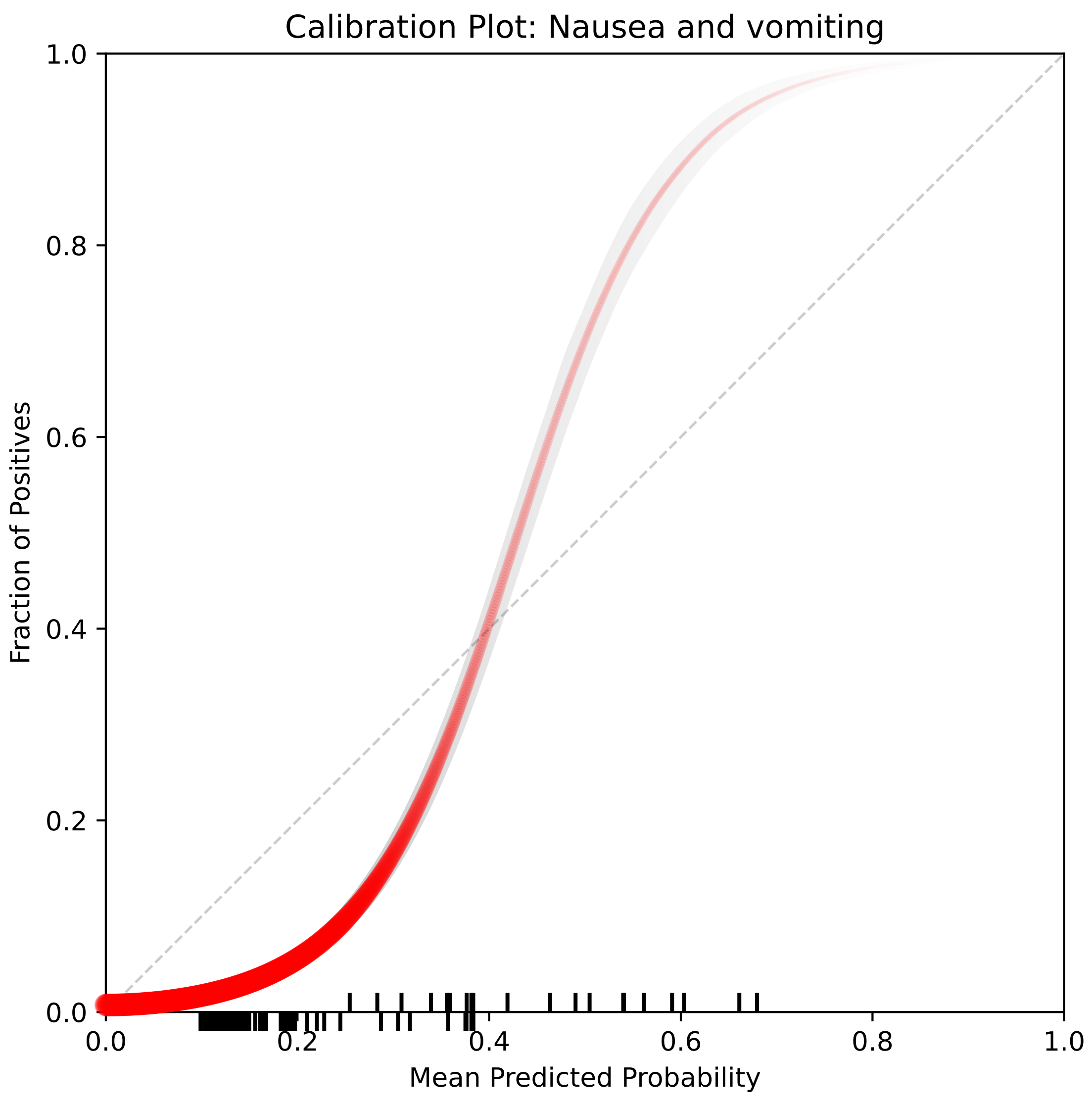

Calibration Plot: Nausea and vomiting
Fraction of Positives
Mean Predicted Probability
0.0
0.2
0.4
0.6
0.8
1.0

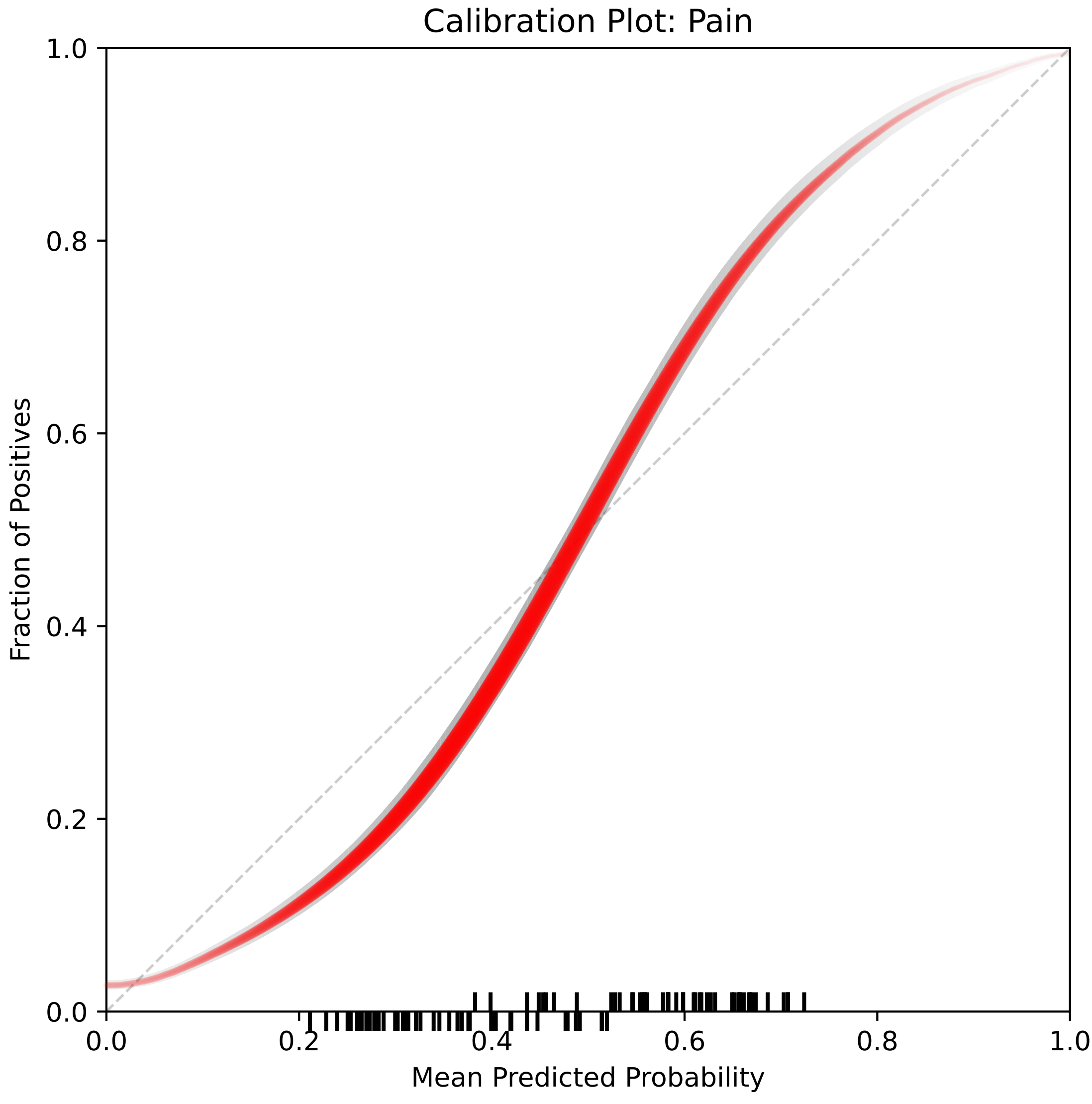
Calibration Plot: Pain
Fraction of Positives
Mean Predicted Probability
0.0
0.2
0.4
0.6
0.8
1.0
0.0
0.2
0.4
0.6
0.8
1.0

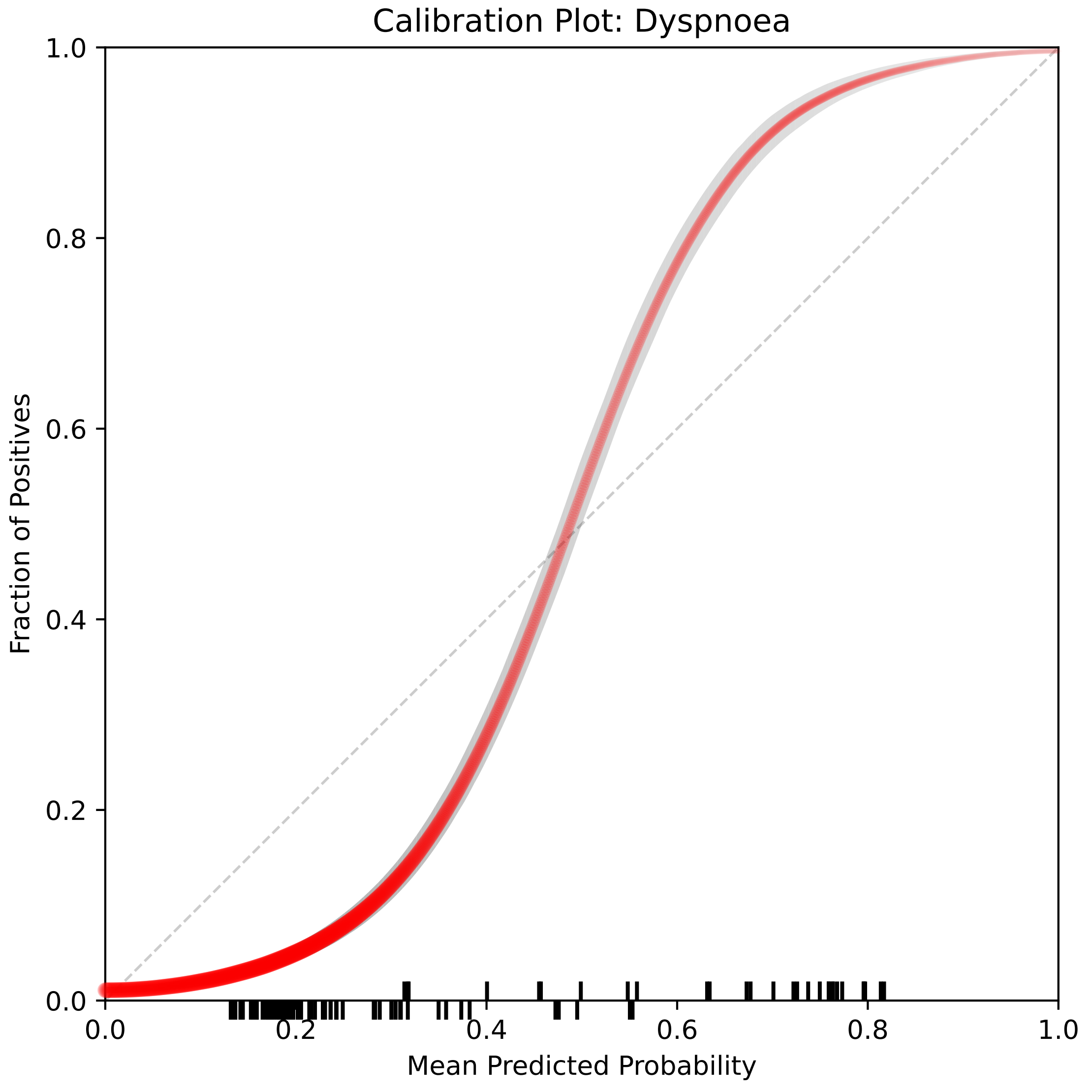

Calibration Plot: Dyspnoea
Fraction of Positives
Mean Predicted Probability
0.0
0.2
0.4
0.6
0.8
1.0

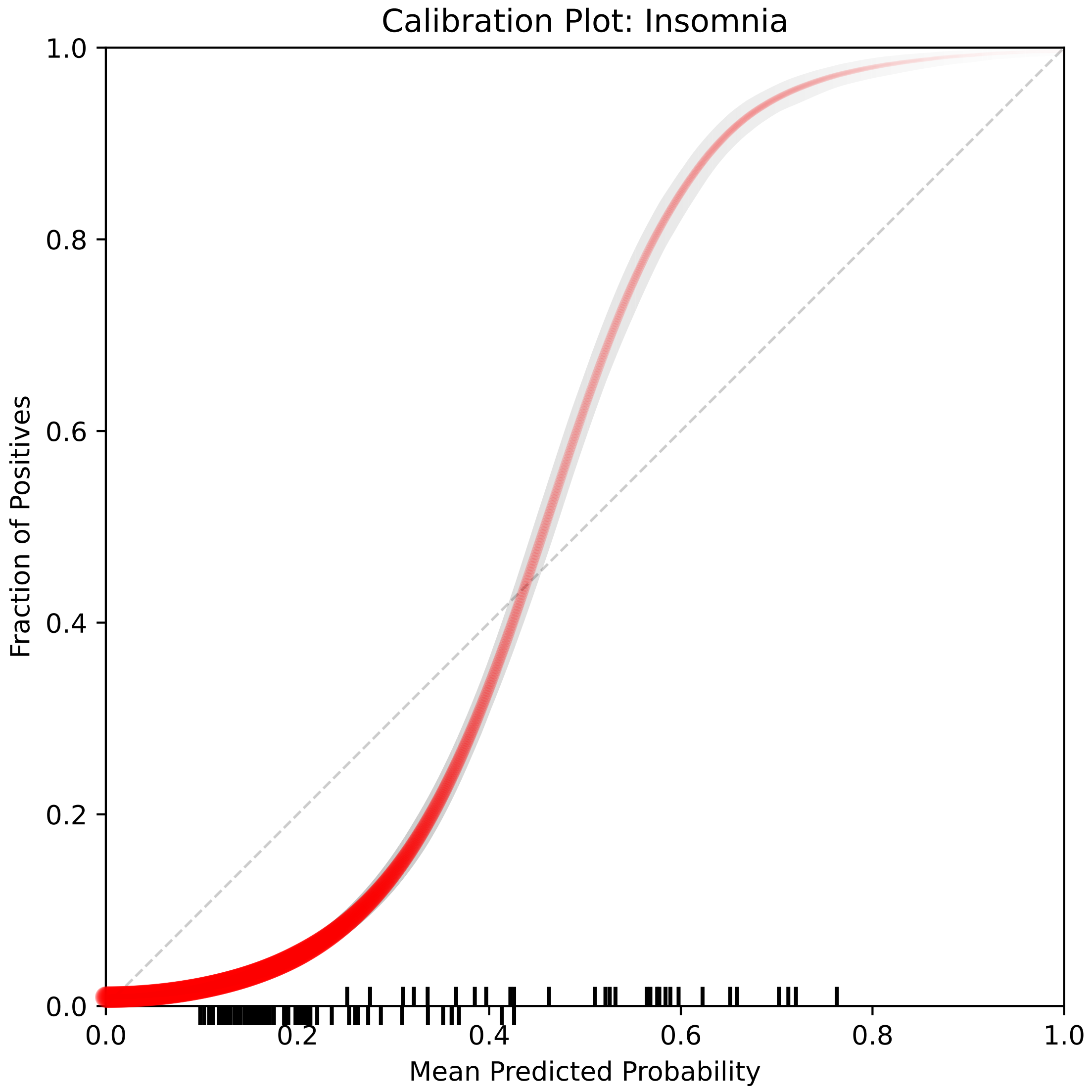

Calibration Plot: Insomnia
1.0
0.8
0.6
0.4
0.2
0.0
Fraction of Positives
0.0
0.2
0.4
0.6
0.8
1.0
Mean Predicted Probability

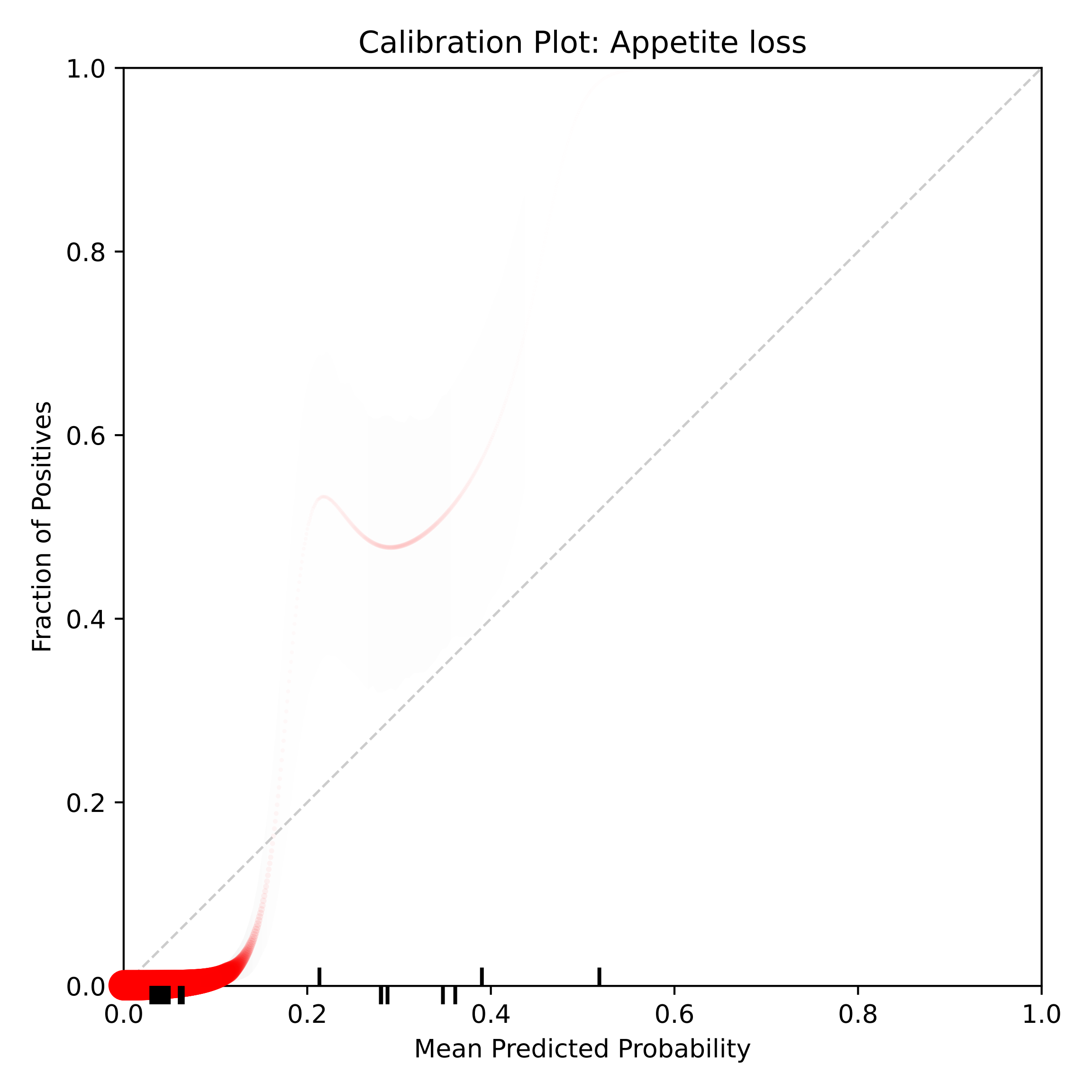
Calibration Plot: Appetite loss
Fraction of Positives
Mean Predicted Probability
0.0
0.2
0.4
0.6
0.8
1.0

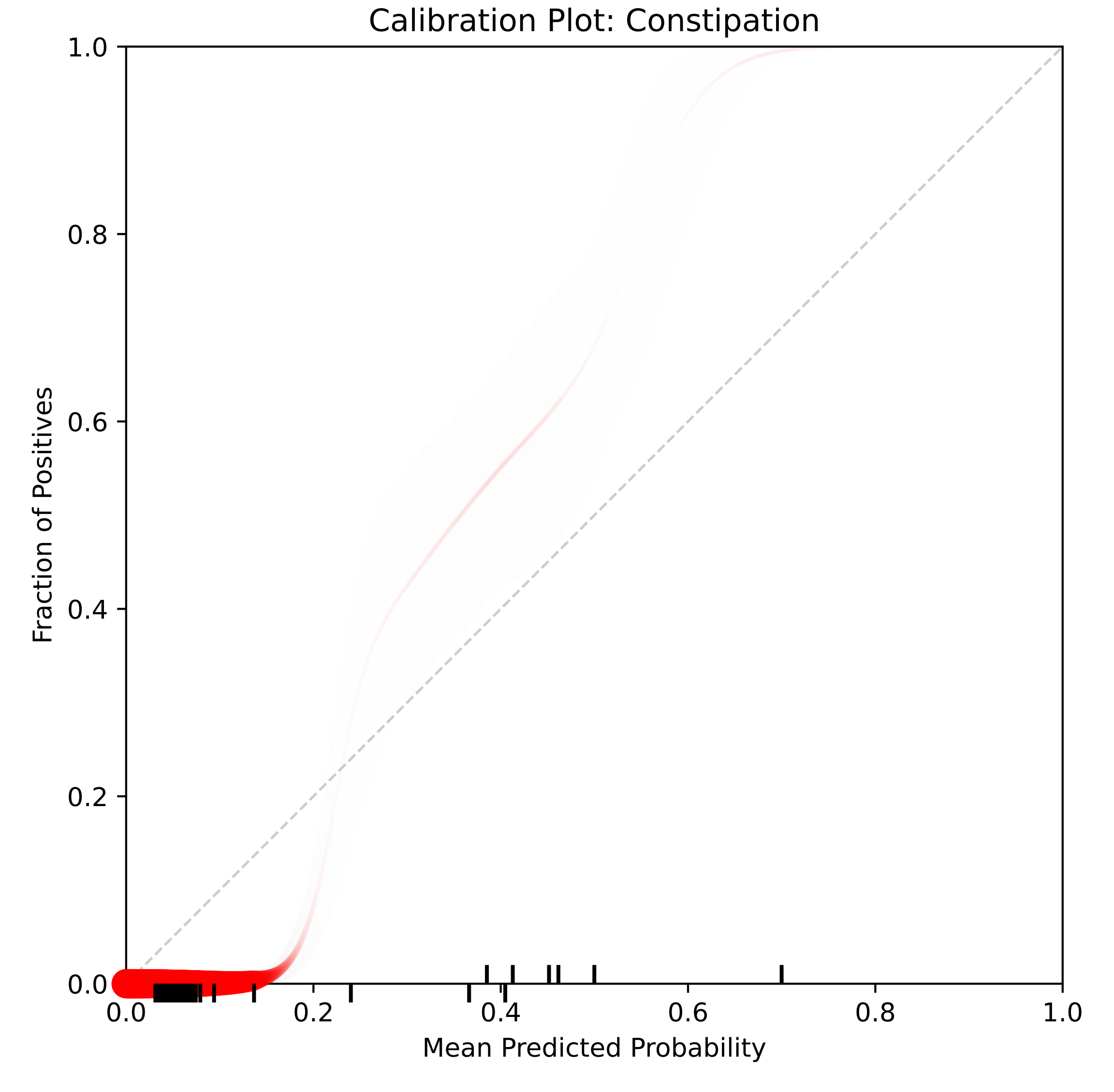

Calibration Plot: Constipation
Fraction of Positives
Mean Predicted Probability
0.0
0.2
0.4
0.6
0.8
1.0
0.0
0.2
0.4
0.6
0.8
1.0

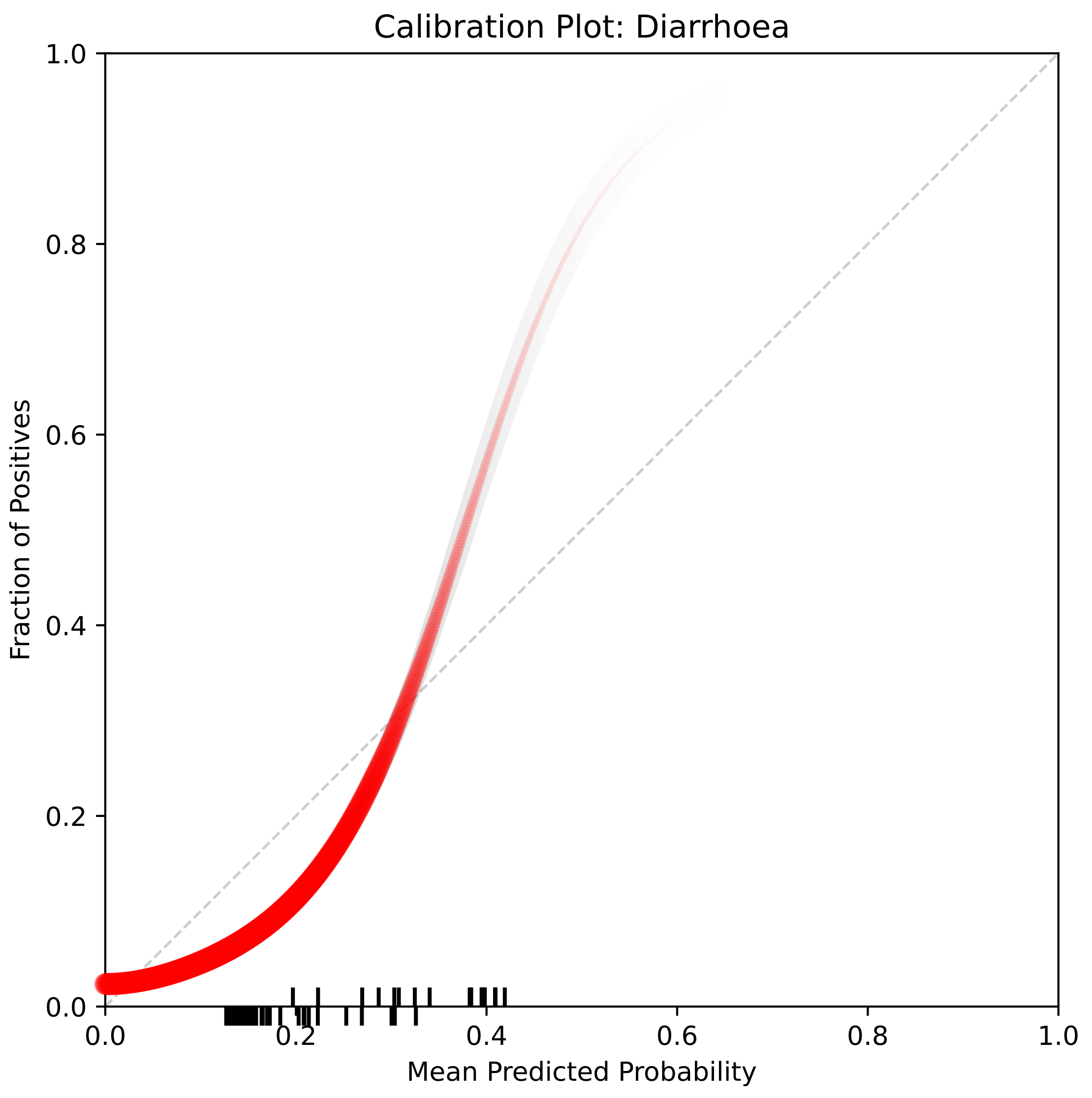
Calibration Plot: Diarrhoea
Fraction of Positives
Mean Predicted Probability
0.0
0.2
0.4
0.6
0.8
1.0

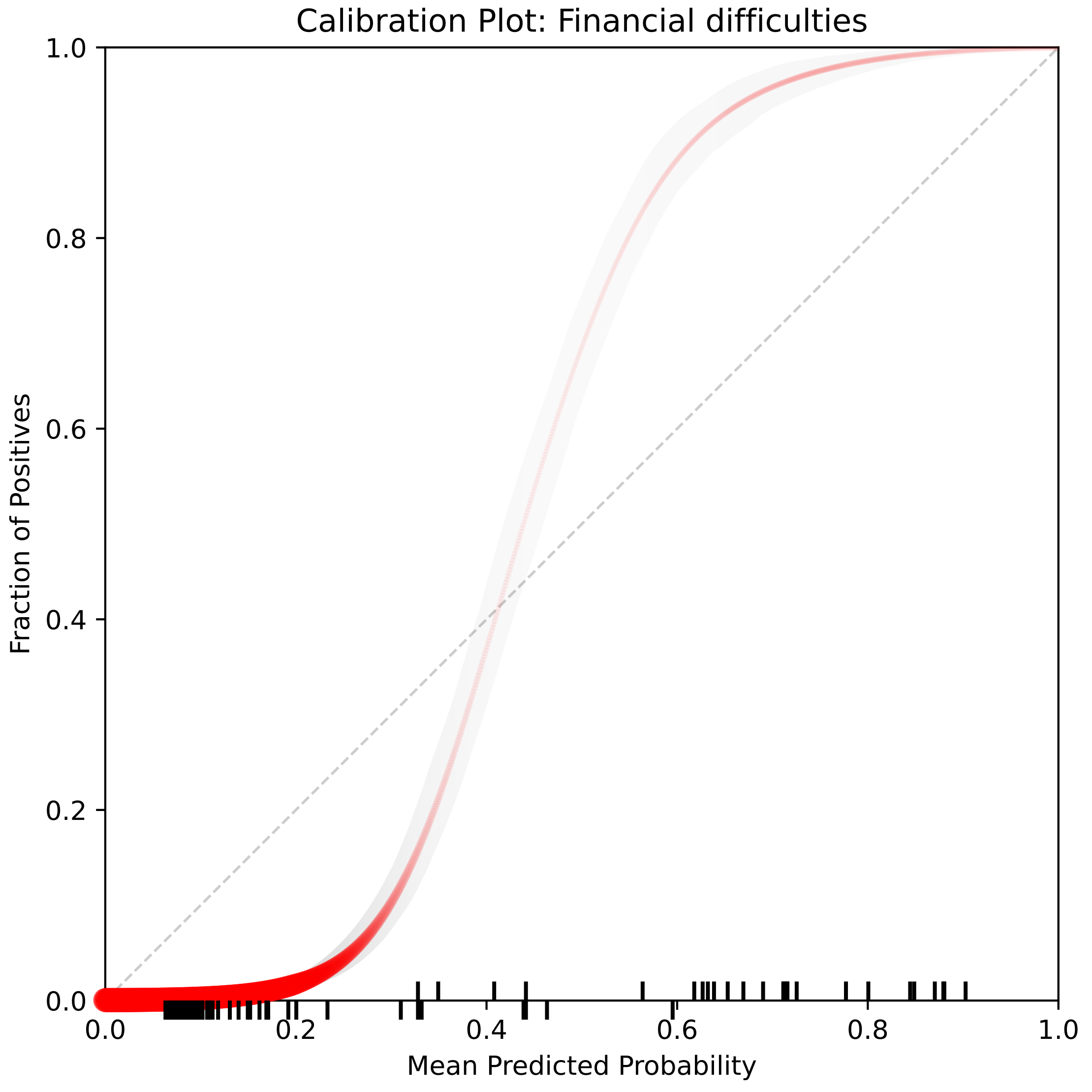

Calibration Plot: Financial difficulties
Fraction of Positives
Mean Predicted Probability
0.0
0.2
0.4
0.6
0.8
1.0

Figure S4. Feature (permutation) importance.

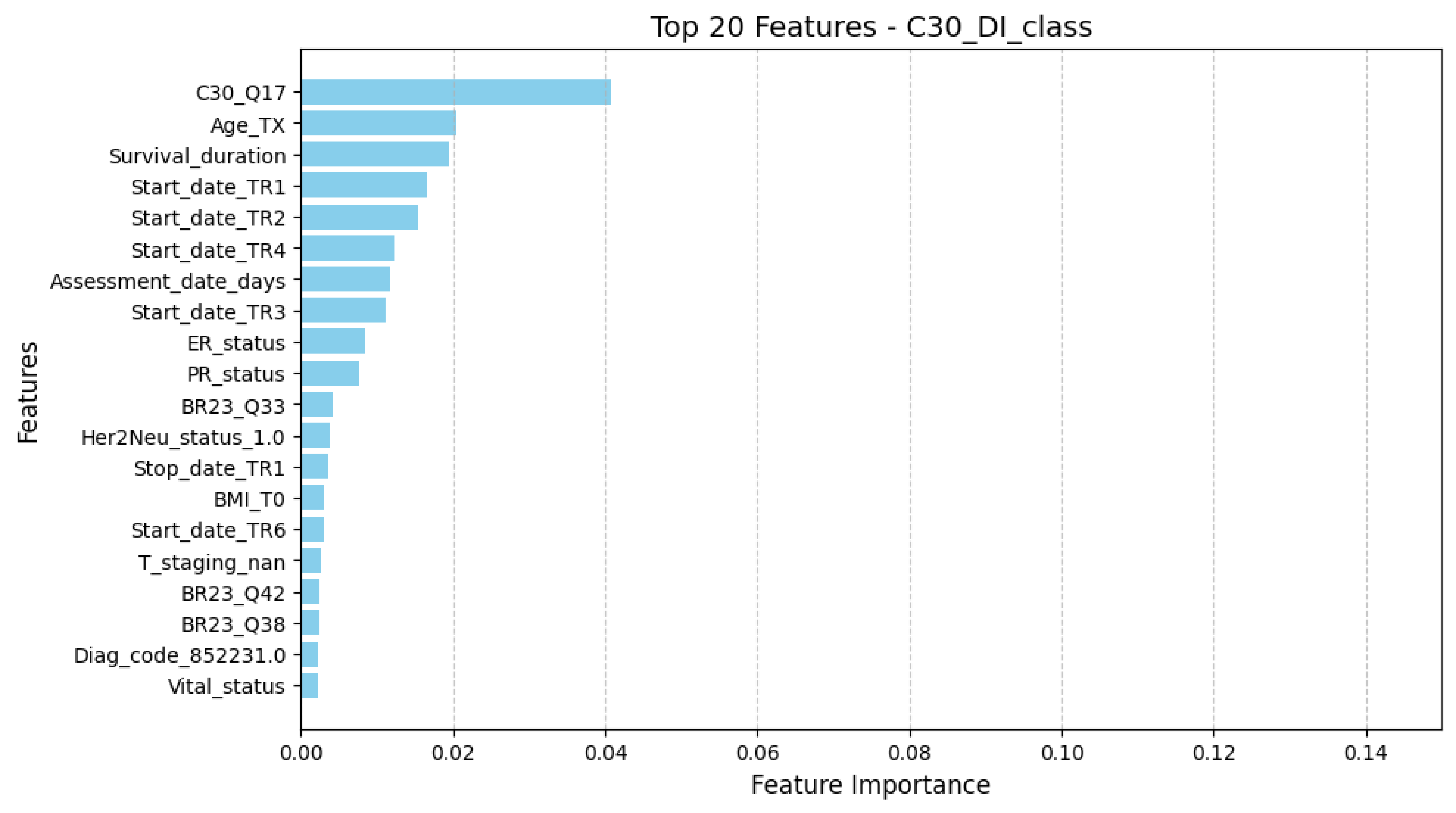

Top 20 Features - C30_DI_class
C30_Q17
Age_TX
Survival_duration
Start_date_TR1
Start_date_TR2
Start_date_TR4
Assessment_date_days
Start_date_TR3
ER_status
PR_status
BR23_Q33
Her2Neu_status_1.0
Stop_date_TR1
BMI_T0
Start_date_TR6
T_staging_nan
BR23_Q42
BR23_Q38
Diag_code_852231.0
Vital_status
Features
0.00
0.02
0.04
0.06
0.08
0.10
0.12
0.14
Feature Importance

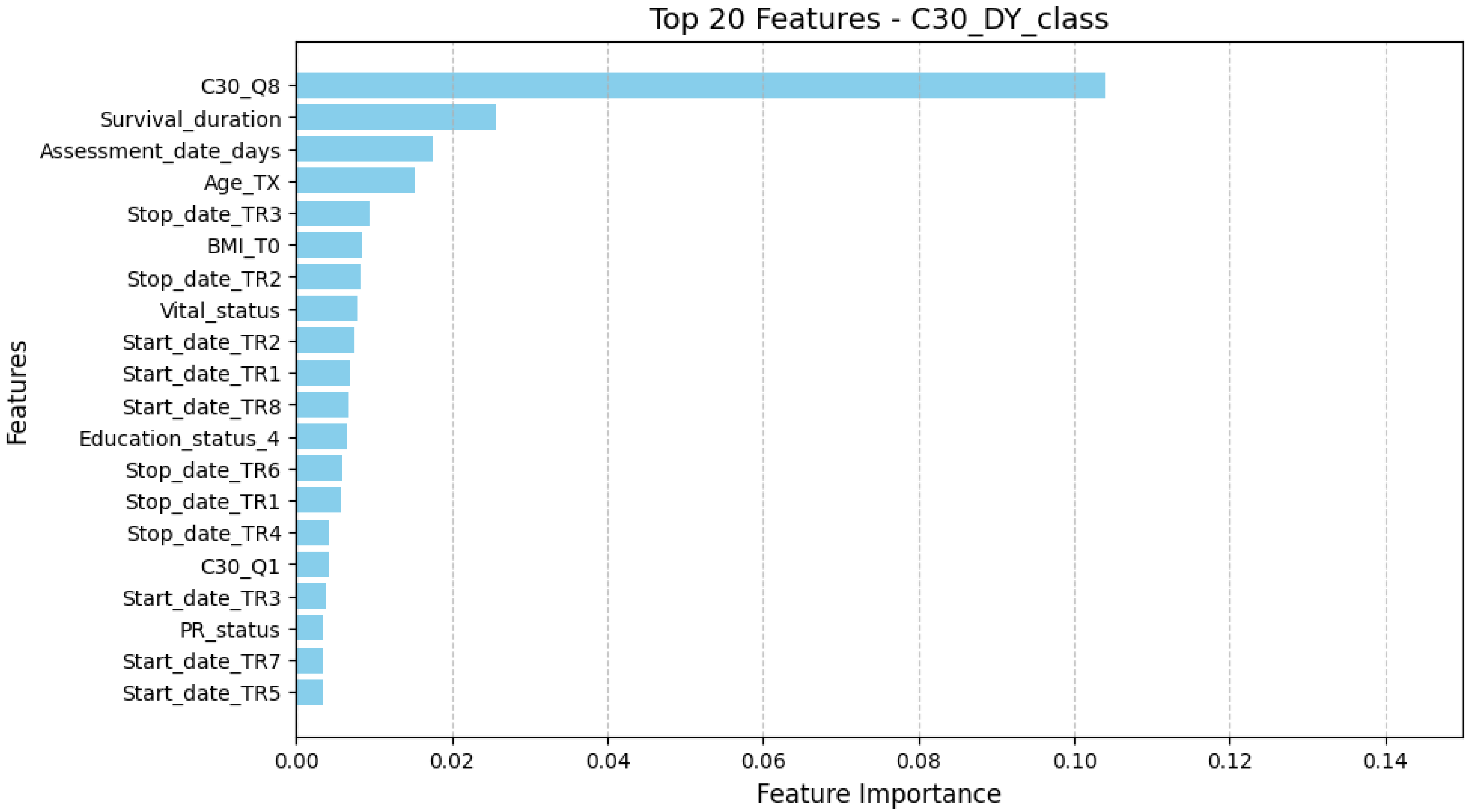
Top 20 Features - C30_DY_class
C30_Q8
Survival_duration
Assessment_date_days
Age_TX
Stop_date_TR3
BMI_T0
Stop_date_TR2
Vital_status
Start_date_TR2
Start_date_TR1
Start_date_TR8
Education_status_4
Stop_date_TR6
Stop_date_TR1
Stop_date_TR4
C30_Q1
Start_date_TR3
PR_status
Start_date_TR7
Start_date_TR5
Features
0.00
0.02
0.04
0.06
0.08
0.10
0.12
0.14
Feature Importance

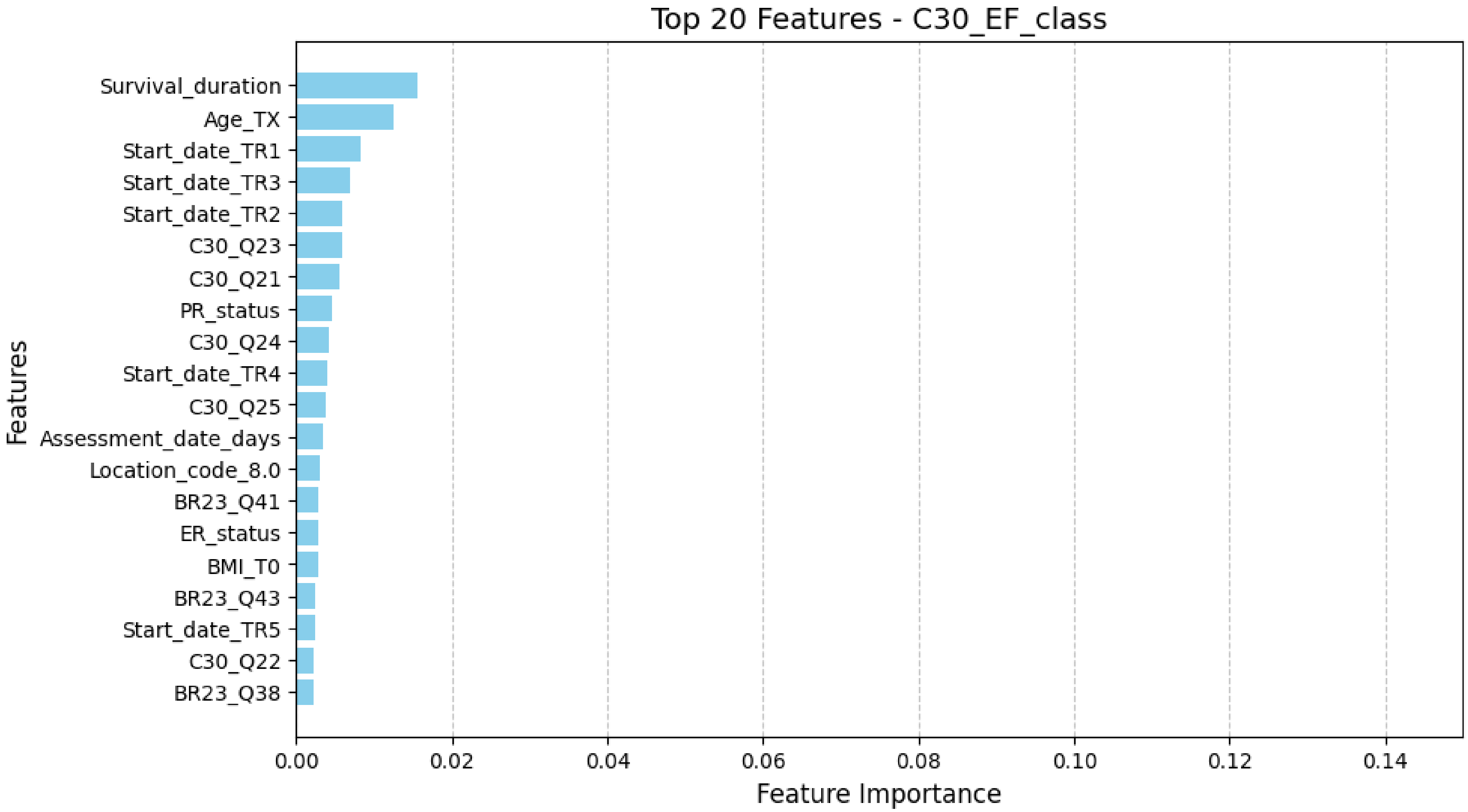
Top 20 Features - C30_EF_class
Survival_duration
Age_TX
Start_date_TR1
Start_date_TR3
Start_date_TR2
C30_Q23
C30_Q21
PR_status
C30_Q24
Start_date_TR4
C30_Q25
Assessment_date_days
Location_code_8.0
BR23_Q41
ER_status
BMI_T0
BR23_Q43
Start_date_TR5
C30_Q22
BR23_Q38
Features
0.00
0.02
0.04
0.06
0.08
0.10
0.12
0.14
Feature Importance

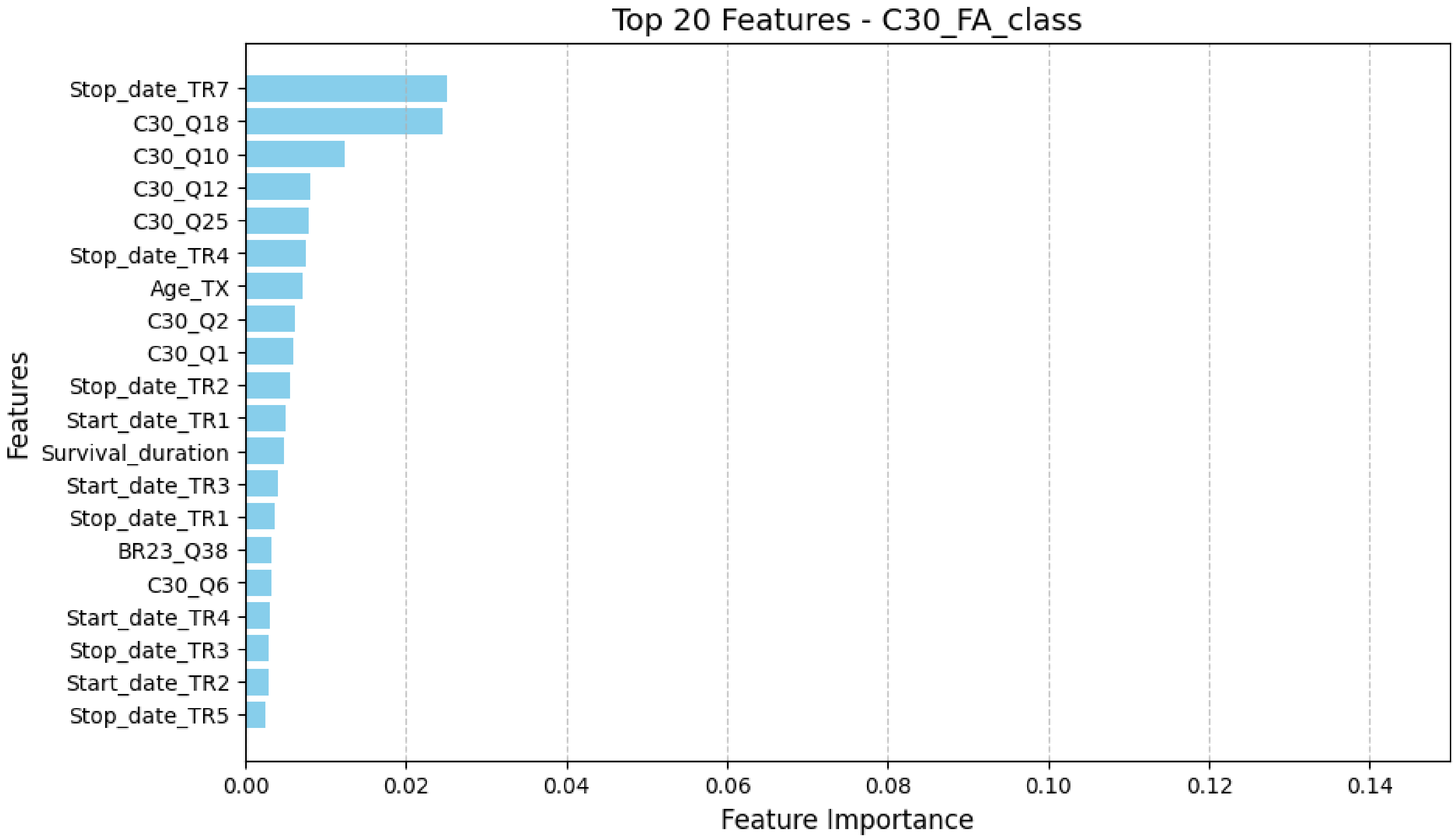

Top 20 Features - C30_FA_class
Stop_date_TR7
C30_Q18
C30_Q10
C30_Q12
C30_Q25
Stop_date_TR4
Age_TX
C30_Q2
C30_Q1
Stop_date_TR2
Start_date_TR1
Survival_duration
Start_date_TR3
Stop_date_TR1
BR23_Q38
C30_Q6
Start_date_TR4
Stop_date_TR3
Start_date_TR2
Stop_date_TR5
Features
0.00
0.02
0.04
0.06
0.08
0.10
0.12
0.14
Feature Importance

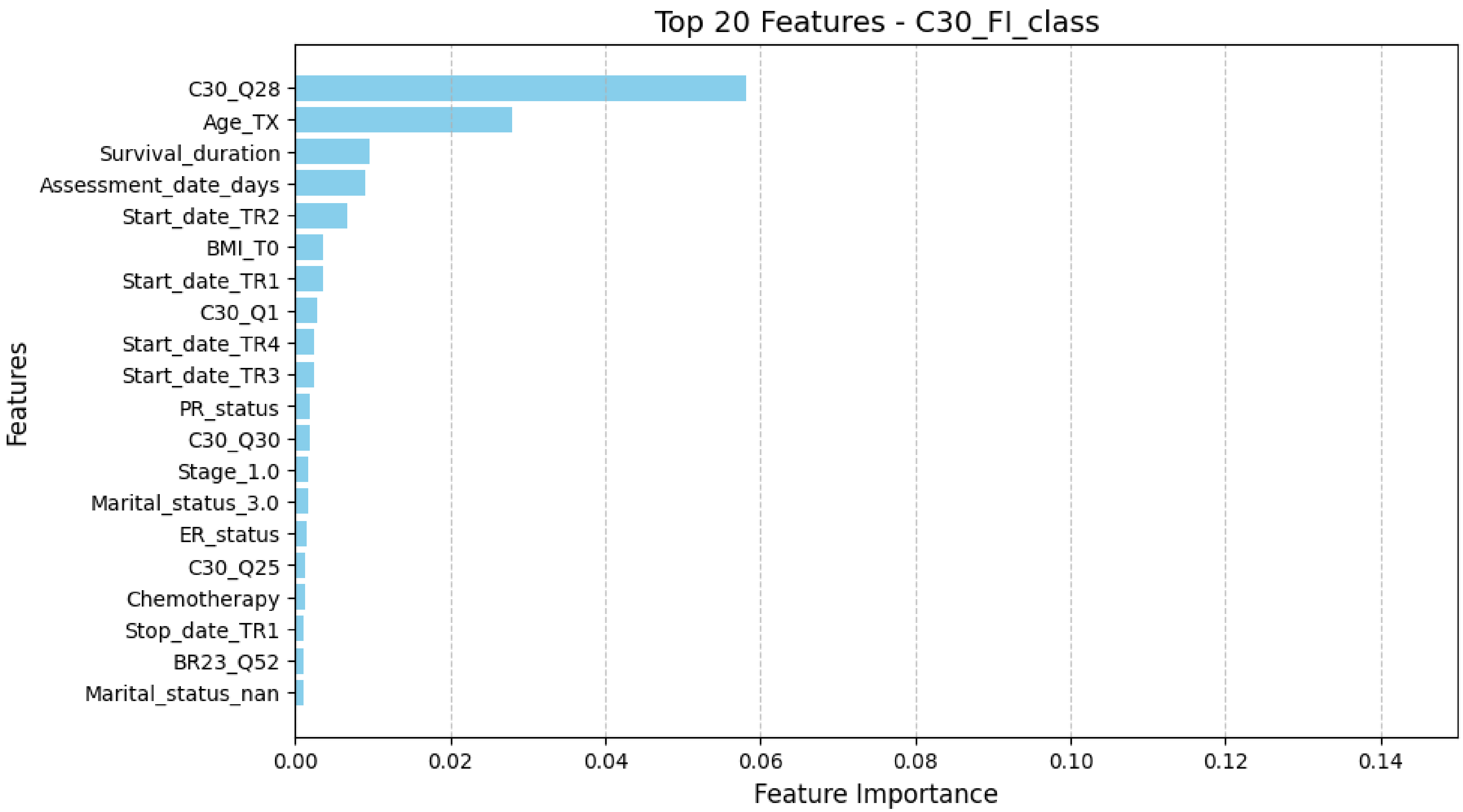
Top 20 Features - C30_FI_class
C30_Q28
Age_TX
Survival_duration
Assessment_date_days
Start_date_TR2
BMI_T0
Start_date_TR1
C30_Q1
Start_date_TR4
Start_date_TR3
PR_status
C30_Q30
Stage_1.0
Marital_status_3.0
ER_status
C30_Q25
Chemotherapy
Stop_date_TR1
BR23_Q52
Marital_status_nan
Features
0.00
0.02
0.04
0.06
0.08
0.10
0.12
0.14
Feature Importance

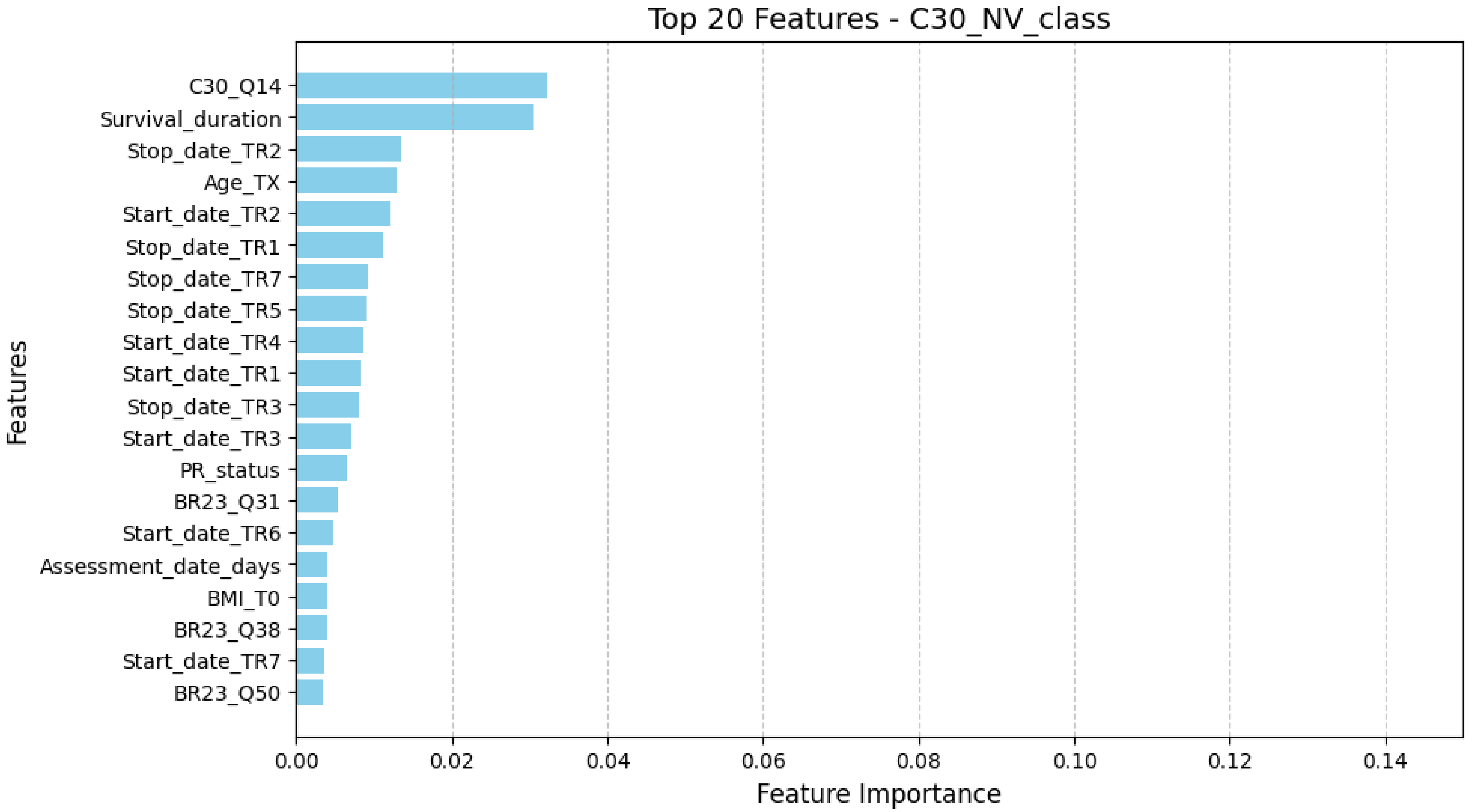

Top 20 Features - C30_NV_class
C30_Q14
Survival_duration
Stop_date_TR2
Age_TX
Start_date_TR2
Stop_date_TR1
Stop_date_TR7
Stop_date_TR5
Start_date_TR4
Start_date_TR1
Stop_date_TR3
Start_date_TR3
PR_status
BR23_Q31
Start_date_TR6
Assessment_date_days
BMI_T0
BR23_Q38
Start_date_TR7
BR23_Q50
Features
0.00
0.02
0.04
0.06
0.08
0.10
0.12
0.14
Feature Importance

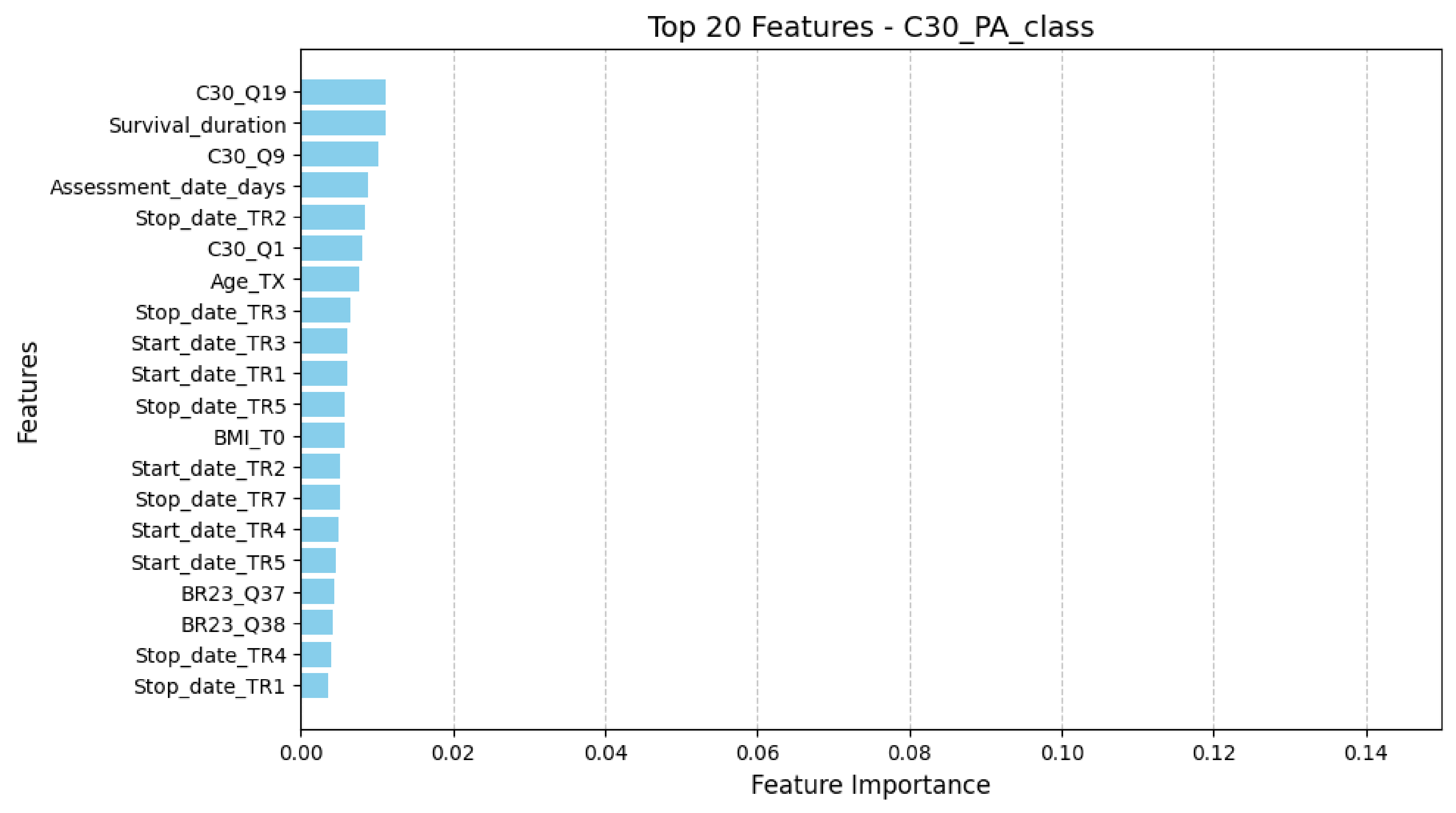

Top 20 Features - C30_PA_class
C30_Q19
Survival_duration
C30_Q9
Assessment_date_days
Stop_date_TR2
C30_Q1
Age_TX
Stop_date_TR3
Start_date_TR3
Start_date_TR1
Stop_date_TR5
BMI_T0
Start_date_TR2
Stop_date_TR7
Start_date_TR4
Start_date_TR5
BR23_Q37
BR23_Q38
Stop_date_TR4
Stop_date_TR1
Features
0.00
0.02
0.04
0.06
0.08
0.10
0.12
0.14
Feature Importance

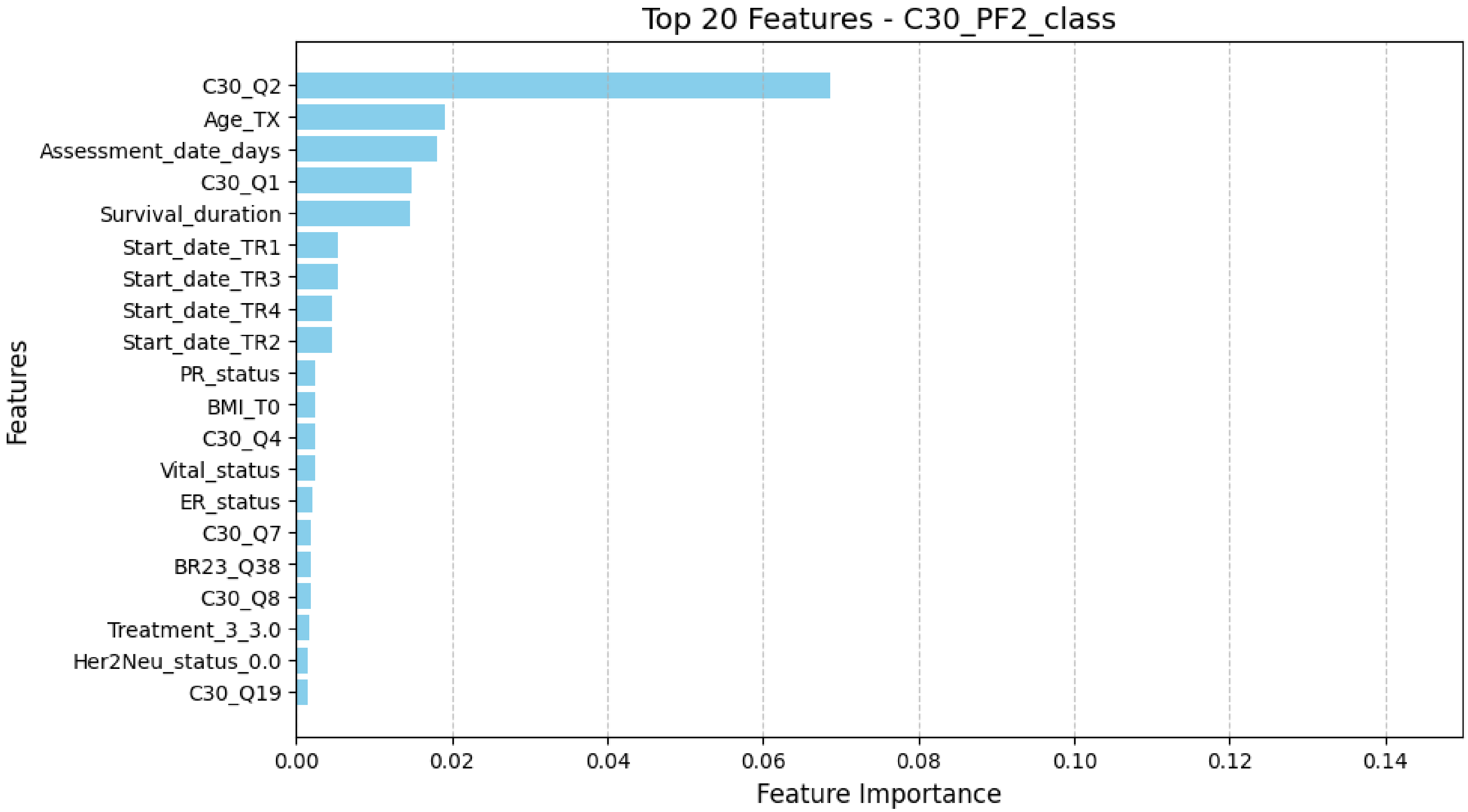
Top 20 Features - C30_PF2_class
C30_Q2
Age_TX
Assessment_date_days
C30_Q1
Survival_duration
Start_date_TR1
Start_date_TR3
Start_date_TR4
Start_date_TR2
PR_status
BMI_T0
C30_Q4
Vital_status
ER_status
C30_Q7
BR23_Q38
C30_Q8
Treatment_3_3.0
Her2Neu_status_0.0
C30_Q19
Features
0.00
0.02
0.04
0.06
0.08
0.10
0.12
0.14
Feature Importance

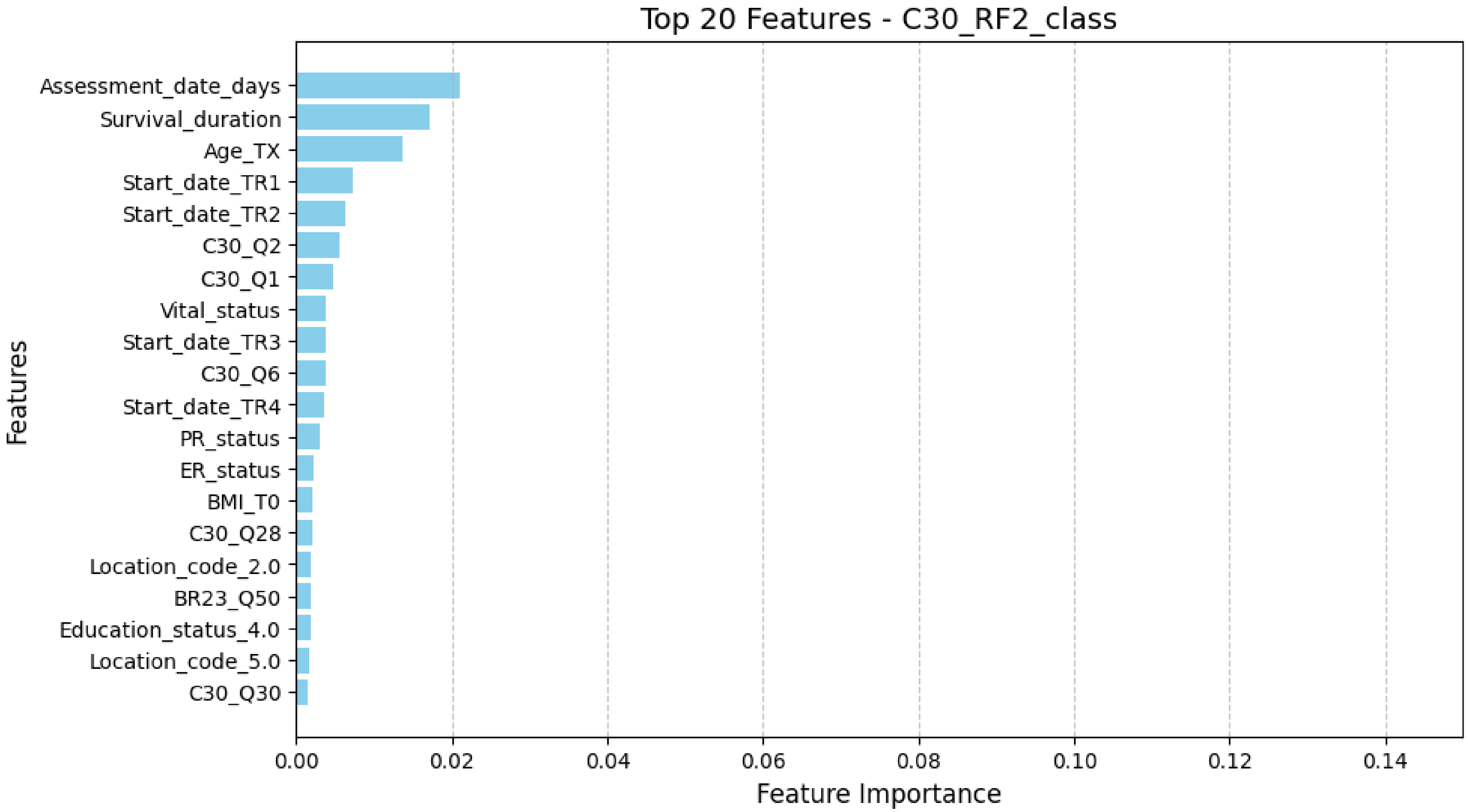

Top 20 Features - C30_RF2_class
Assessment_date_days
Survival_duration
Age_TX
Start_date_TR1
Start_date_TR2
C30_Q2
C30_Q1
Vital_status
Start_date_TR3
C30_Q6
Start_date_TR4
PR_status
ER_status
BMI_T0
C30_Q28
Location_code_2.0
BR23_Q50
Education_status_4.0
Location_code_5.0
C30_Q30
Features
0.00
0.02
0.04
0.06
0.08
0.10
0.12
0.14
Feature Importance

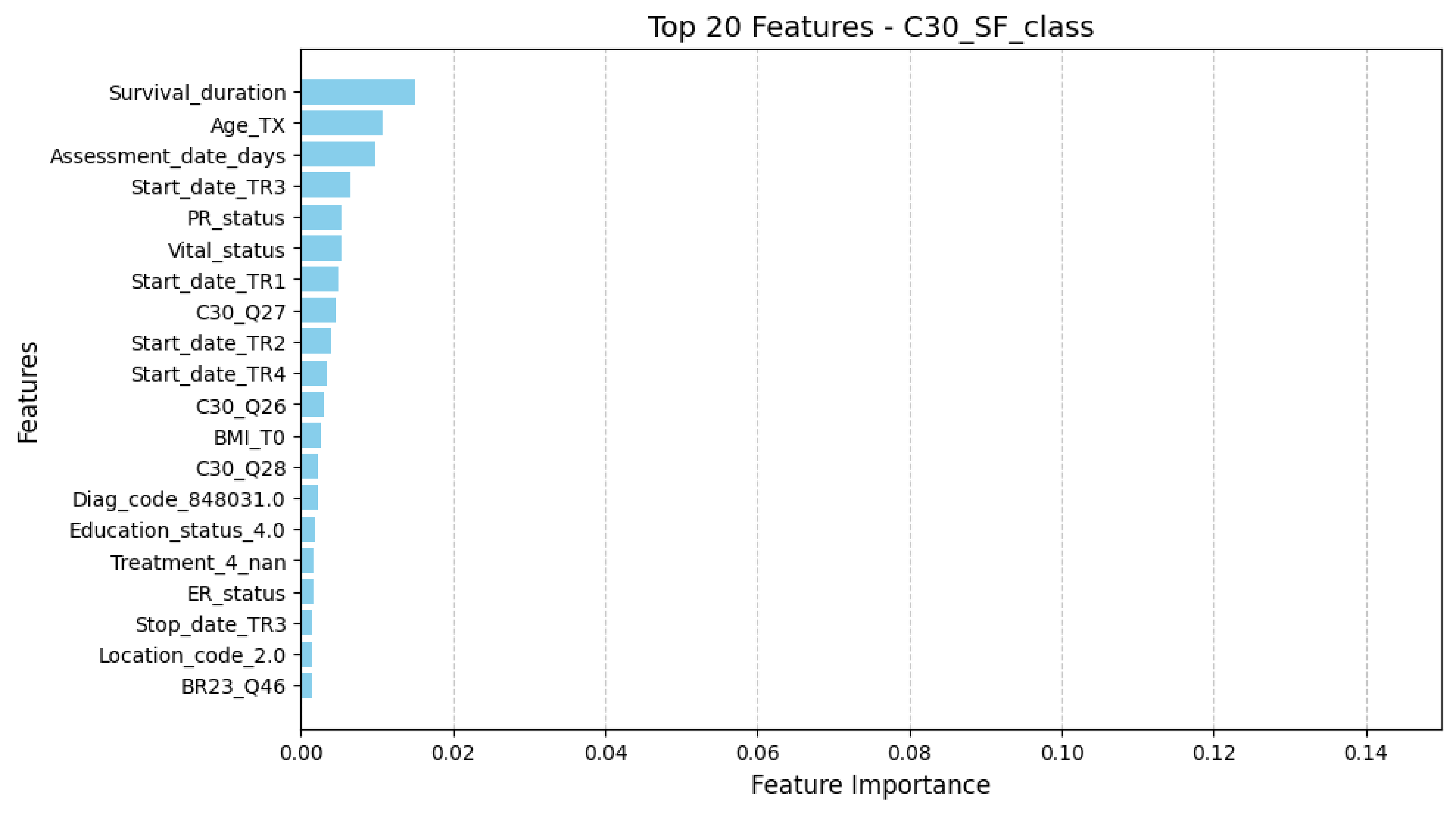
Top 20 Features - C30_SF_class
Survival_duration
Age_TX
Assessment_date_days
Start_date_TR3
PR_status
Vital_status
Start_date_TR1
C30_Q27
Start_date_TR2
Start_date_TR4
C30_Q26
BMI_T0
C30_Q28
Diag_code_848031.0
Education_status_4.0
Treatment_4_nan
ER_status
Stop_date_TR3
Location_code_2.0
BR23_Q46
Features
0.00
0.02
0.04
0.06
0.08
0.10
0.12
0.14
Feature Importance

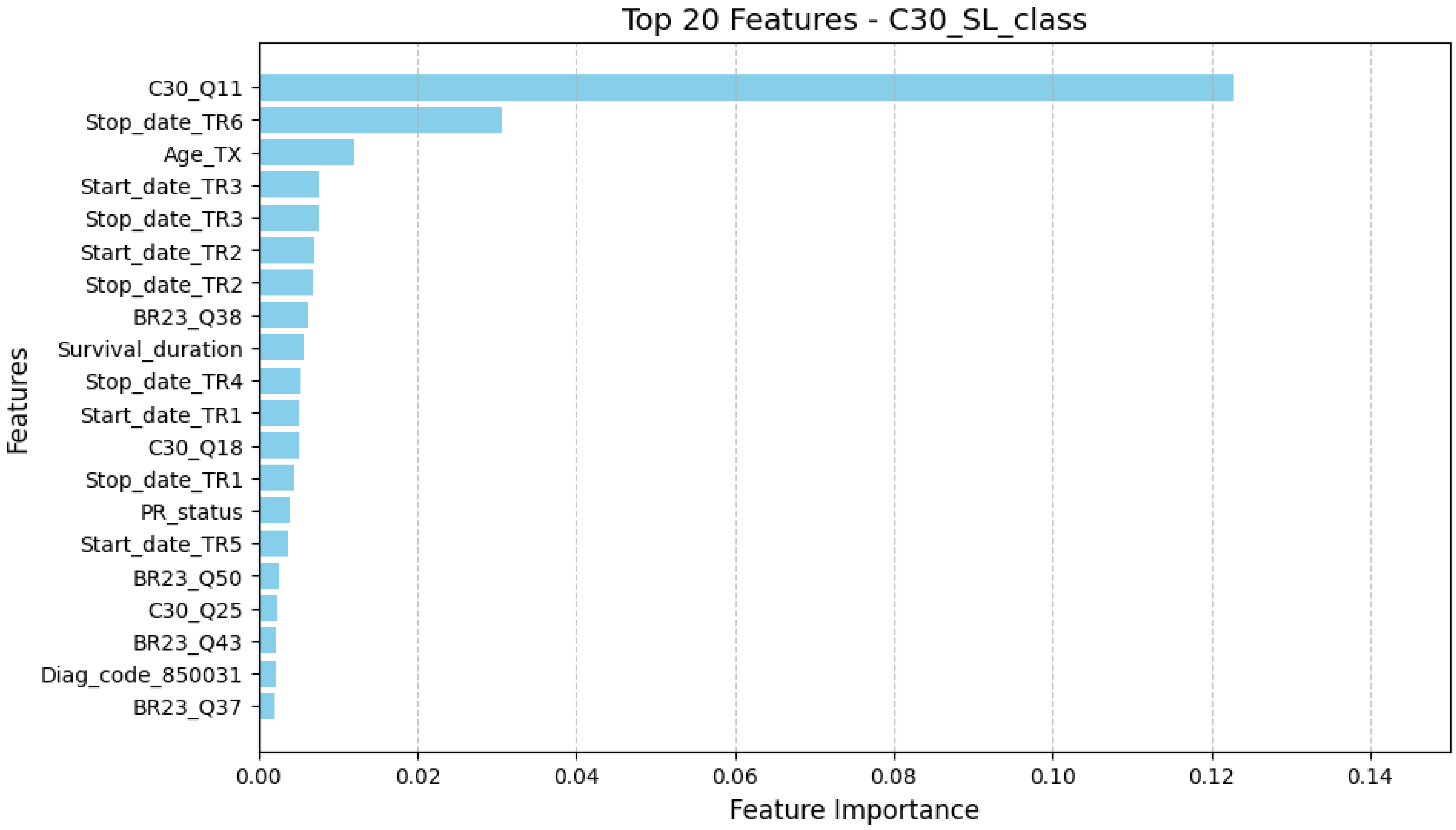
Top 20 Features - C30_SL_class
C30_Q11
Stop_date_TR6
Age_TX
Start_date_TR3
Stop_date_TR3
Start_date_TR2
Stop_date_TR2
BR23_Q38
Survival_duration
Stop_date_TR4
Start_date_TR1
C30_Q18
Stop_date_TR1
PR_status
Start_date_TR5
BR23_Q50
C30_Q25
BR23_Q43
Diag_code_850031
BR23_Q37
Features
0.00
0.02
0.04
0.06
0.08
0.10
0.12
0.14
Feature Importance

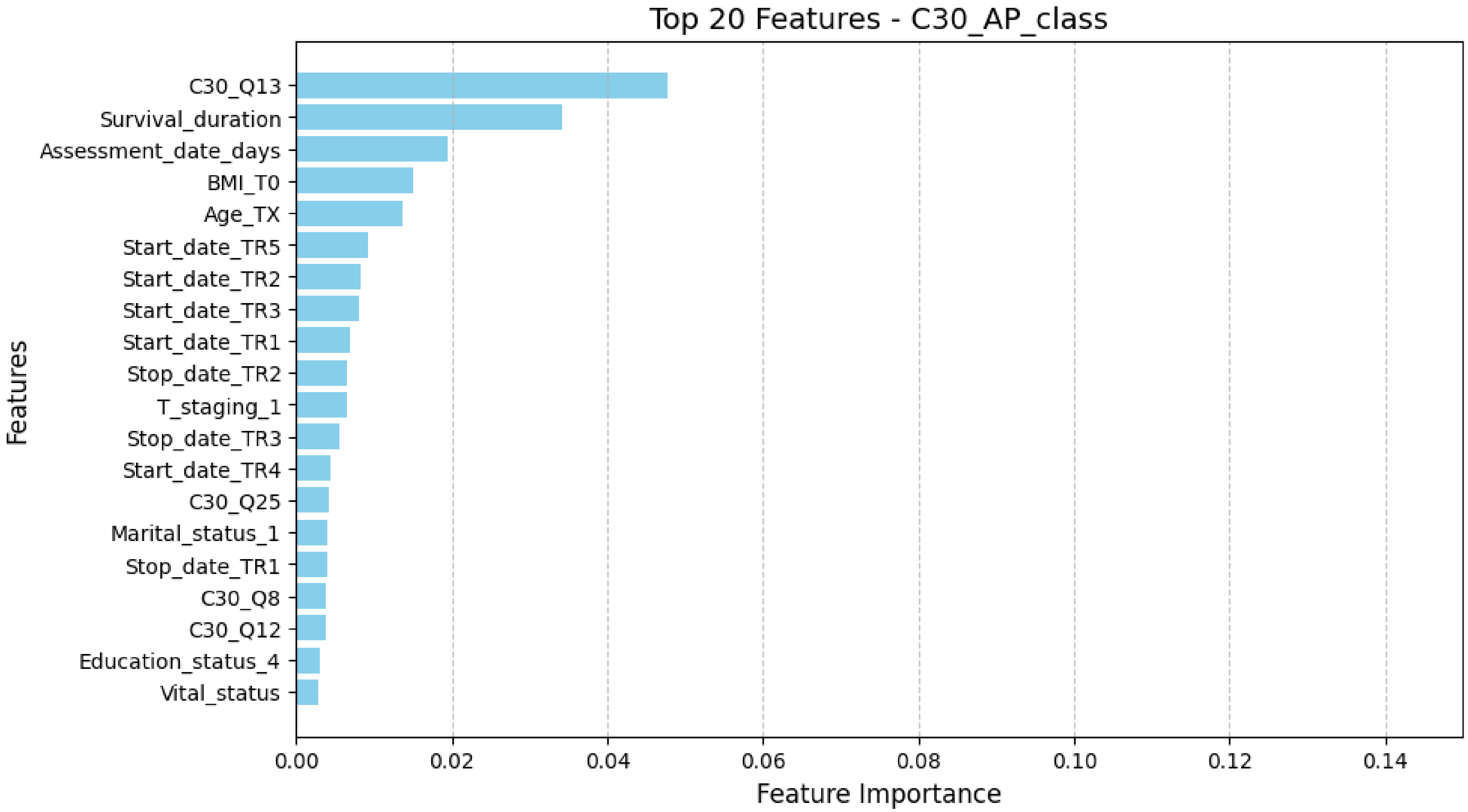
Top 20 Features - C30_AP_class
C30_Q13
Survival_duration
Assessment_date_days
BMI_T0
Age_TX
Start_date_TR5
Start_date_TR2
Start_date_TR3
Start_date_TR1
Stop_date_TR2
T_staging_1
Stop_date_TR3
Start_date_TR4
C30_Q25
Marital_status_1
Stop_date_TR1
C30_Q8
C30_Q12
Education_status_4
Vital_status
Features
0.00
0.02
0.04
0.06
0.08
0.10
0.12
0.14
Feature Importance

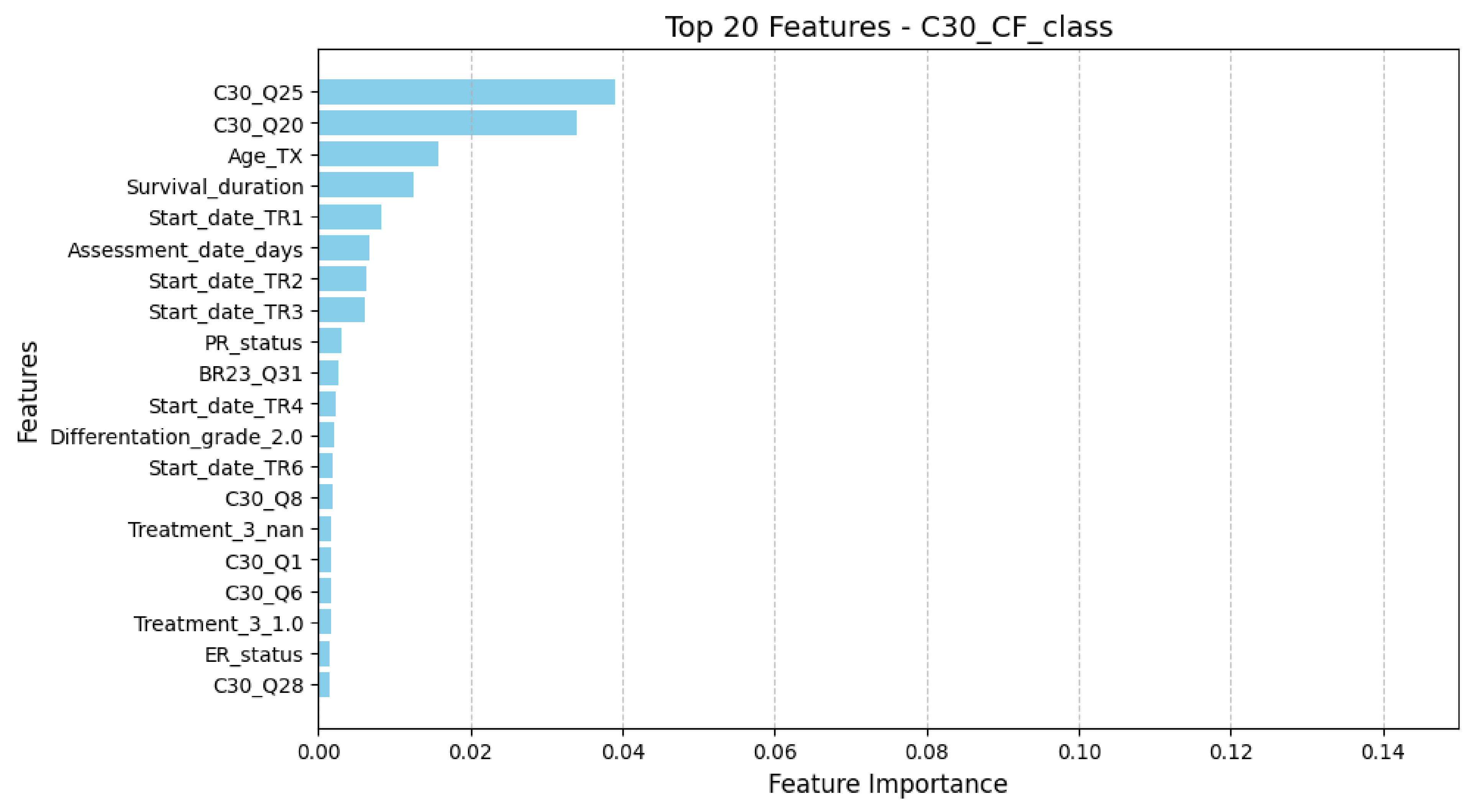

Top 20 Features - C30_CF_class
C30_Q25
C30_Q20
Age_TX
Survival_duration
Start_date_TR1
Assessment_date_days
Start_date_TR2
Start_date_TR3
PR_status
BR23_Q31
Start_date_TR4
Differentation_grade_2.0
Start_date_TR6
C30_Q8
Treatment_3_nan
C30_Q1
C30_Q6
Treatment_3_1.0
ER_status
C30_Q28
Features
0.00
0.02
0.04
0.06
0.08
0.10
0.12
0.14
Feature Importance

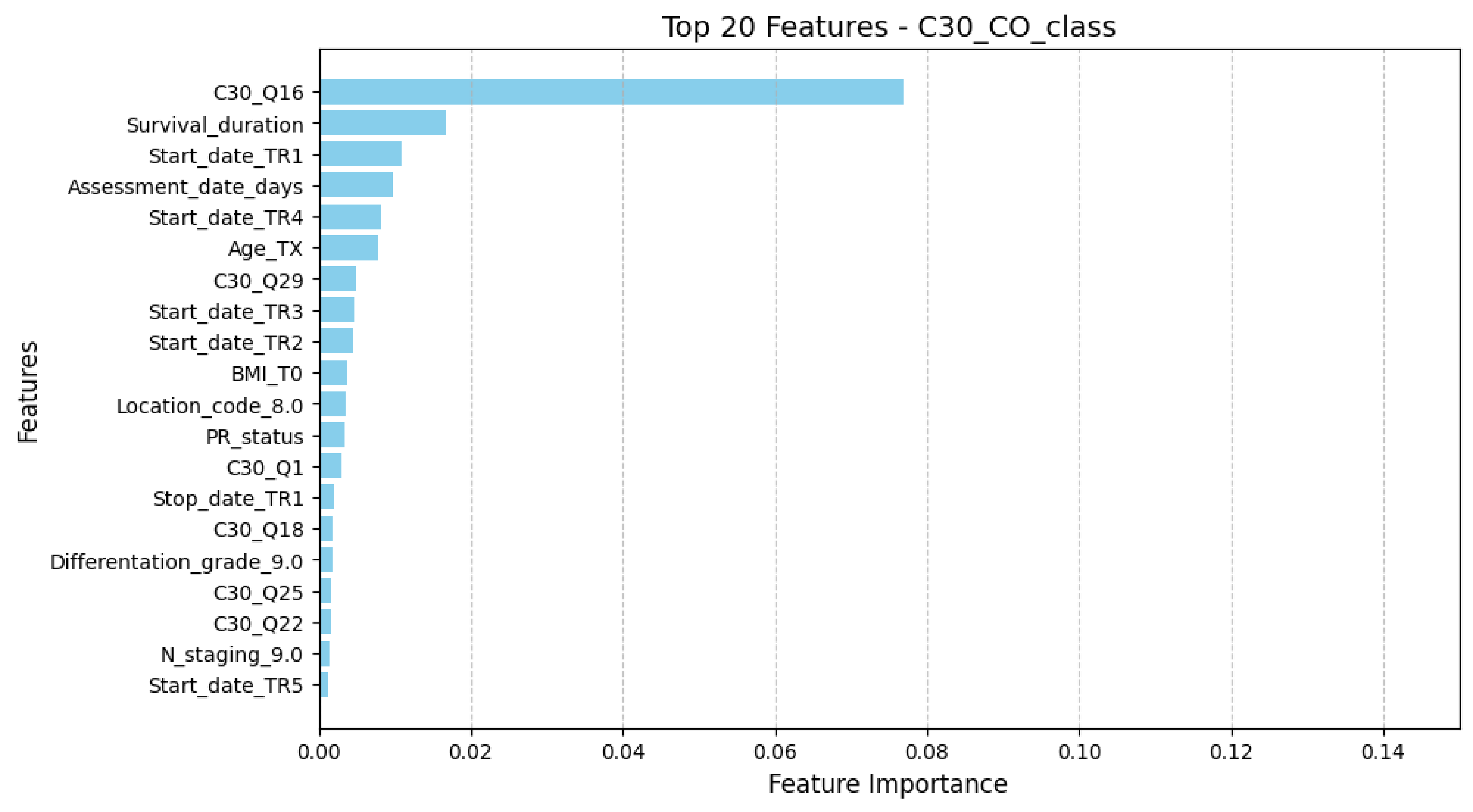

Top 20 Features - C30_CO_class
C30_Q16
Survival_duration
Start_date_TR1
Assessment_date_days
Start_date_TR4
Age_TX
C30_Q29
Start_date_TR3
Start_date_TR2
BMI_T0
Location_code_8.0
PR_status
C30_Q1
Stop_date_TR1
C30_Q18
Differentation_grade_9.0
C30_Q25
C30_Q22
N_staging_9.0
Start_date_TR5
Features
0.00
0.02
0.04
0.06
0.08
0.10
0.12
0.14
Feature Importance

Figure S5. Decision curve analyses.

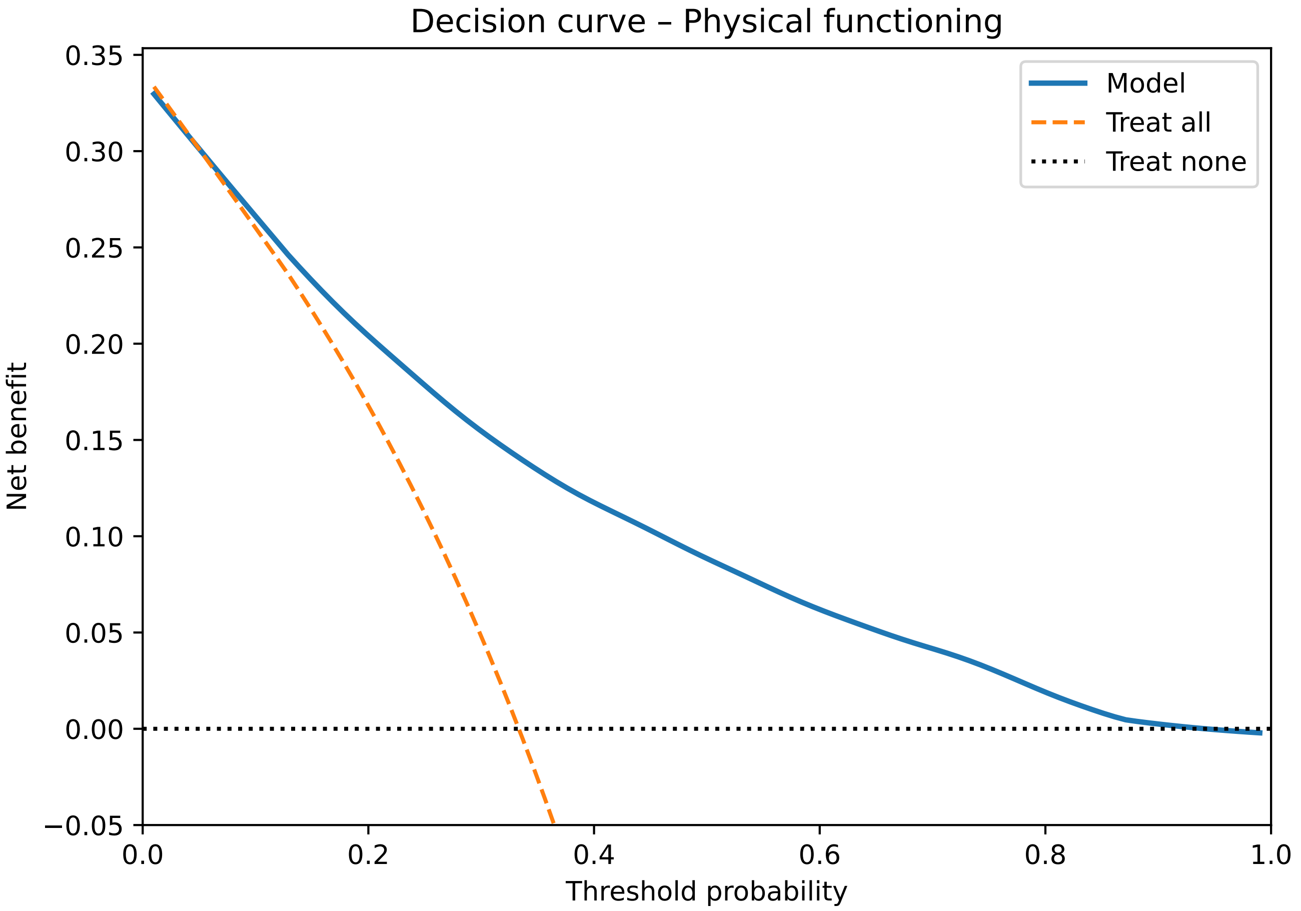

Decision curve – Physical functioning
Model
Treat all
Treat none
Net benefit
Threshold probability
0.35
0.30
0.25
0.20
0.15
0.10
0.05
0.00
−0.05
0.0
0.2
0.4
0.6
0.8
1.0

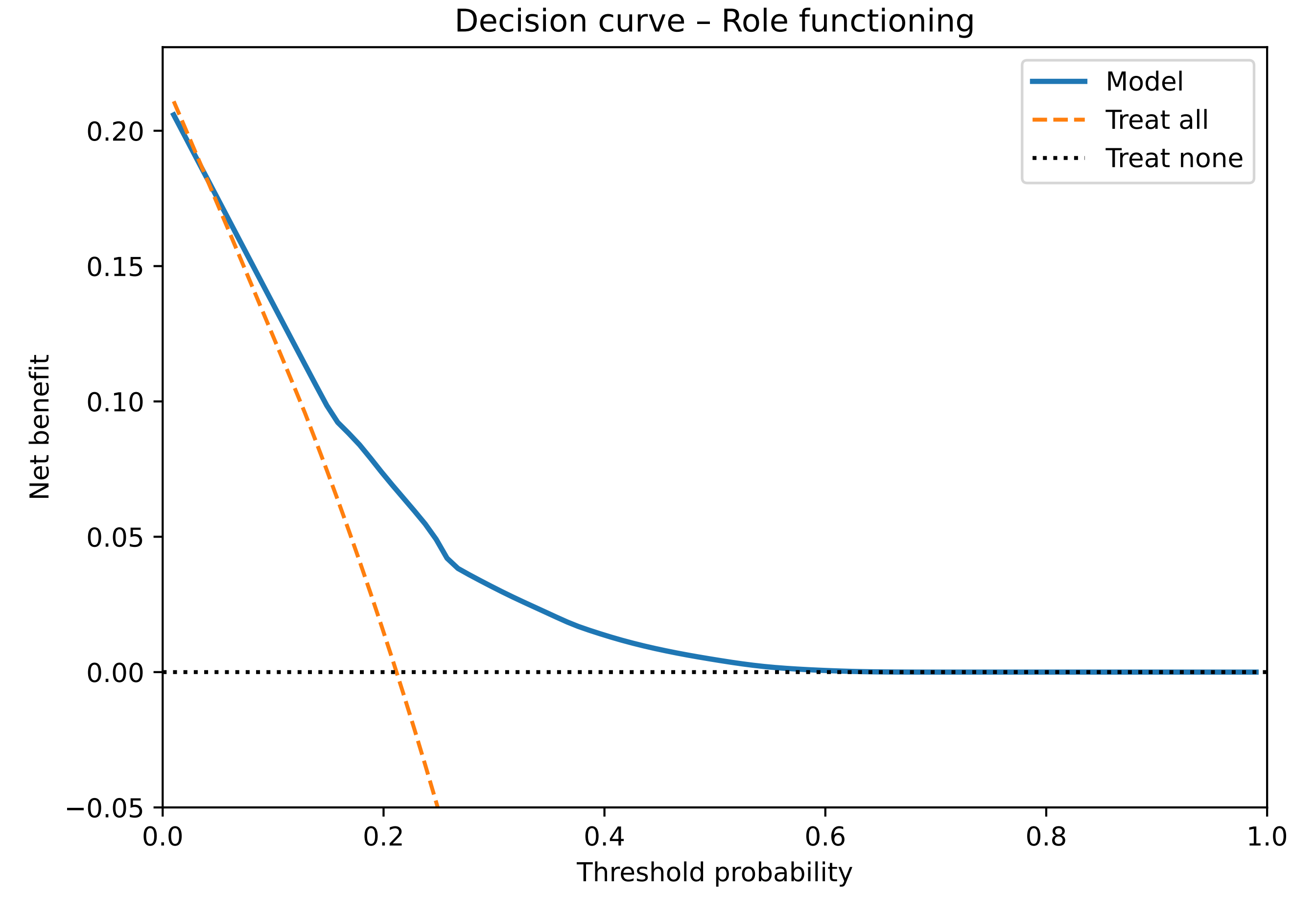

Decision curve – Role functioning
Model
Treat all
Treat none
Net benefit
Threshold probability
0.20
0.15
0.10
0.05
0.00
−0.05
0.0
0.2
0.4
0.6
0.8
1.0

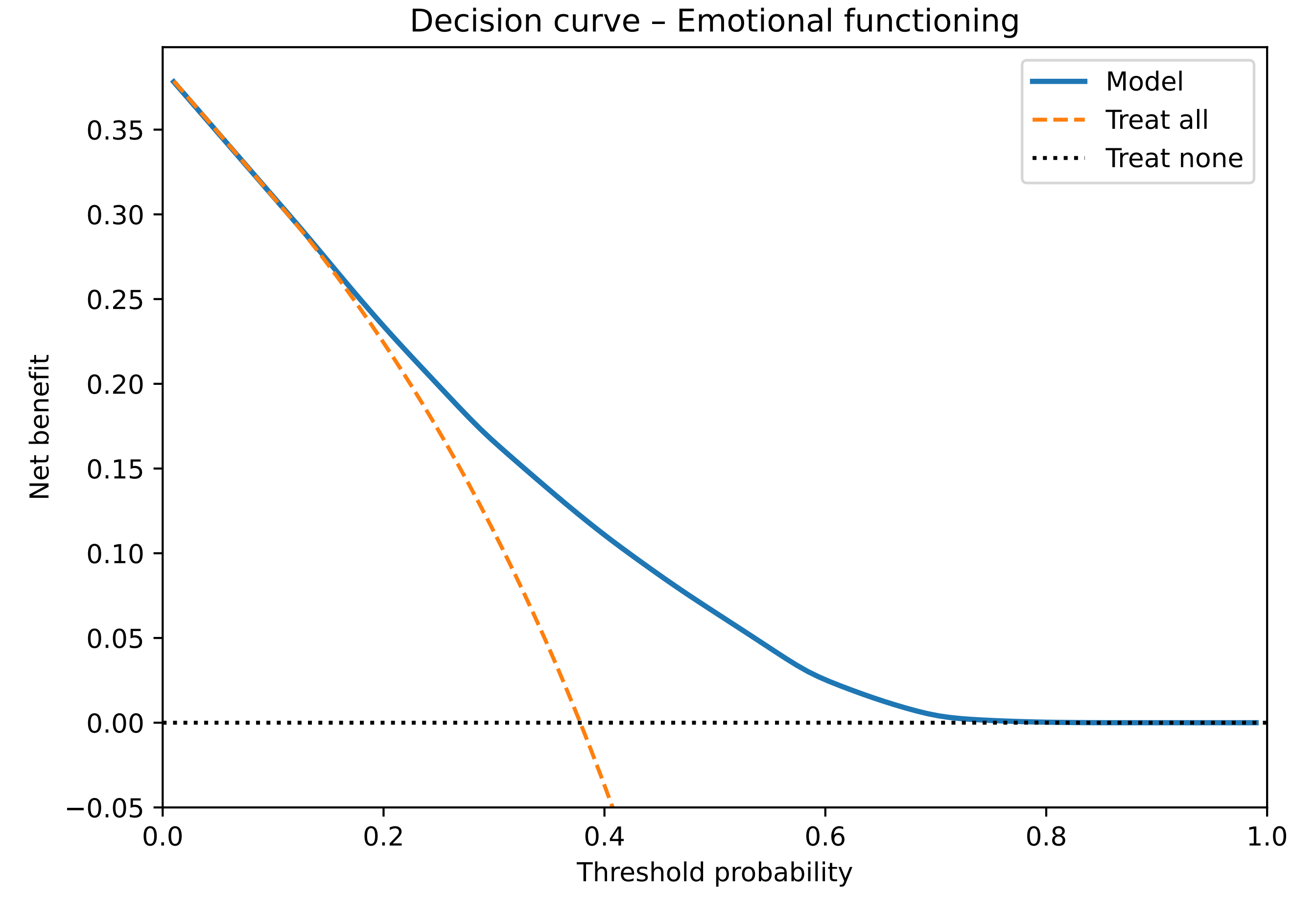

Decision curve – Emotional functioning
Model
Treat all
Treat none
Net benefit
Threshold probability
0.35
0.30
0.25
0.20
0.15
0.10
0.05
0.00
−0.05
0.0
0.2
0.4
0.6
0.8
1.0

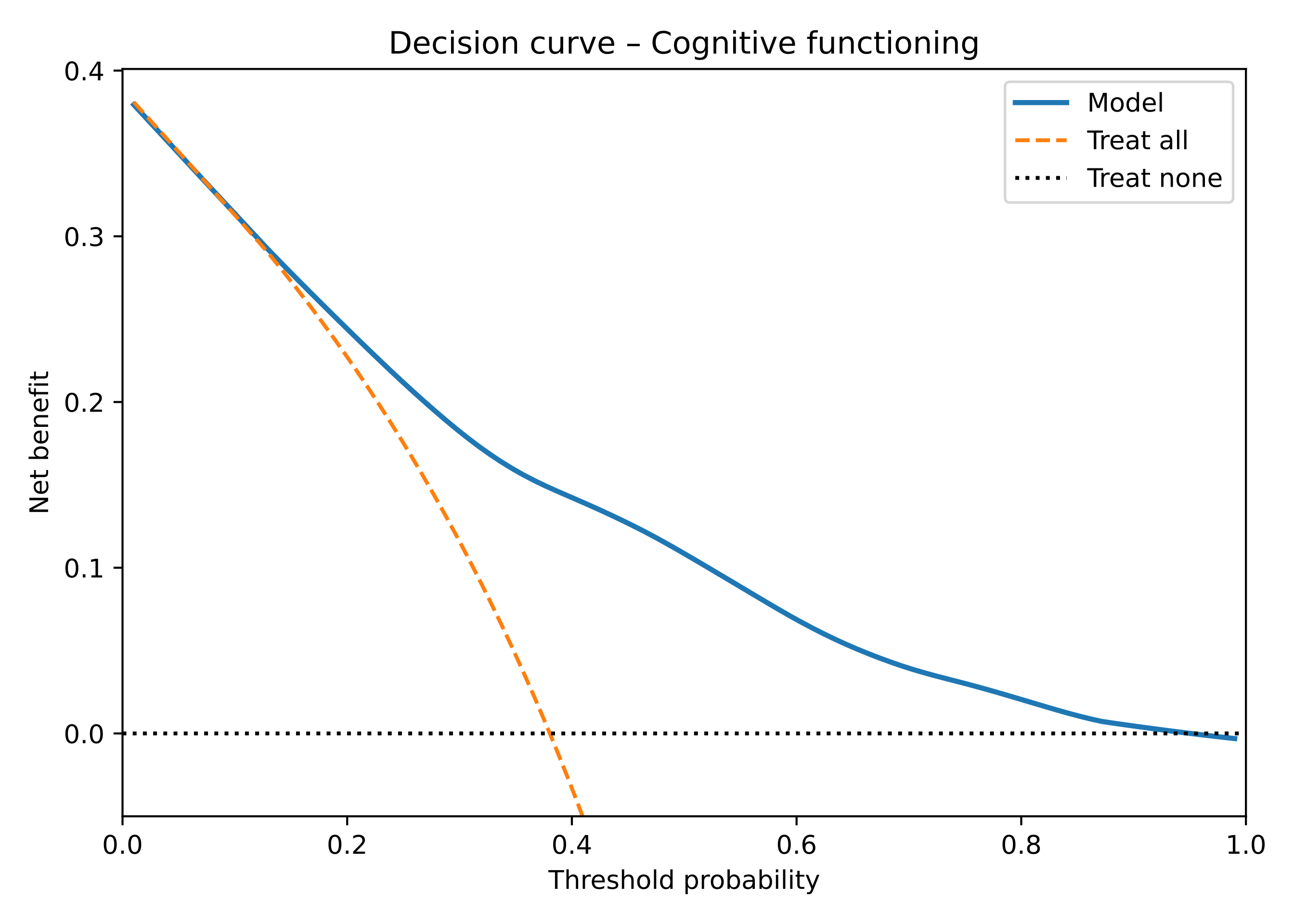

Decision curve – Cognitive functioning
Net benefit
Threshold probability
Model
Treat all
Treat none
0.4
0.3
0.2
0.1
0.0
0.0
0.2
0.4
0.6
0.8
1.0

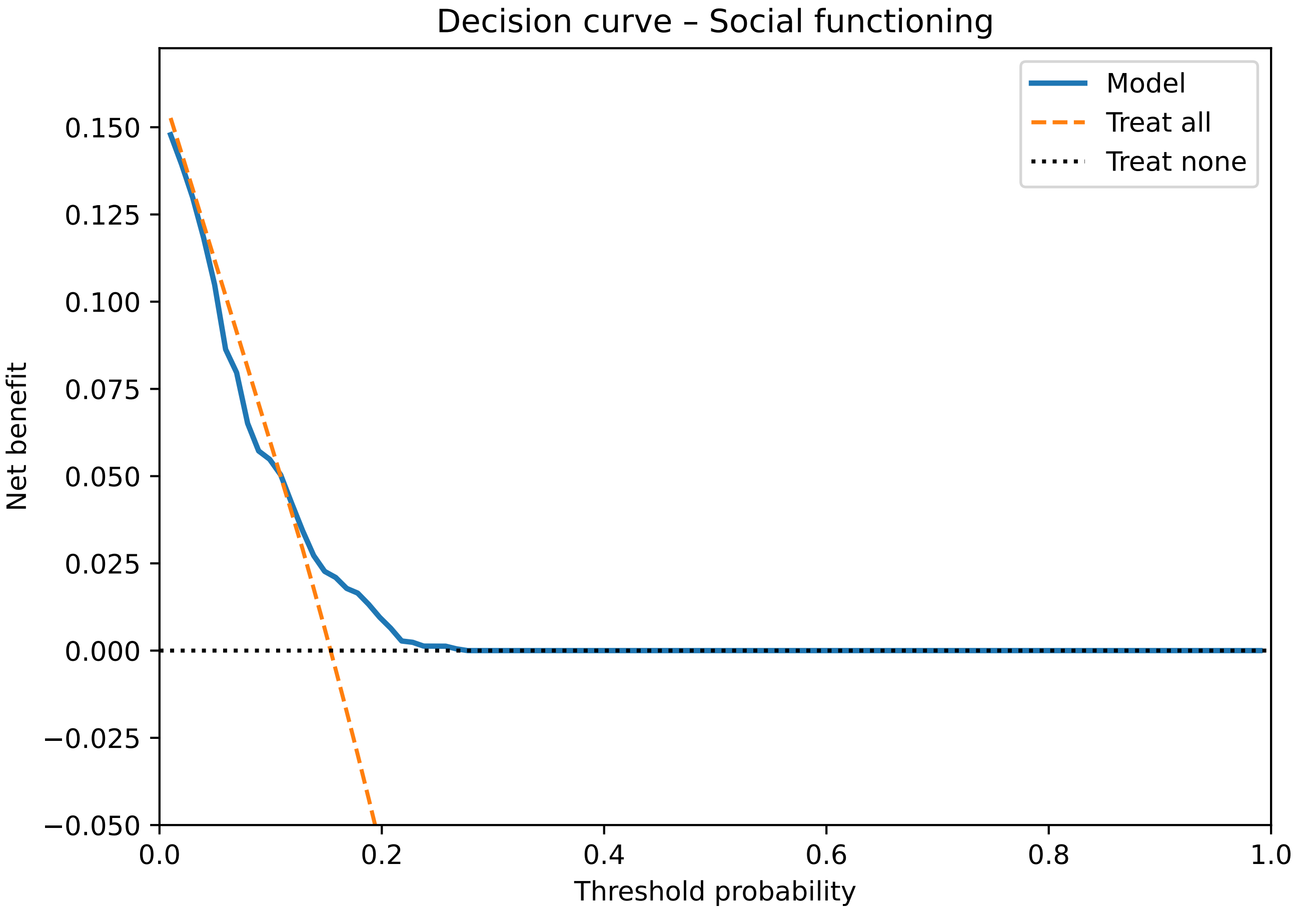
Decision curve – Social functioning
Model
Treat all
Treat none
Net benefit
Threshold probability
0.150
0.125
0.100
0.075
0.050
0.025
0.000
−0.025
−0.050
0.0
0.2
0.4
0.6
0.8
1.0

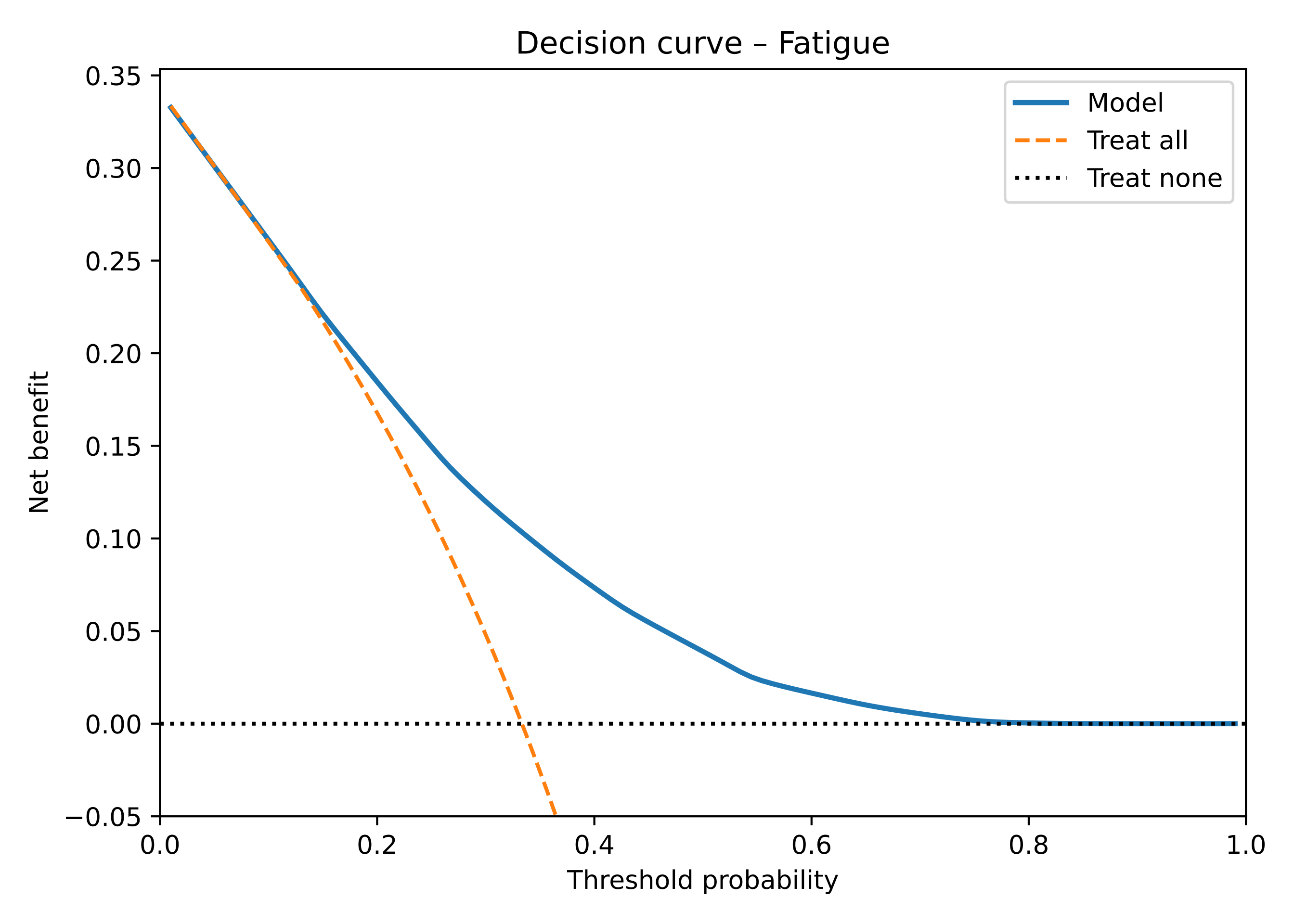
Decision curve – Fatigue
Model
Treat all
Treat none
Net benefit
Threshold probability
0.35
0.30
0.25
0.20
0.15
0.10
0.05
0.00
−0.05
0.0
0.2
0.4
0.6
0.8
1.0

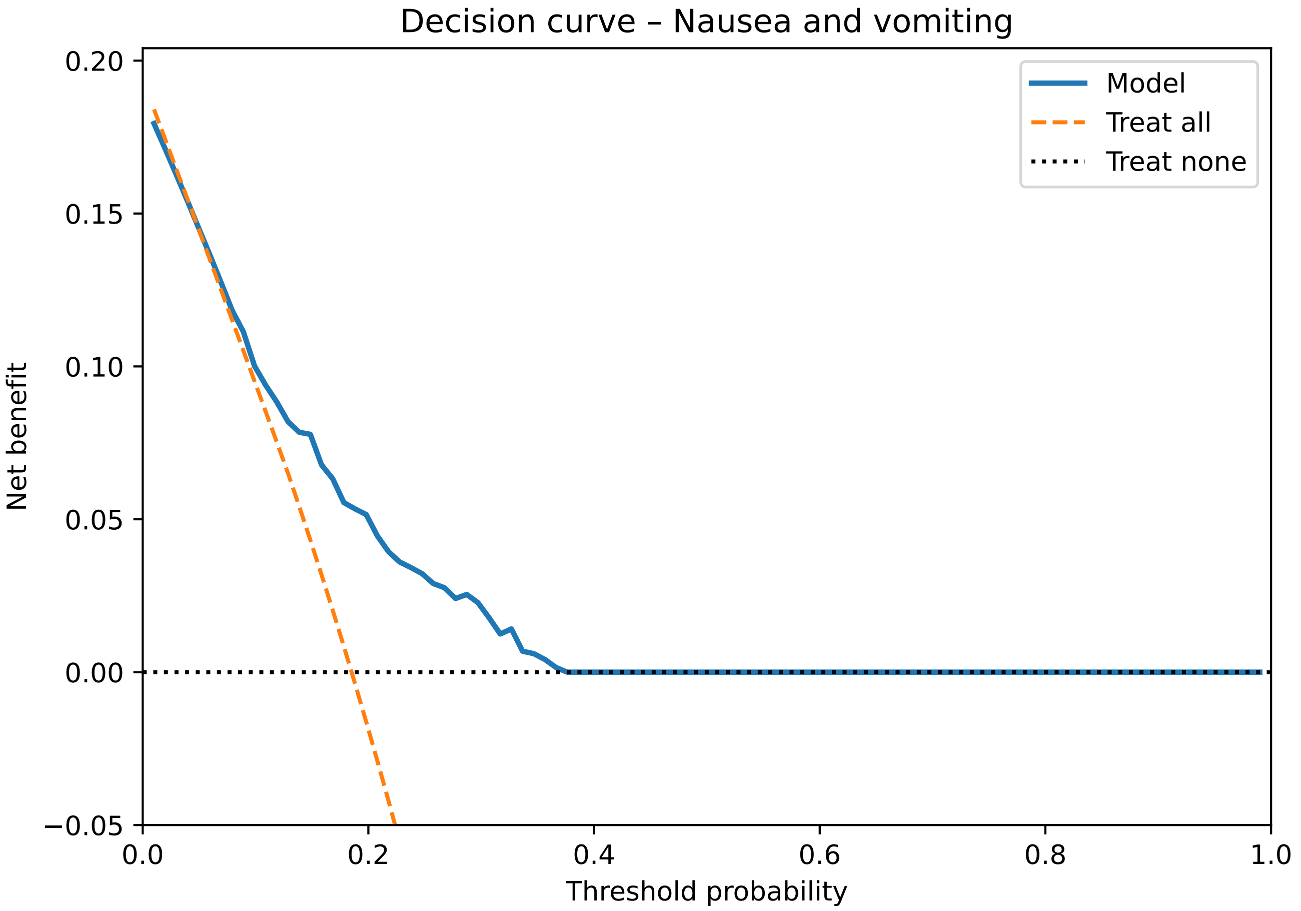

Decision curve – Nausea and vomiting
Model
Treat all
Treat none
Net benefit
Threshold probability
0.20
0.15
0.10
0.05
0.00
−0.05
0.0
0.2
0.4
0.6
0.8
1.0

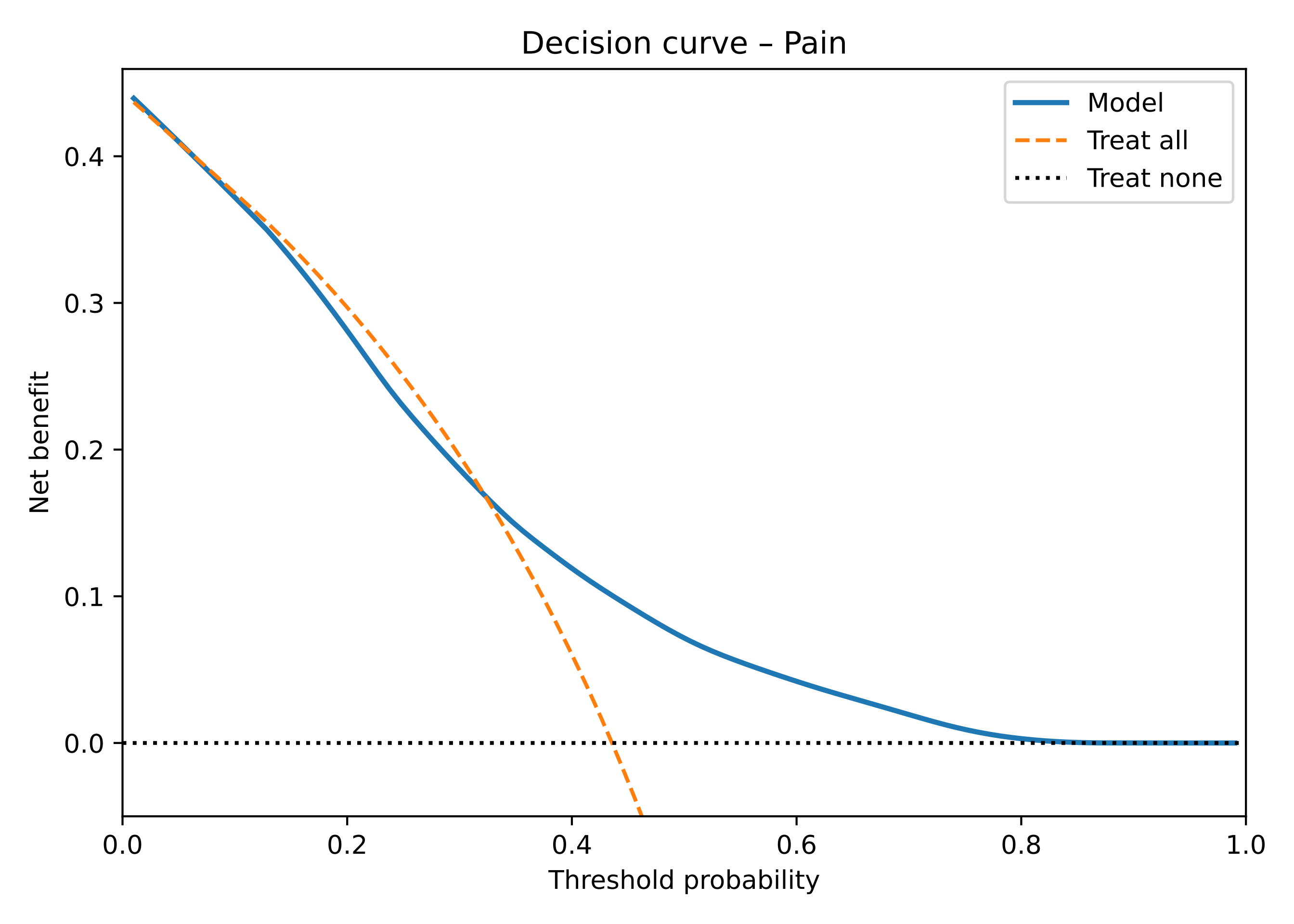
Decision curve – Pain
Model
Treat all
Treat none
Net benefit
Threshold probability
0.0
0.1
0.2
0.3
0.4
0.0
0.2
0.4
0.6
0.8
1.0

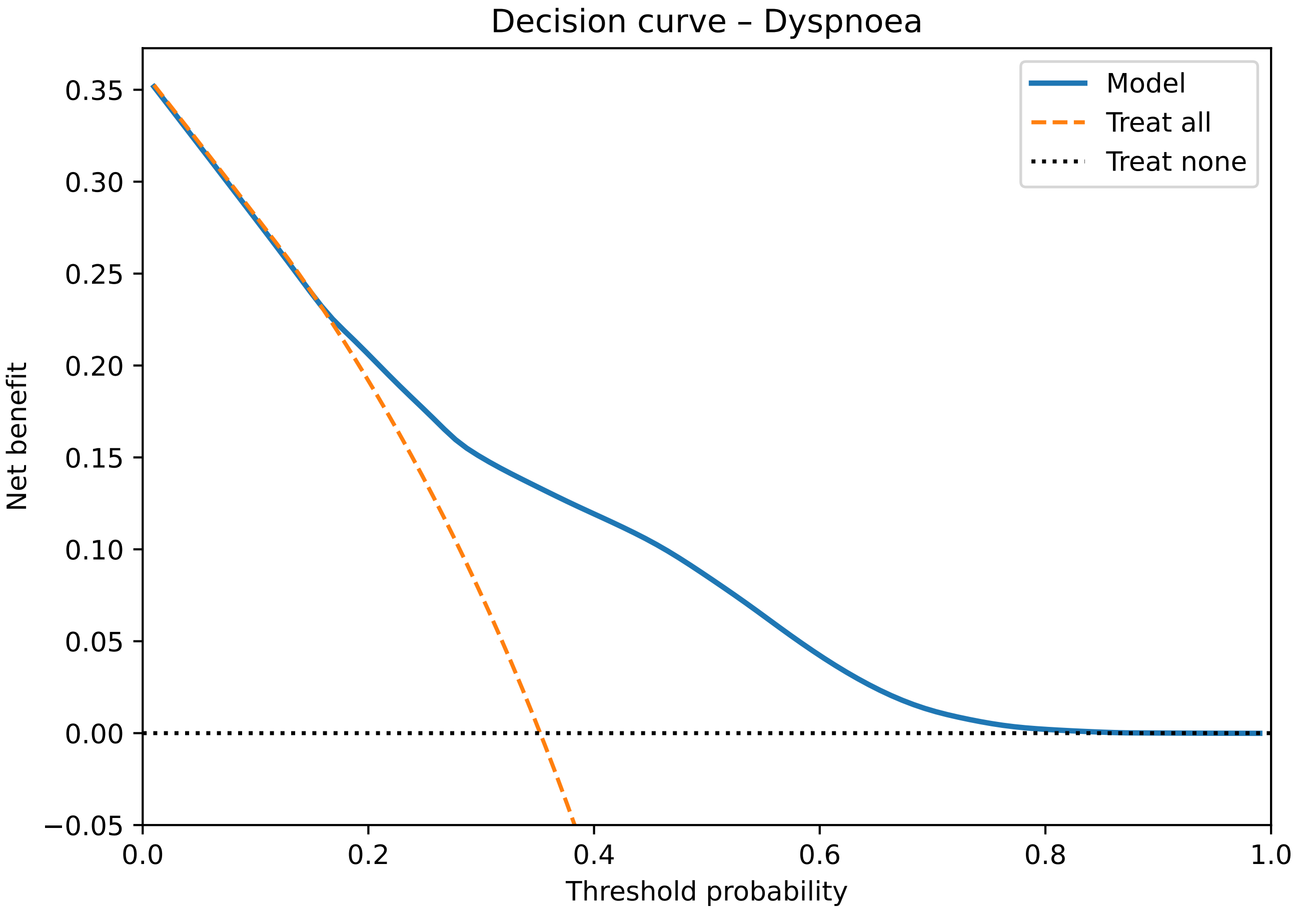

Decision curve – Dyspnoea
Model
Treat all
Treat none
Net benefit
Threshold probability
0.35
0.30
0.25
0.20
0.15
0.10
0.05
0.00
−0.05
0.0
0.2
0.4
0.6
0.8
1.0

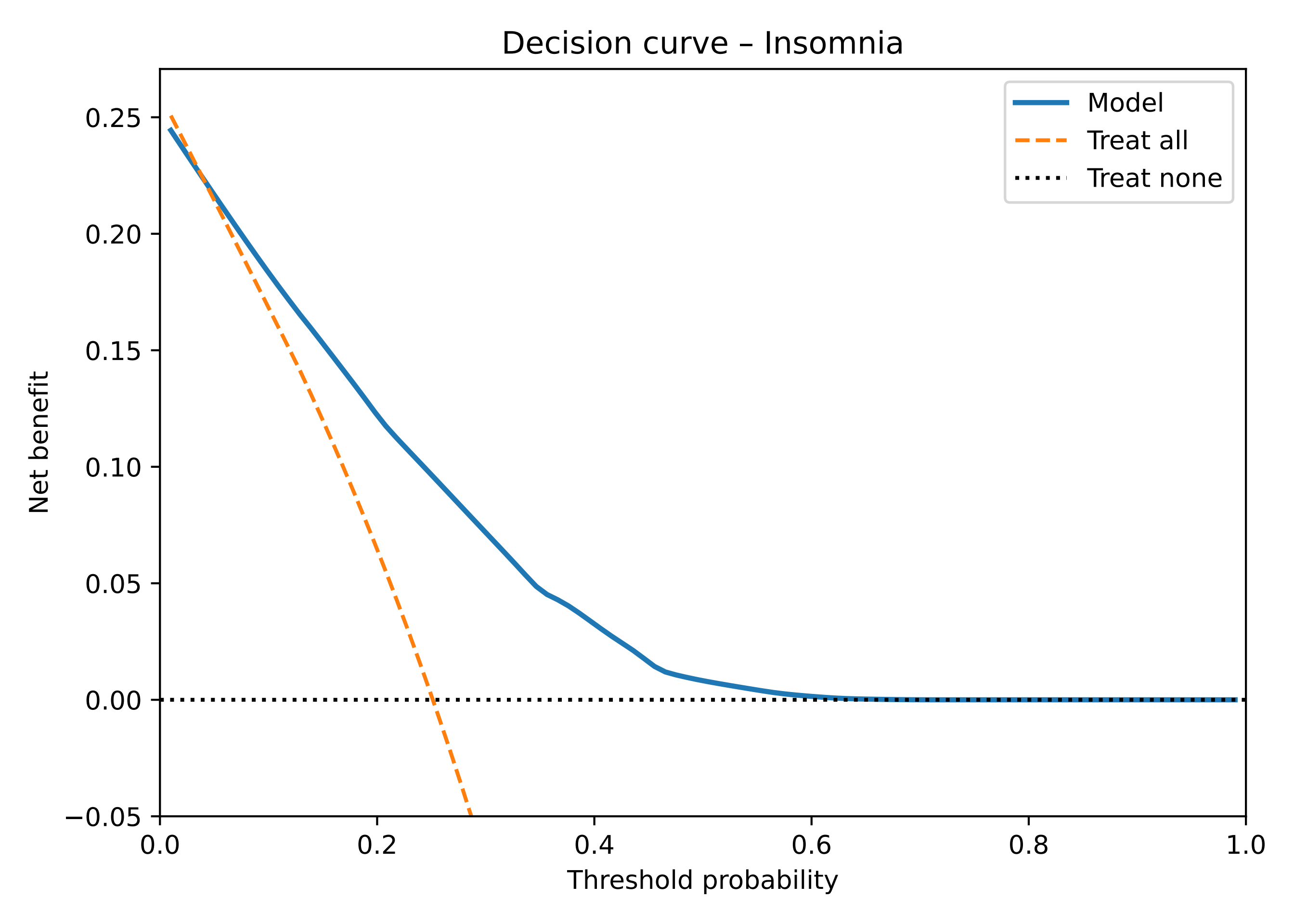

Decision curve – Insomnia
Model
Treat all
Treat none
0.25
0.20
0.15
0.10
0.05
0.00
−0.05
Net benefit
0.0
0.2
0.4
0.6
0.8
1.0
Threshold probability

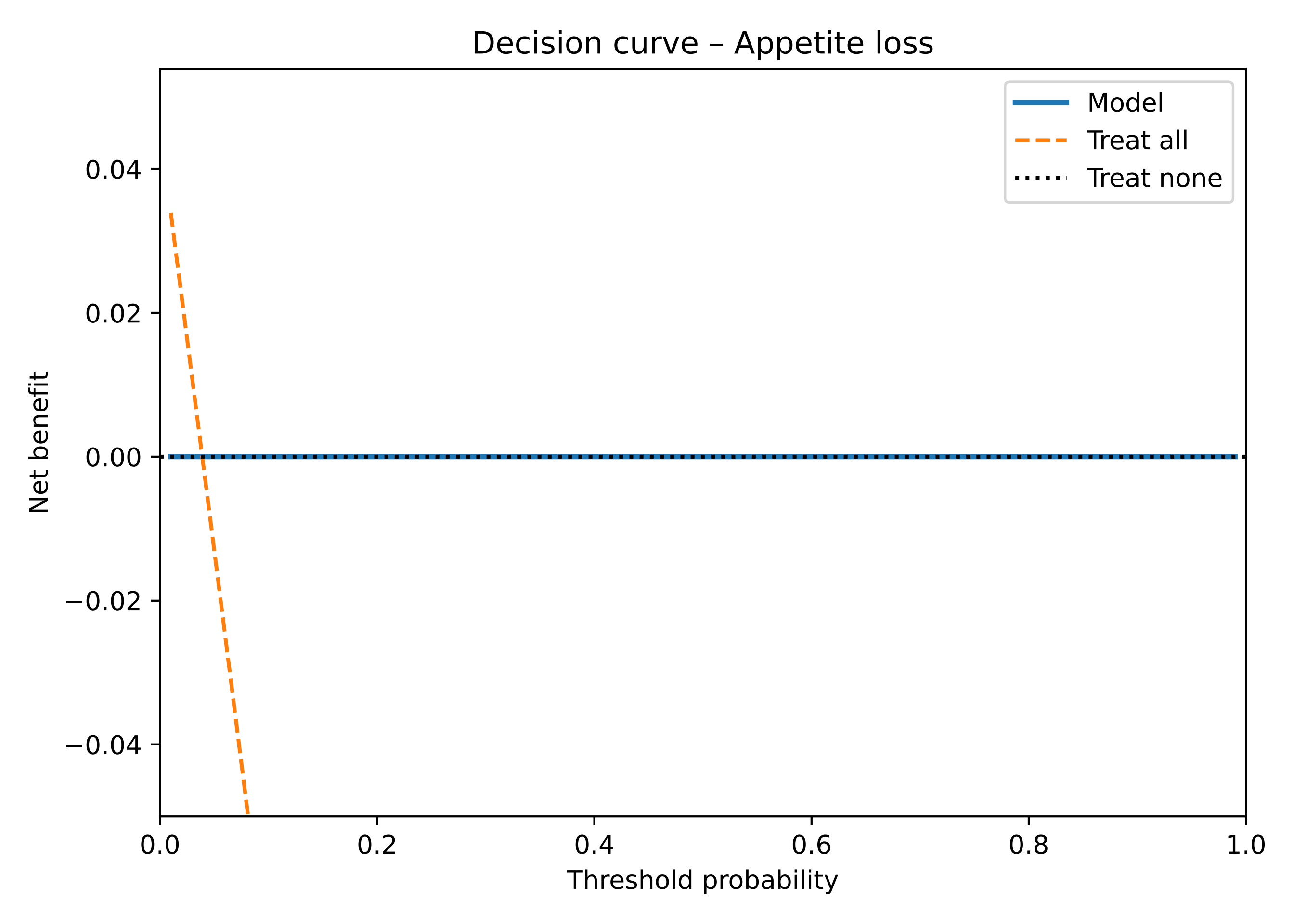

Decision curve – Appetite loss
Model
Treat all
Treat none
0.04
0.02
0.00
−0.02
−0.04
Net benefit
0.0
0.2
0.4
0.6
0.8
1.0
Threshold probability

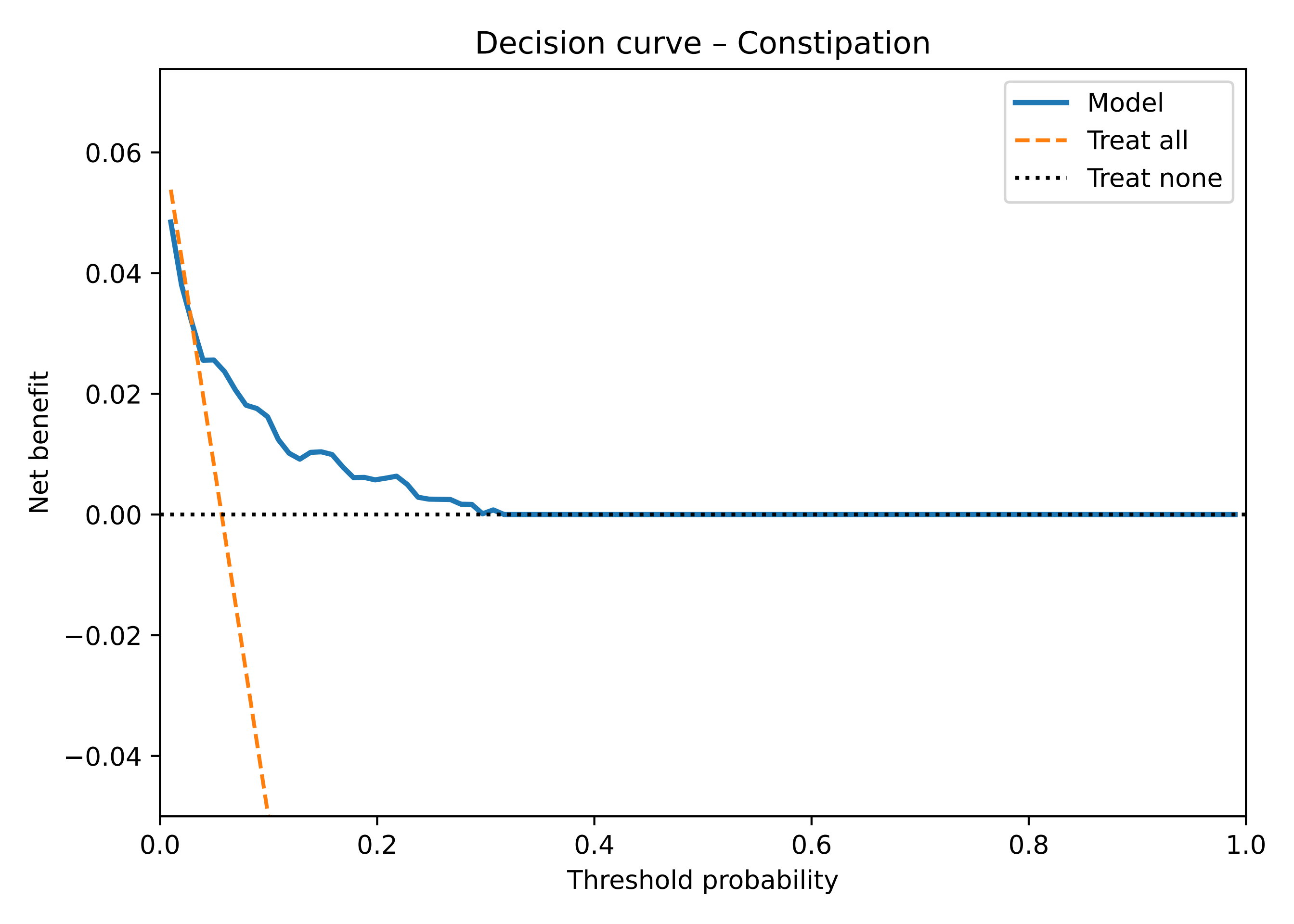
Decision curve – Constipation
Model
Treat all
Treat none
Net benefit
0.06
0.04
0.02
0.00
−0.02
−0.04
0.0
0.2
0.4
0.6
0.8
1.0
Threshold probability

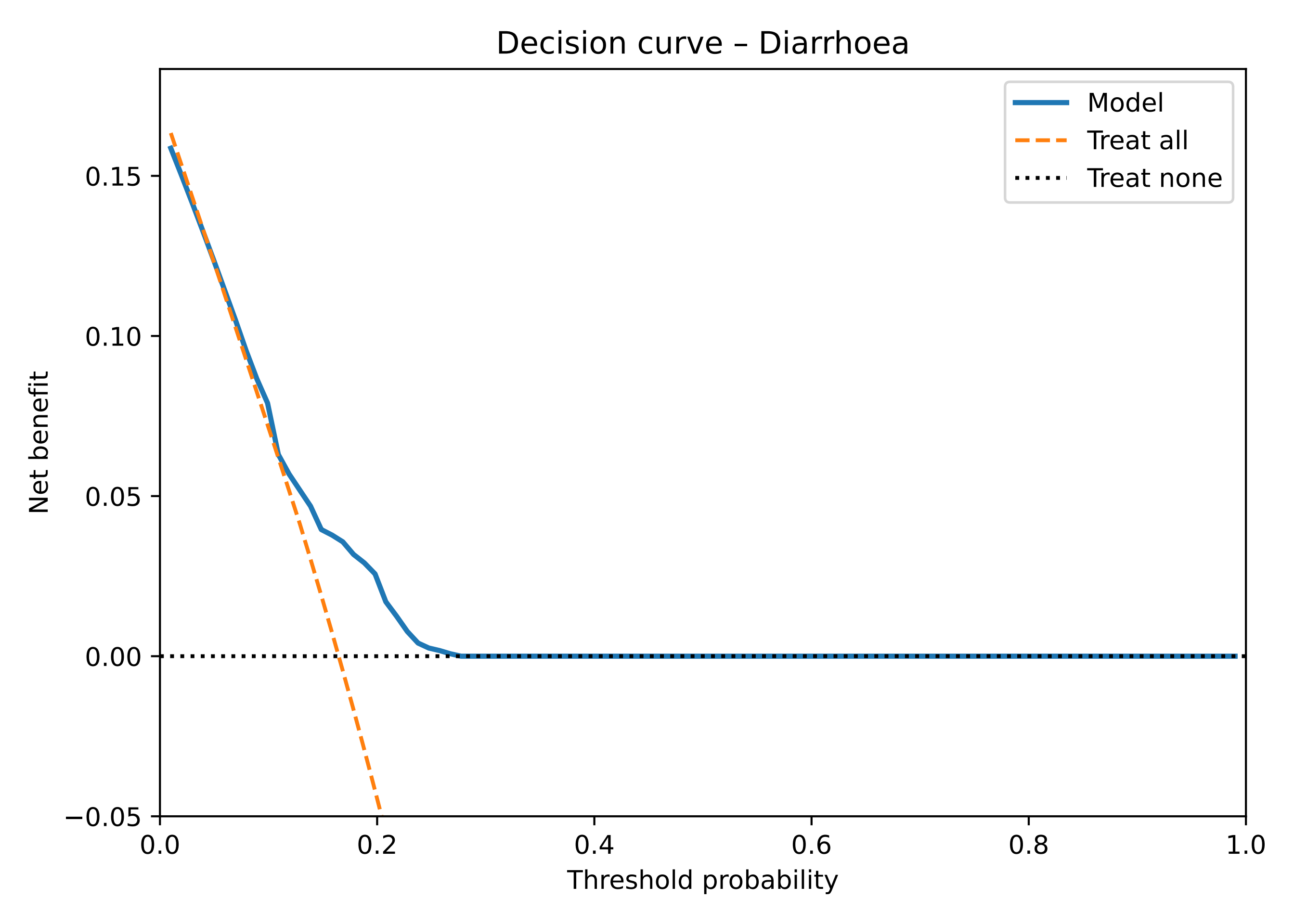

Decision curve – Diarrhoea
Model
Treat all
Treat none
Net benefit
Threshold probability
0.15
0.10
0.05
0.00
−0.05
0.0
0.2
0.4
0.6
0.8
1.0

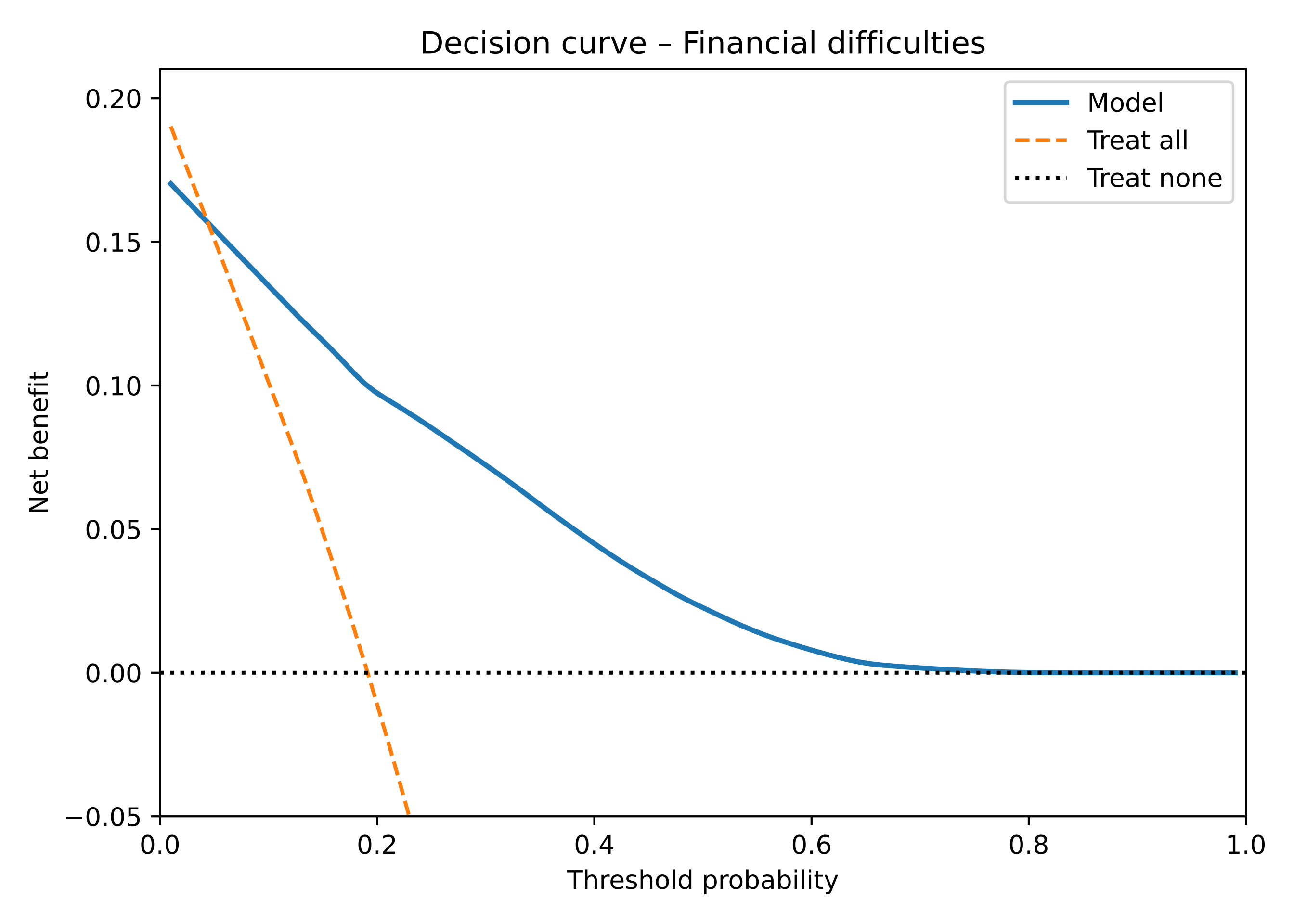

Decision curve – Financial difficulties
Model
Treat all
Treat none
Net benefit
Threshold probability
0.20
0.15
0.10
0.05
0.00
−0.05
0.0
0.2
0.4
0.6
0.8
1.0

Methods S1. List of libraries.

Name	Version
anaconda-anon-usage	0.4.4
anyio	4.2.0
archspec	0.2.3
argon2-cffi	21.3.0
argon2-cffi-bindings	21.2.0
asttokens	2.0.5
async-lru	2.0.4
attrs	23.1.0
babel	2.11.0
beautifulsoup4	4.12.3
bleach	4.1.0
boltons	23.0.0
brotli-python	1.0.9
bzip2	1.0.8
ca-certificates	2024.7.2
certifi	2024.8.30
cffi	1.16.0
charset-normalizer	3.3.2
colorama	0.4.6
comm	0.2.1
conda	24.11.3
conda-content-trust	0.2.0
conda-libmamba-solver	24.7.0
conda-package-handling	2.3.0
conda-package-streaming	0.10.0
console_shortcut_miniconda	0.1.1
contourpy	1.3.0
cryptography	42.0.5
cycler	0.12.1
debugpy	1.6.7
decorator	5.1.1
defusedxml	0.7.1
distro	1.9.0
executing	0.8.3
expat	2.6.2
fmt	9.1.0
fonttools	4.53.1
frozendict	2.4.2
h11	0.14.0
httpcore	1.0.2
httpx	0.27.0
icu	73.1

idna	3.7
imbalanced-learn	0.12.4
imblearn	0
ipykernel	6.28.0
ipython	8.27.0
ipywidgets	8.1.2
jedi	0.19.1
jinja2	3.1.4
joblib	1.4.2
jpeg	9e
json5	0.9.6
jsonpatch	1.33
jsonpointer	2.1
jsonschema	4.19.2
jsonschema-specifications	2023.7.1
jupyter	1.0.0
jupyter-lsp	2.2.0
jupyter_client	8.6.0
jupyter_console	6.6.3
jupyter_core	5.7.2
jupyter_events	0.10.0
jupyter_server	2.14.1
jupyter_server_terminals	0.4.4
jupyterlab	4.2.5
jupyterlab_pygments	0.1.2
jupyterlab_server	2.27.3
jupyterlab_widgets	3.0.10
kiwisolver	1.4.7
krb5	1.20.1
libarchive	3.6.2
libclang	14.0.6
libclang13	14.0.6
libcurl	8.7.1
libffi	3.4.4
libiconv	1.16
libmamba	1.5.8
libmambapy	1.5.8
libpng	1.6.39
libpq	12.17
libsodium	1.0.18
libsolv	0.7.24
libssh2	1.11.0
libxml2	2.13.1
lz4-c	1.9.4
markupsafe	2.1.3
matplotlib	3.9.2

matplotlib-inline	0.1.6
menuinst	2.1.2
mistune	2.0.4
nbclient	0.8.0
nbconvert	7.10.0
nbformat	5.9.2
nest-asyncio	1.6.0
notebook	7.2.2
notebook-shim	0.2.3
numpy	2.1.1
openssl	3.0.15
overrides	7.4.0
packaging	24.1
pandas	2.3.3
pandocfilters	1.5.0
parso	0.8.3
patsy	1.0.1
pcre2	10.42
pillow	10.4.0
pip	24.2
platformdirs	3.10.0
pluggy	1.0.0
ply	3.11
powershell_shortcut_minico	nda 0.0.1
prometheus_client	0.14.1
prompt-toolkit	3.0.43
prompt_toolkit	3.0.43
psutil	5.9.0
pure_eval	0.2.2
pybind11-abi	5
pycosat	0.6.6
pycparser	2.21
pygments	2.15.1
pyparsing	3.1.4
pyqt	5.15.10
pyqt5-sip	12.13.0
pyreadstat	1.2.8
pysocks	1.7.1
python	3.12.4
python-dateutil	2.9.0post0
python-fastjsonschema	2.16.2
python-json-logger	2.0.7
pytz	2024.1
pywin32	305
pywinpty	2.0.10
pyyaml	6.0.1

pyzmq	25.1.2
qt-main	5.15.2
qtconsole	5.5.1
qtpy	2.4.1
referencing	0.30.2
reproc	14.2.4
reproc-cpp	14.2.4
requests	2.32.3
rfc3339-validator	0.1.4
rfc3986-validator	0.1.1
rpds-py	0.10.6
ruamel.yaml	0.17.21
scikit-learn	1.8.0
scipy	1.14.1
seaborn	0.13.2
send2trash	1.8.2
setuptools	72.1.0
sip	6.7.12
six	1.16.0
sniffio	1.3.0
soupsieve	2.5
sqlite	3.45.3
stack_data	0.2.0
statsmodels	0.14.4
terminado	0.17.1
threadpoolctl	3.5.0
tinycss2	1.2.1
tk	8.6.14
tkintermodernthemes	1.10.2
tornado	6.4.1
tqdm	4.66.4
traitlets	5.14.3
truststore	0.8.0
typing-extensions	4.11.0
typing_extensions	4.11.0
tzdata	2024.1
urllib3	2.2.2
vc	14.4
vs2015_runtime	14.40.33807
wcwidth	0.2.5
webencodings	0.5.1
websocket-client	1.8.0
wheel	0.43.0
widgetsnbextension	4.0.10
win_inet_pton	1.1.0
winpty	0.4.3

xgboost	2.1.3
xz	5.4.6
yaml	0.2.5
yaml-cpp	0.8.0
zeromq	4.3.5
zlib	1.2.13
zstandard	0.22.0
zstd	1.5.5

Methods S2. **Codebook**.

Categories	Variable	Description	Type	Values/Range
**Patient and source identification**	BALANCE_ID	BALANCE study identification code with the first two digits identifying the source	Numerical	1000000-9999999
	RWD	Data from clinical routine or research	Categorical	0=Research, 1=Clinical routine
	Source	Origin of dataset	Categorical	1-99
**Patient characteristics**	Sex	Sex at birth	Categorical	0=Male, 1=Female
	Country	Country of origin / assessment location	Categorical	text
	Year_of_birth	Year of birth	Date	≥ 1900
	Age_TX	Age in years at time point	Numerical	> 0
	Height	Height in cm at baseline, max. two decimal digits	Numerical	> 0
	Weight_T0	Weight in kg at baseline, max. two decimal digits	Numerical	> 0
	BMI_T0	BMI as kg/m² at baseline	Numerical	> 0
	Menopause	Menopause at baseline	Categorical	0=Pre/perimenopausal,<br>1=Postmenopausal / artificial
	Marital_status	Marital status at baseline	Categorical	1=Married/Living Together,<br>2=Relationship (not married/not living together),<br>3=Divorced/Split up,<br>4= Widow(er)/Partner deceased,<br>5= Never married/lived together
	Education_status	Education status at baseline	Categorical	1=Primary education or lower,<br>2=Secondary educationor similar,<br>3=Vocational education or similar,<br>4=University, Higher (vocational) education
	Vital_status	Vital status at last contact	Categorical	0=Deceased, 1=Alive
	Survival_duration	Duration of survival since diagnosis in days at last contact	Numerical	> 0
**Comorbidities**	Comorbidities	How many comorbidities are present	Numerical	0,1, =>2
	Cardiac_history	Has there been any cardiac issues or illnesses in the last 12 months?	Categorical	0=no, 1=yes
	Stroke_history	Has there been any stroke in the last 12 months?	Categorical	0=no, 1=yes
	BP_history	Has there been any bloodpressure issues or illesses in the last 12 months?	Categorical	0=no, 1=yes
	Respitory_history	Has there been any respitory issues or illnesses in the last 12 months?	Categorical	0=no, 1=yes
	Diabetes	Has there been any diabetes present or diagnosed in the last 12 months?	Categorical	0=no, 1=yes
	Stomach_ulcer	Has there been stomach ulcer(s) in the last 12 months?	Categorical	0=no, 1=yes
	Kidney_history	Has there been any kidney issues or illnesses in the last 12 months?	Categorical	0=no, 1=yes
	Liver_history	Has there been any liver issues or illnesses in the last 12 months?	Categorical	0=no, 1=yes
	Blood_history	Has there been any blood issues or illnesses in the last 12 months?	Categorical	0=no, 1=yes
	Thyriod_history	Has there been any thyroid issues or illnesses in the last 12 months?	Categorical	0=no, 1=yes
	Depression	Has there been depression diagnosed or present in the last 12 months?	Categorical	0=no, 1=yes
	Arthritis	Has there been any arthritis diagnosed or present in the last 12 months?	Categorical	0=no, 1=yes
	Backpain	Has there been any back pain issues in the last 12 months?	Categorical	0=no, 1=yes
	Jointpain	Has there been any joint pain issues in the last 12 months?	Categorical	0=no, 1=yes
**Disease characteristics**	Tumour_No	Tumour follow number	Numerical	> 0
	Location_code	ICD-10 coding of location of tumour (topography)	Categorical	1=C50.0 (Nipple),<br>2=C50.1 (Central portion),<br>3=C50.2 (Upper inner quadrant),<br>4=C50.3 (Lower inner quadrant),<br>5=C50.4 (Upper outer quadrant),<br>6=C50.5 (Lower outer quadrant),<br>7=C50.6 (Axillary tail),<br>8=C50.8 (Overlapping lesion of breast)
	Lateralisation	Left or right breast	Categorical	1= left,<br>2=right,<br>3=both
	Diag_code	6 digit type of tumour code according to the ICD-O-3 criteria	Categorical	100000-999999
	Histology_code	4 digit histology code according to the ICD-O-3 criteria	Categorical	1000-9999
	Behaviour_code	1 digit behaviour code according to ICD-O-3 criteria	Categorical	0= Benign,<br>1=Uncertain whether benign or malignant,<br>2=Carcinoma in situ,<br>3=Malignant, primary site,<br>6= Malignant, metastatic site,<br>9=Malignant, uncertain whether primary or metastatic site

	Differentation_grade	1 digit differentation code according to ICD-O-3 criteria	Categorical	1=Grade I Well differentiated,<br>2=Grade II Moderately differentiated,<br>3=Grade III Poorly differentiated,<br>4=Grade IV Undifferentiated
	T_staging	UICC staging for malignant tumours, T describes size original tumour	Categorical	X =cannot be measured,<br>0=main tumor not found,<br>1.1=Carcinoma in situ,<br>1=T1 tumour diameter <= 2cm ,<br>2=T2 tumour diameter 2-5cm.<br>3=T3 tumour diameter>5cm,<br>4=T4 tumour has invaded other organs
	N_staging	UICC staging for malignant tumours, N describes if tumour spread to surrounding lymphnodes	Categorical	X=unable to evalulate, 0=No spread to lymphnodes,<br>1=spread to <=3 lympnodes of underam or any of internal mammary lymphnodes,<br>1.1 = N1mi (micrometastases),<br>2=spread to 4-9 lymphnodes of the underarm<br>OR has enlarged the lymphnodes of internal mammary area, 3=spread to 10 or more lymphnodes
	M_staging	UICC staging for malignant tumours, M describes if tumour has metastasised	Categorical	0=no, 1=yes
	Stage	Stage derived from TNM staging	Categorical	0 = Stage 0,<br>1 = Stage IA,<br>2 = Stage IB,<br>3 = Stage IIA,<br>4 = Stage IIB,<br>5 = Stage IIIA,<br>6 = Stage IIIB,<br>7 = Stage IIIC,<br>8 = Stage IV
	ER_status	Expression of the estrogen receptor for sensitivity in percentage	Numerical	0-100
	ER_stat_cat	If estrogen sensitivity is present, ER_status categorised	Categorical	0=no, 1=yes
	PR_status	Expression of the progesteron receptor for sensitivity in percentage	Numerical	0-100
	PR_stat_cat	If progesteron sensitivity is present, PR_status categorised	Categorical	0=no, 1=yes
	Her2Neu_status	If the tumour expresses Her2Neu receptors	Ordinal	0=no coloration or less than 10% tumour cells incomplete or weak coloration,<br>1=More than 10% of tumour cells colors incomplete or weak,<br>2=More than 10% of tumour cells show moderate or incomplete coloring, or complete and intense coloring in =<10% of cells,<br>3=more than 10% of cells show circumfetional and strong color
	T_staging_postOP	UICC staging for malignant tumours, T describes size tumour after surgery	Ordinal	X =cannot be measured,<br>0=main tumor not found,<br>1.1=Carcinoma in situ, 1=T1 tumour diameter <= 2cm,<br>2=T2 tumour diameter 2-5cm,<br>3=T3 tumour diameter>5cm,<br>4=T4 tumour has invaded other organs
	N_staging_postOP	UICC staging for malignant tumours, N describes tumour spread to surrounding lymphnodes after surgery	Ordinal	X=unable to evalulate,<br>0=No spread to lymphnodes,<br>1=spread to <=3 lympnodes of underam or any of internal mammary lymphnodes,<br>1.1 = N1mi (micrometastases),<br>2=spread to 4-9 lymphnodes of the underarm OR has enlarged the lymphnodes of internal mammary area,<br>3=spread to 10 or more lymphnodes
	M_staging_postOP	UICC staging for malignant tumours, M describes if tumour has metastasised assesed after surgery	Ordinal	0=no, 1=yes
	Stage_postOP	Stage derived from TNM staging assesed after post surgery	Ordinal	0 = Stage 0,<br>1 = Stage IA,<br>2 = Stage IB,<br>3 = Stage IIA,<br>4 = Stage IIB,<br>5 = Stage IIIA,<br>6 = Stage IIIB,<br>7 = Stage IIIC,<br>8 = Stage IV
**Treatment characteristics**	Surgery	If patient received surgery	Categorical	0=no, 1=yes
	Radiotherapy	If patient received radiotherapy	Categorical	0=no, 1=yes
	Neo_RT	If radiotherapy is received as neo-adjuvant treatment	Categorical	0=no, 1=yes
	Chemotherapy	If patient received chemotherapy	Categorical	0=no, 1=yes
	Neo_Chemo	If chemotherapy is received as neo-adjuvant treatment	Categorical	0=no, 1=yes
	Immunotherapy	If patient received immunotherapy	Categorical	0=no, 1=yes
	Neo_Immuno	If immunotherapy is received as neo-adjuvant treatment	Categorical	0=no, 1=yes
	Targeted	If patient received targeted therapy	Categorical	0=no, 1=yes
	Neo_Targeted	If targeted therapy is received as neo-adjuvant treatment	Categorical	0=no, 1=yes
	Hormonaltherapy	If patient received hormonal therapy	Categorical	0=no, 1=yes
	Neo_Hormonal	If hormonal therapy is received as neo-adjuvant treatment	Categorical	0=no, 1=yes

	Treatment_n	Type of treatment number n received (in chronological order)	Categorical	1 = Chemotherapy,<br>2 = Immunotherapy,<br>3 = Radiotherapy,<br>4 = Surgery,<br>5 = Hormonaltherapy,<br>6 = Targeted Therapy,<br>9 = Other
	Start_date_TRn	Start date of treatment number n in days since diagnosis	Numerical	≥ 0
	Stop_date_TRn	Stop date of treatment number n in days since diagnosis	Numerical	≥ 0
**QoL Assessment**	Assessment_date	Time of QoL assessment in months since diagnosis	Numerical	≥ 0
	Assessment_date_days	Time of QoL assessment in days since diagnosis	Numerical	≥ 0
**EORTC-QLG-C30 items**	C30_Q1	Do you have any trouble doing strenuous activities, like carrying a heavy shopping bag or a suitcase?	Ordinal	1=not at all, 2=a little, 3=quite a bit, 4=very much
	C30_Q2	Do you have any trouble taking a long walk?	Ordinal	1=not at all, 2=a little, 3=quite a bit, 4=very much
	C30_Q3	Do you have any trouble taking a short walk outside of the house?	Ordinal	1=not at all, 2=a little, 3=quite a bit, 4=very much
	C30_Q4	Do you need to stay in bed or a chair during the day?	Ordinal	1=not at all, 2=a little, 3=quite a bit, 4=very much
	C30_Q5	Do you need help with eating, dressing, washing yourself or using the toilet?	Ordinal	1=not at all, 2=a little, 3=quite a bit, 4=very much
	C30_Q6	Were you limited in doing either your work or other daily activities?	Ordinal	1=not at all, 2=a little, 3=quite a bit, 4=very much
	C30_Q7	Were you limited in pursuing your hobbies or other leisure time activities?	Ordinal	1=not at all, 2=a little, 3=quite a bit, 4=very much
	C30_Q8	Were you short of breath?	Ordinal	1=not at all, 2=a little, 3=quite a bit, 4=very much
	C30_Q9	leisure time activities?	Ordinal	1=not at all, 2=a little, 3=quite a bit, 4=very much
	C30_Q10	Did you need to rest?	Ordinal	1=not at all, 2=a little, 3=quite a bit, 4=very much
	C30_Q11	Have you had trouble sleeping?	Ordinal	1=not at all, 2=a little, 3=quite a bit, 4=very much
	C30_Q12	Have you felt weak?	Ordinal	1=not at all, 2=a little, 3=quite a bit, 4=very much
	C30_Q13	Have you lacked appetite?	Ordinal	1=not at all, 2=a little, 3=quite a bit, 4=very much
	C30_Q14	Have you felt nauseated?	Ordinal	1=not at all, 2=a little, 3=quite a bit, 4=very much
	C30_Q15	Have you vomited?	Ordinal	1=not at all, 2=a little, 3=quite a bit, 4=very much
	C30_Q16	Have you been constipated?	Ordinal	1=not at all, 2=a little, 3=quite a bit, 4=very much
	C30_Q17	Have you had diarrhea?	Ordinal	1=not at all, 2=a little, 3=quite a bit, 4=very much
	C30_Q18	Were you tired?	Ordinal	1=not at all, 2=a little, 3=quite a bit, 4=very much
	C30_Q19	Did pain interfere with your daily activities?	Ordinal	1=not at all, 2=a little, 3=quite a bit, 4=very much
	C30_Q20	Have you had difficulty in concentrating on things, like reading a newspaper or watching television?	Ordinal	1=not at all, 2=a little, 3=quite a bit, 4=very much
	C30_Q21	Did you feel tense?	Ordinal	1=not at all, 2=a little, 3=quite a bit, 4=very much
	C30_Q22	Did you worry?	Ordinal	1=not at all, 2=a little, 3=quite a bit, 4=very much
	C30_Q23	Did you feel irritable?	Ordinal	1=not at all, 2=a little, 3=quite a bit, 4=very much
	C30_Q24	Did you feel depressed?	Ordinal	1=not at all, 2=a little, 3=quite a bit, 4=very much
	C30_Q25	Have you had difficulty remembering things?	Ordinal	1=not at all, 2=a little, 3=quite a bit, 4=very much
	C30_Q26	Has your physical condition or medical treatment interfered with your family life?	Ordinal	1=not at all, 2=a little, 3=quite a bit, 4=very much
	C30_Q27	Has your physical condition or medical treatment interfered with your social activities?	Ordinal	1=not at all, 2=a little, 3=quite a bit, 4=very much
	C30_Q28	Has your physical condition or medical treatment caused you financial difficulties?	Ordinal	1=not at all, 2=a little, 3=quite a bit, 4=very much
	C30_Q29	How would you rate your overall health during the past week?	Ordinal	1-7
	C30_Q30	How would you rate your overall quality of life during the past week?	Ordinal	1-7
**EORTC-QLQ-C30 scales**	C30_QL2	Global health status/QoL scale	Numerical	0-100
	C30_PF2	Physical functioning	Numerical	0-100
	C30_RF2	Role functioning	Numerical	0-100
	C30_EF	Emotional functioning	Numerical	0-100
	C30_CF	Cognitive functioning	Numerical	0-100
	C30_SF	Social functioning	Numerical	0-100
	C30_FA	Fatigue	Numerical	0-100
	C30_NV	Nausea and vomiting	Numerical	0-100
	C30_PA	Pain	Numerical	0-100
	C30_DY_	Dyspnoea	Numerical	0-100
	C30_SL	Insomnia	Numerical	0-100
	C30_AP	Appetite loss	Numerical	0-100
	C30_CO	Constipation	Numerical	0-100
	C30_DI	Diarrhoea	Numerical	0-100
	C30_FI	Financial difficulties	Numerical	0-100
**EORTC-QLQ-BR23 items**	BR23_Q31	Did you have a dry mouth?	Ordinal	1=not at all, 2=a little, 3=quite a bit, 4=very much
	BR23_Q32	Did food and drink taste different than usual?	Ordinal	1=not at all, 2=a little, 3=quite a bit, 4=very much
	BR23_Q33	Were your eyes painful, irritated or watery?	Ordinal	1=not at all, 2=a little, 3=quite a bit, 4=very much
	BR23_Q34	Have you lost any hair?	Ordinal	1=not at all, 2=a little, 3=quite a bit, 4=very much
	BR23_Q35	Answer this question only if you had any hair loss: Were you upset by the loss of your hair?	Ordinal	1=not at all, 2=a little, 3=quite a bit, 4=very much
	BR23_Q36	Did you feel ill or unwell?	Ordinal	1=not at all, 2=a little, 3=quite a bit, 4=very much
	BR23_Q37	Did you have hot flushes?	Ordinal	1=not at all, 2=a little, 3=quite a bit, 4=very much

	BR23_Q38	Did you have headaches?	Ordinal	1=not at all, 2=a little, 3=quite a bit, 4=very much
	BR23_Q39	Have you felt physically less attractive as a result of your disease or treatment?	Ordinal	1=not at all, 2=a little, 3=quite a bit, 4=very much
	BR23_Q40	Have you been feeling less feminine as a result of your disease or treatment?	Ordinal	1=not at all, 2=a little, 3=quite a bit, 4=very much
	BR23_Q41	Did you find it difficult to look at yourself naked?	Ordinal	1=not at all, 2=a little, 3=quite a bit, 4=very much
	BR23_Q42	Have you been dissatisfied with your body?	Ordinal	1=not at all, 2=a little, 3=quite a bit, 4=very much
	BR23_Q43	Were you worried about your health in the future?	Ordinal	1=not at all, 2=a little, 3=quite a bit, 4=very much
	BR23_Q44	To what extent were you interested in sex?	Ordinal	1=not at all, 2=a little, 3=quite a bit, 4=very much
	BR23_Q45	To what extent were you sexually active? (with or without intercourse)	Ordinal	1=not at all, 2=a little, 3=quite a bit, 4=very much
	BR23_Q46	Answer this question only if you have been sexually active: To what extent was sex enjoyable for you?	Ordinal	1=not at all, 2=a little, 3=quite a bit, 4=very much
	BR23_Q47	Did you have any pain in your arm or shoulder?	Ordinal	1=not at all, 2=a little, 3=quite a bit, 4=very much
	BR23_Q48	Did you have a swollen arm or hand?	Ordinal	1=not at all, 2=a little, 3=quite a bit, 4=very much
	BR23_Q49	Was it difficult to raise your arm or to move it sideways?	Ordinal	1=not at all, 2=a little, 3=quite a bit, 4=very much
	BR23_Q20	Have you had any pain in the area of your affected breast?	Ordinal	1=not at all, 2=a little, 3=quite a bit, 4=very much
	BR23_Q51	Was the area of your affected breast swollen?	Ordinal	1=not at all, 2=a little, 3=quite a bit, 4=very much
	BR23_Q52	Was the area of your affected breast oversensitive?	Ordinal	1=not at all, 2=a little, 3=quite a bit, 4=very much
	BR23_Q53	Have you had skin problems on or in the area of your affected breast (e.g., itchy, dry, flaky)?	Ordinal	1=not at all, 2=a little, 3=quite a bit, 4=very much
**EORTC-QLQ-BR23 scales**	BR23_ST	Systemic Therapy Side Effects	Numerical	0-100
	BR23_HL	Upset by Hair Loss	Numerical	0-100
	BR23_AS	Arm Symptoms	Numerical	0-100
	BR23_BS	Breast Symptoms	Numerical	0-100
	BR23_BI	Body Image	Numerical	0-100
	BR23_FU	Future Perspective	Numerical	0-100
	BR23_SEF	Sexual Functioning	Numerical	0-100
	BR23_SEE	Sexual Enjoyment	Numerical	0-100

Methods S3. Outcome pairing.

To evaluate time-dynamic predictive performance, for each patient, all possible combinations of their assessments were compiled into distinct pairs. A new variable was introduced to represent the difference in days between the assessment dates within each pair. The outcome variable for each pair was defined as the value from the later assessment. For example, if a patient had three assessments, A1, A2, and A3, occurring at different time points, pairs such as (A1, A2), (A1, A3), and (A2, A3) were generated.

Each pair (A_m, A_n) is represented as a training sample (X_m, t_{m-n}, Y_n), where X_m are the features of the assessment m, Y_n is the binary outcome variable of the respective observation, and t_{m-n} is the time difference between A_m and A_n in days. Each training sample can be interpreted as "t_{m-n} days after the patient has reported results X_m, the patient's outcome is Y_n.”.

In the example below the Pair A1+A2 contains A1’s C30_Q1 answer (=1, red circle) as a predictor and A2’s C30_FA domain score (=50, yellow circle) as the target (outcome).

Assessment	Date	C30_Q1	...	C30_FA
A1	01.01.2001	1	...	33
A2	15.01.2001	2	...	50
A3	30.01.2001	3	...	66

Pair	C30_Q1 (Predictor)	Time Difference	...	C30_FA (Target)
A1 + A2	1	14		50
A1 + A3	1	29		66
A2 + A3	2	15		66
